# Supplementary figures and images for: Protocell formation on micrometeorites
Source: Sci Rep. 2026 Jul 11;16:21833. doi: 10.1038/s41598-026-60022-x (PMC13365509; doi:10.1038/s41598-026-60022-x)

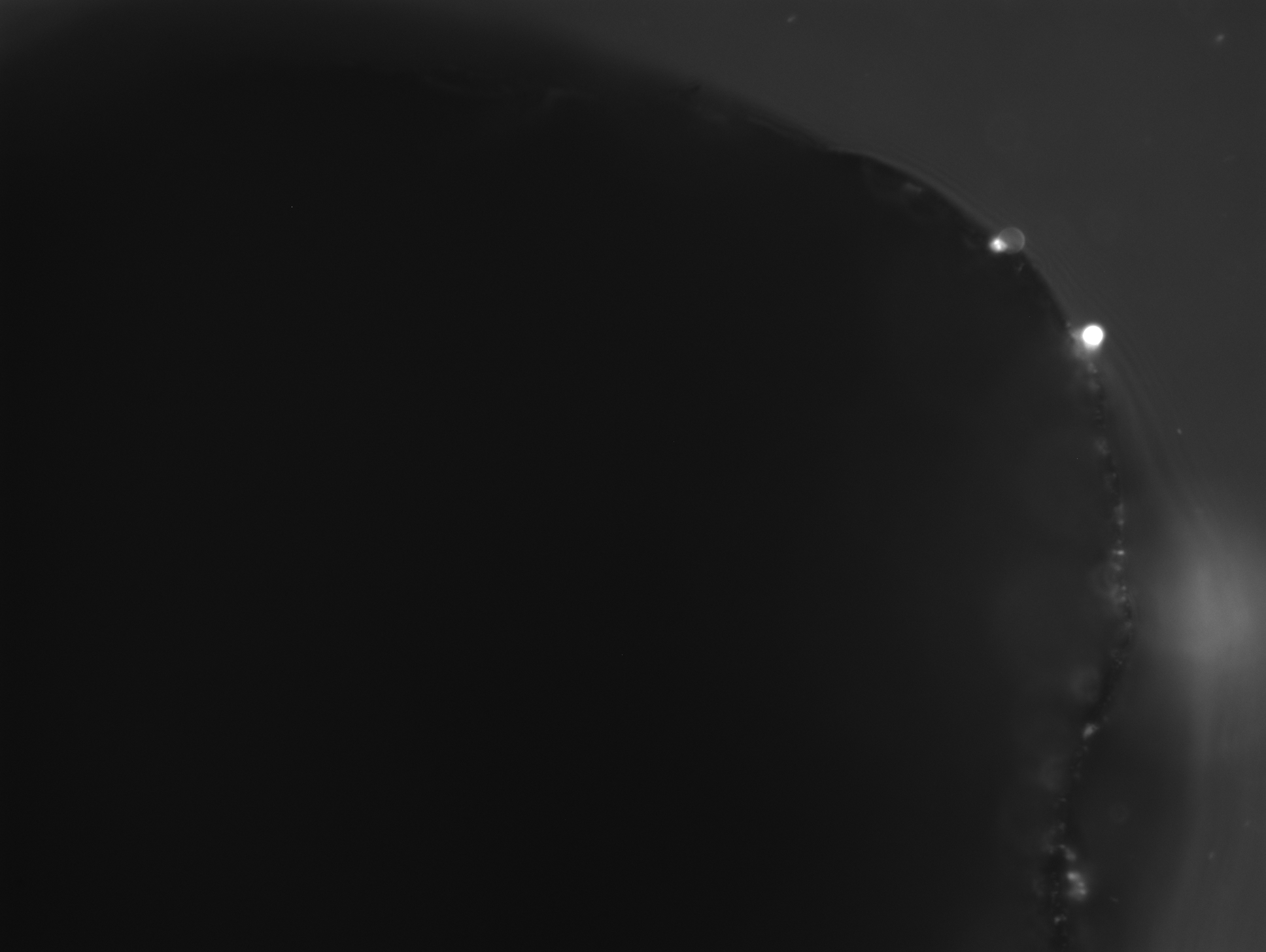

Supplement: Supplementary file 1 — Supplementary Material 1 [file 41598_2026_60022_MOESM1_ESM.zip › SI_Fig5_source images/A1_s1/1_23_44.971.tif]

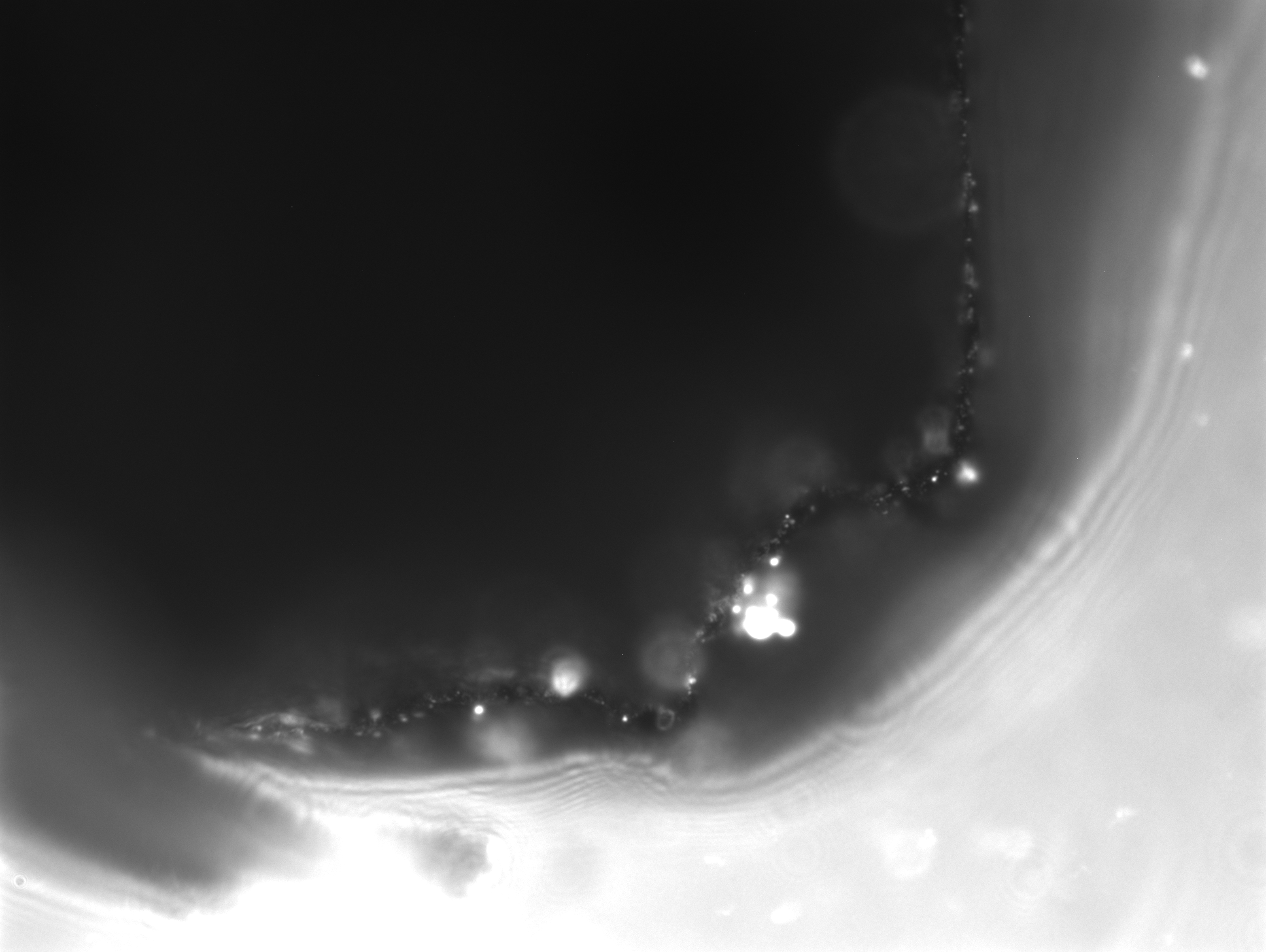

Supplement: Supplementary file 1 — Supplementary Material 1 [file 41598_2026_60022_MOESM1_ESM.zip › SI_Fig5_source images/A1_s1/2_66_06.370.tif]

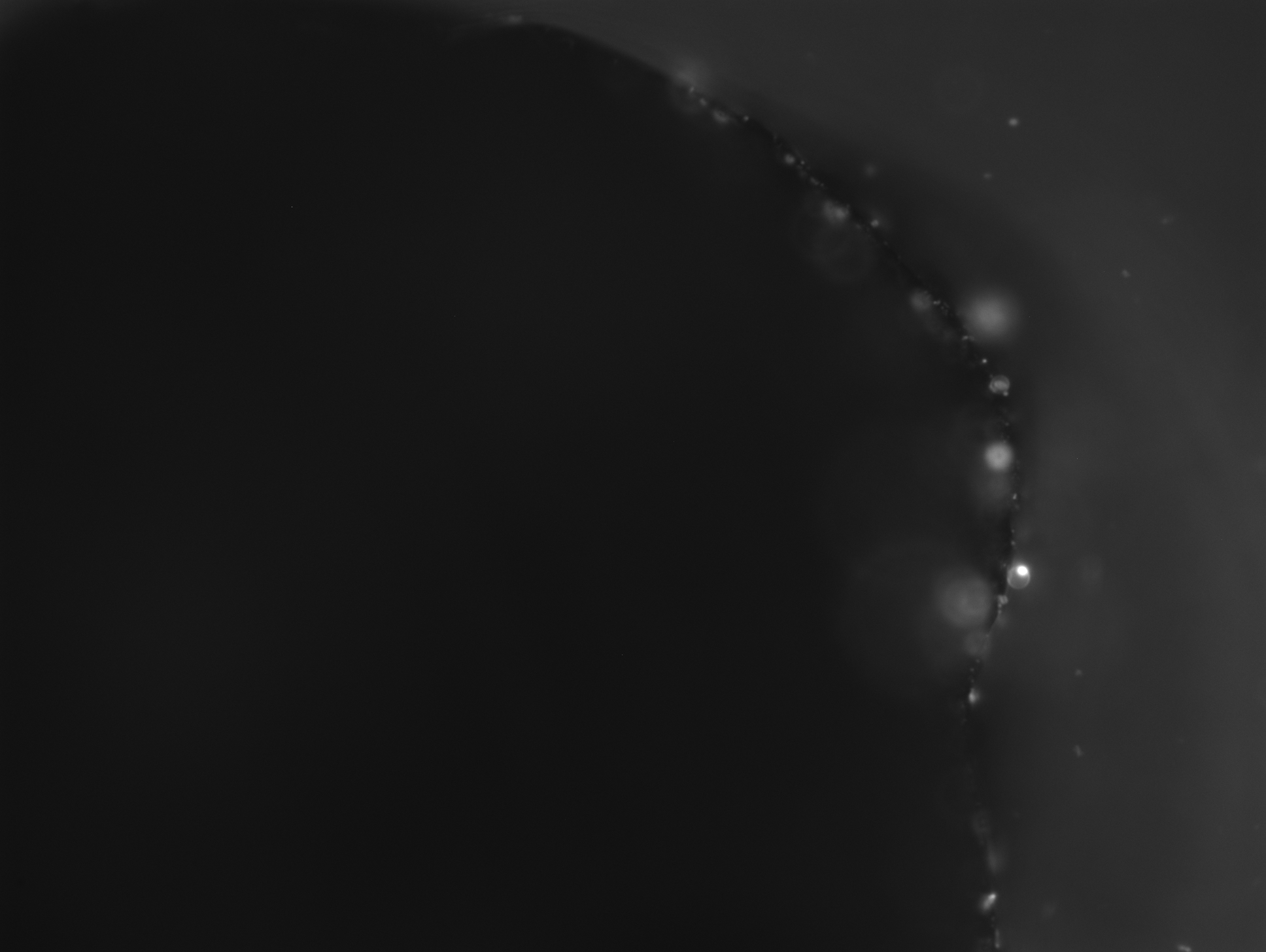

Supplement: Supplementary file 1 — Supplementary Material 1 [file 41598_2026_60022_MOESM1_ESM.zip › SI_Fig5_source images/A1_s1/3_23_21.050.tif]

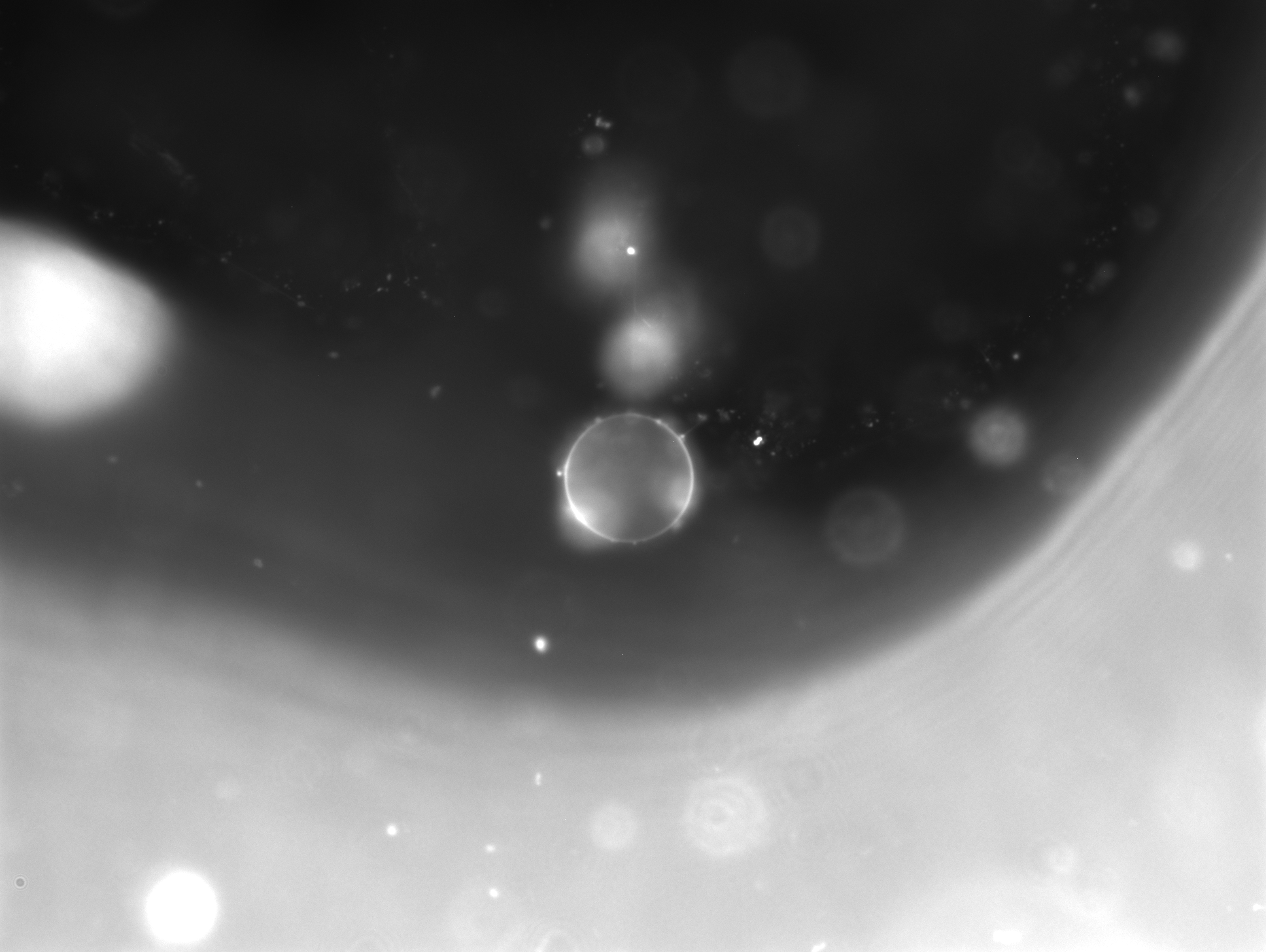

Supplement: Supplementary file 1 — Supplementary Material 1 [file 41598_2026_60022_MOESM1_ESM.zip › SI_Fig5_source images/A2_s2/1_37_52.531.tif]

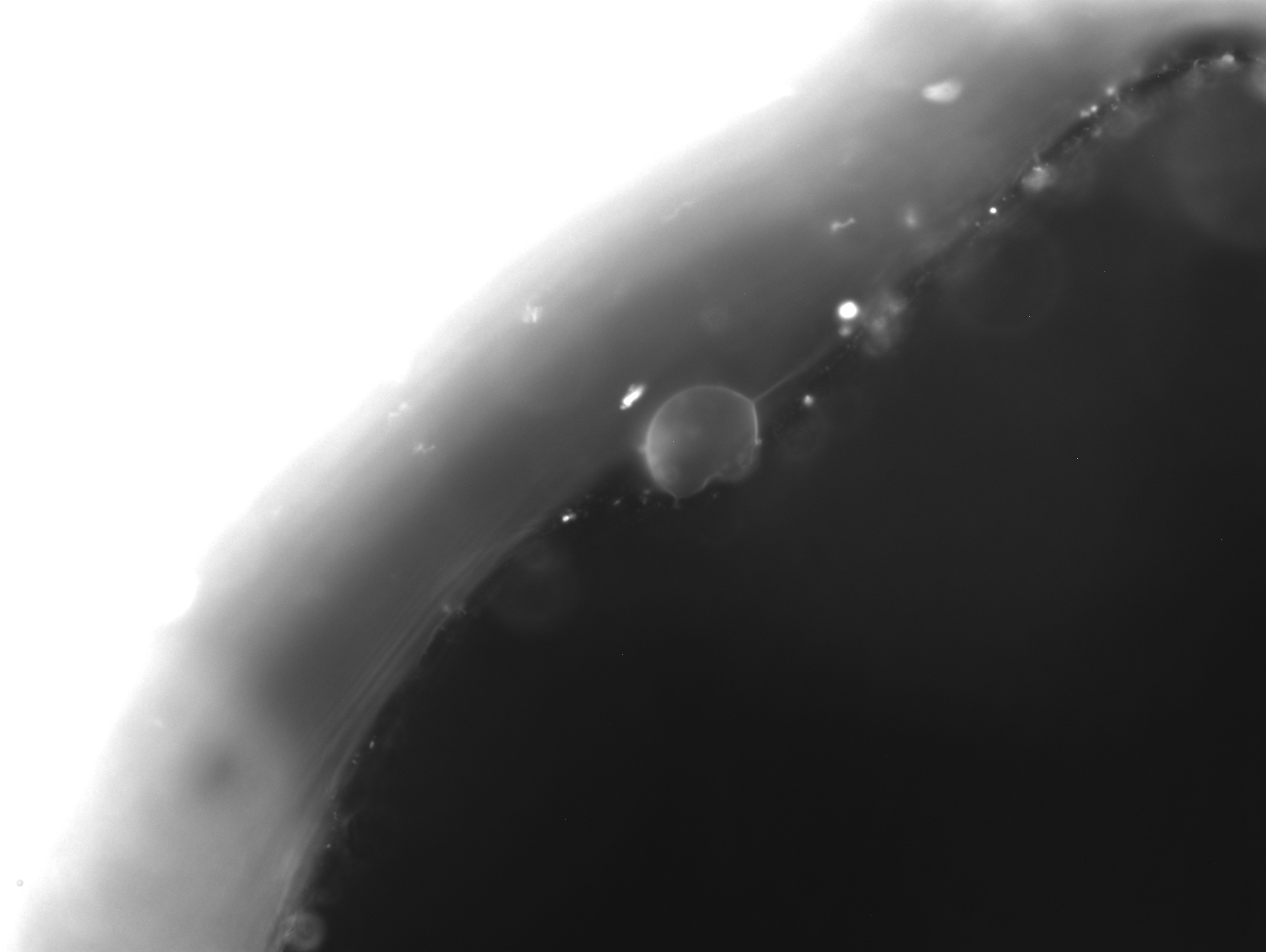

Supplement: Supplementary file 1 — Supplementary Material 1 [file 41598_2026_60022_MOESM1_ESM.zip › SI_Fig5_source images/A2_s2/2_7_46.292.tif]

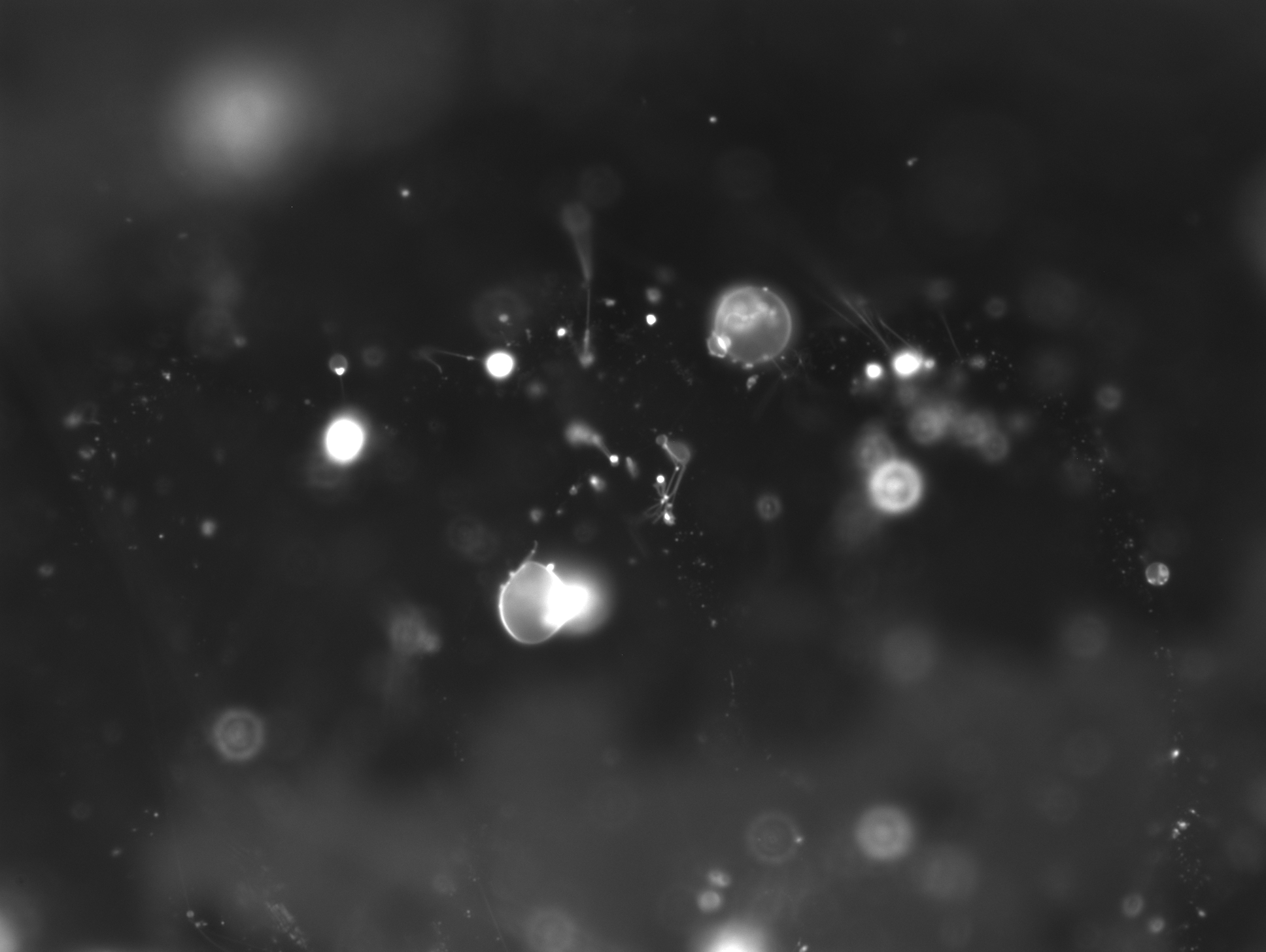

Supplement: Supplementary file 1 — Supplementary Material 1 [file 41598_2026_60022_MOESM1_ESM.zip › SI_Fig5_source images/A2_s2/3_65_18.857.tif]

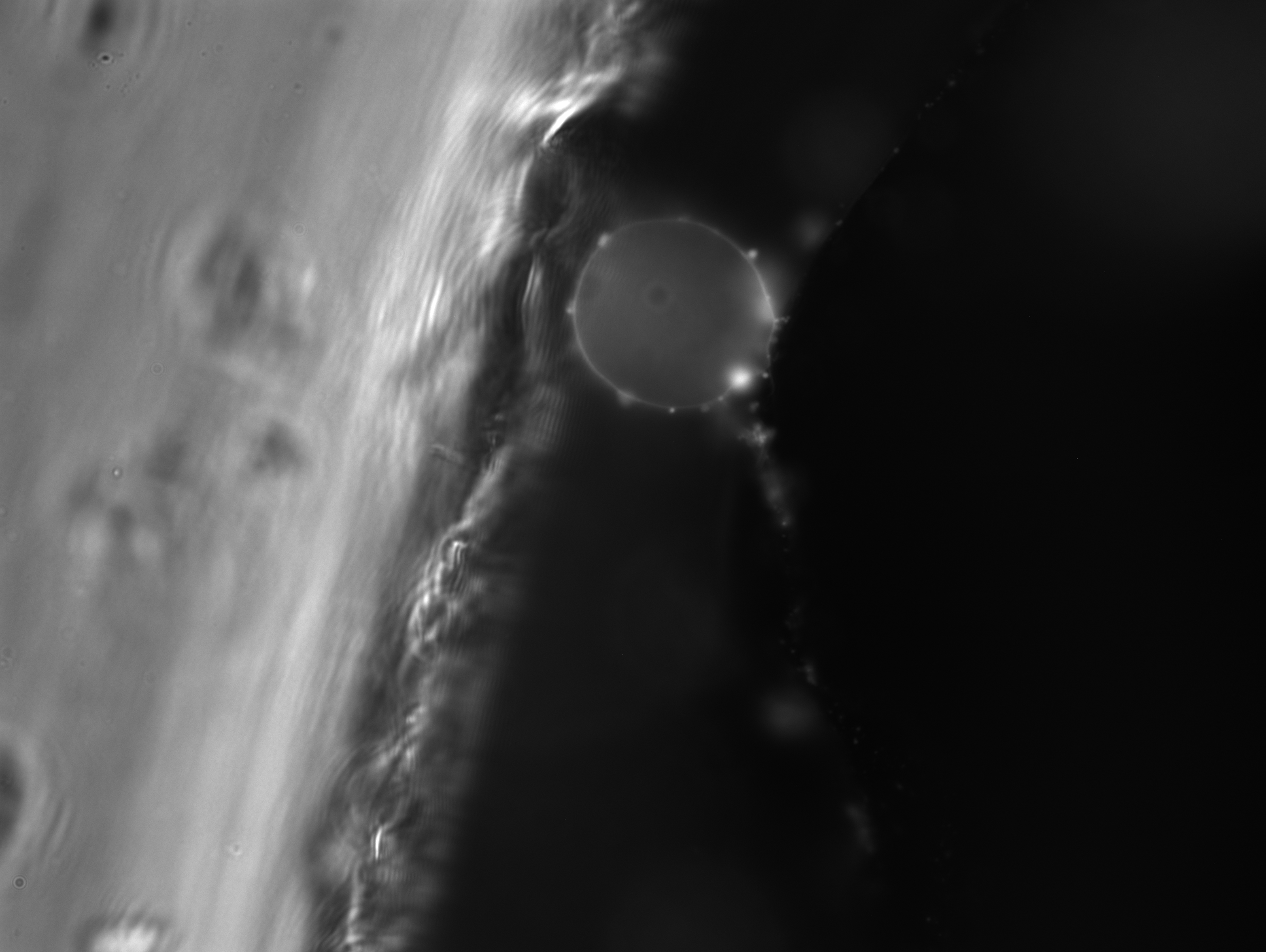

Supplement: Supplementary file 1 — Supplementary Material 1 [file 41598_2026_60022_MOESM1_ESM.zip › SI_Fig5_source images/A3_s3/1_88_16.009.tif]

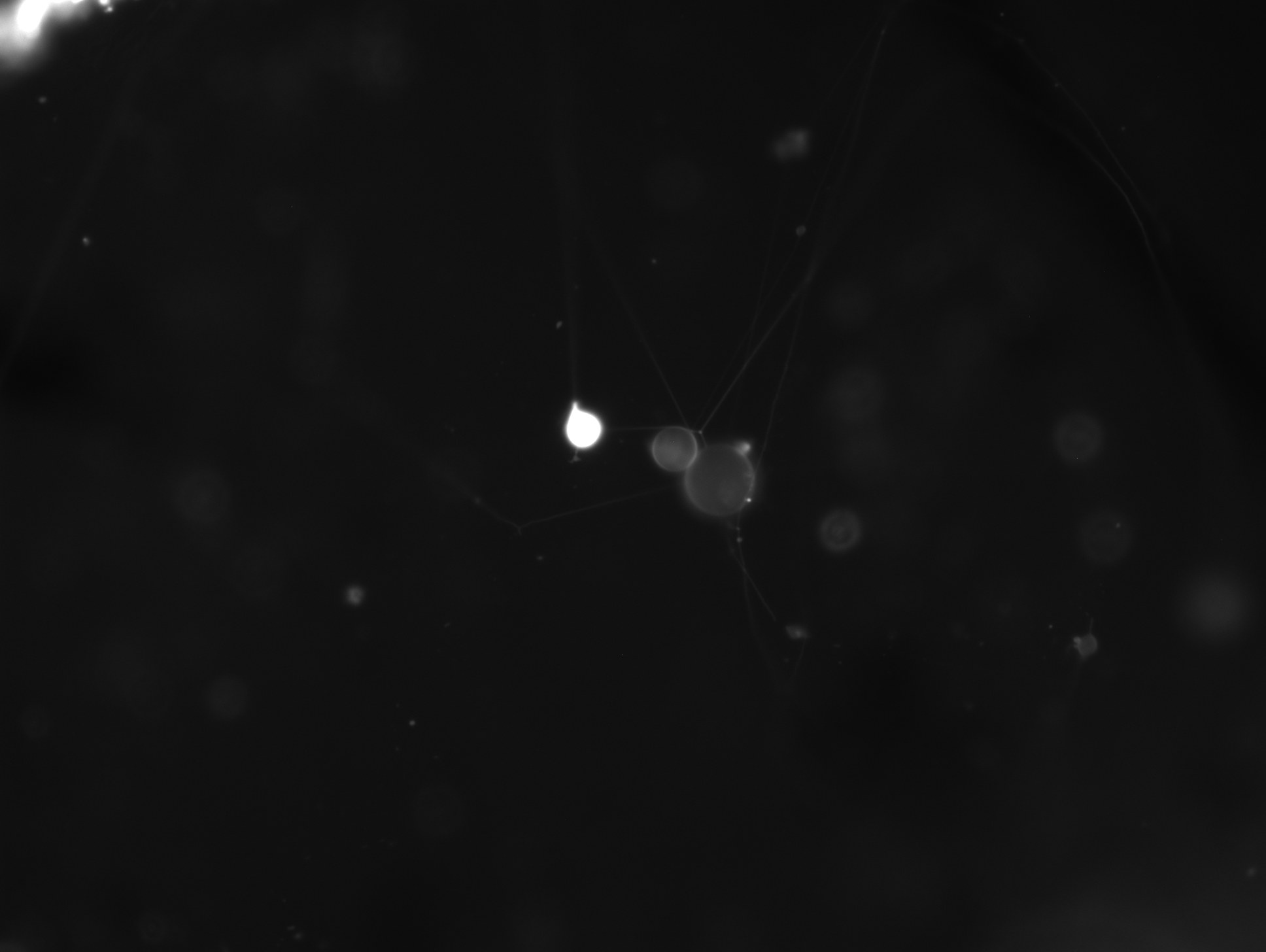

Supplement: Supplementary file 1 — Supplementary Material 1 [file 41598_2026_60022_MOESM1_ESM.zip › SI_Fig5_source images/A3_s3/2_31_26.363.tif]

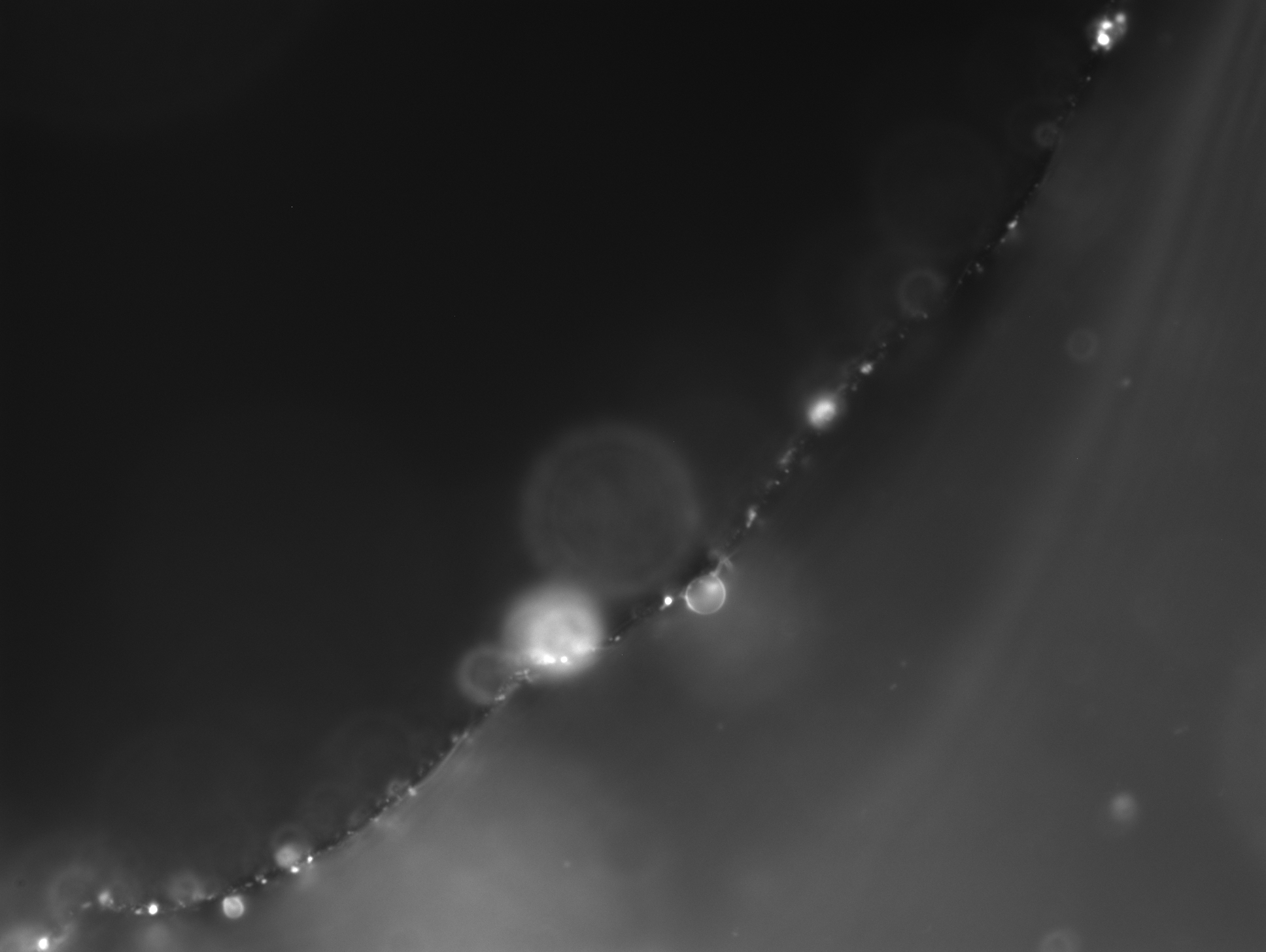

Supplement: Supplementary file 1 — Supplementary Material 1 [file 41598_2026_60022_MOESM1_ESM.zip › SI_Fig5_source images/A3_s3/3_109_05.979.tif]

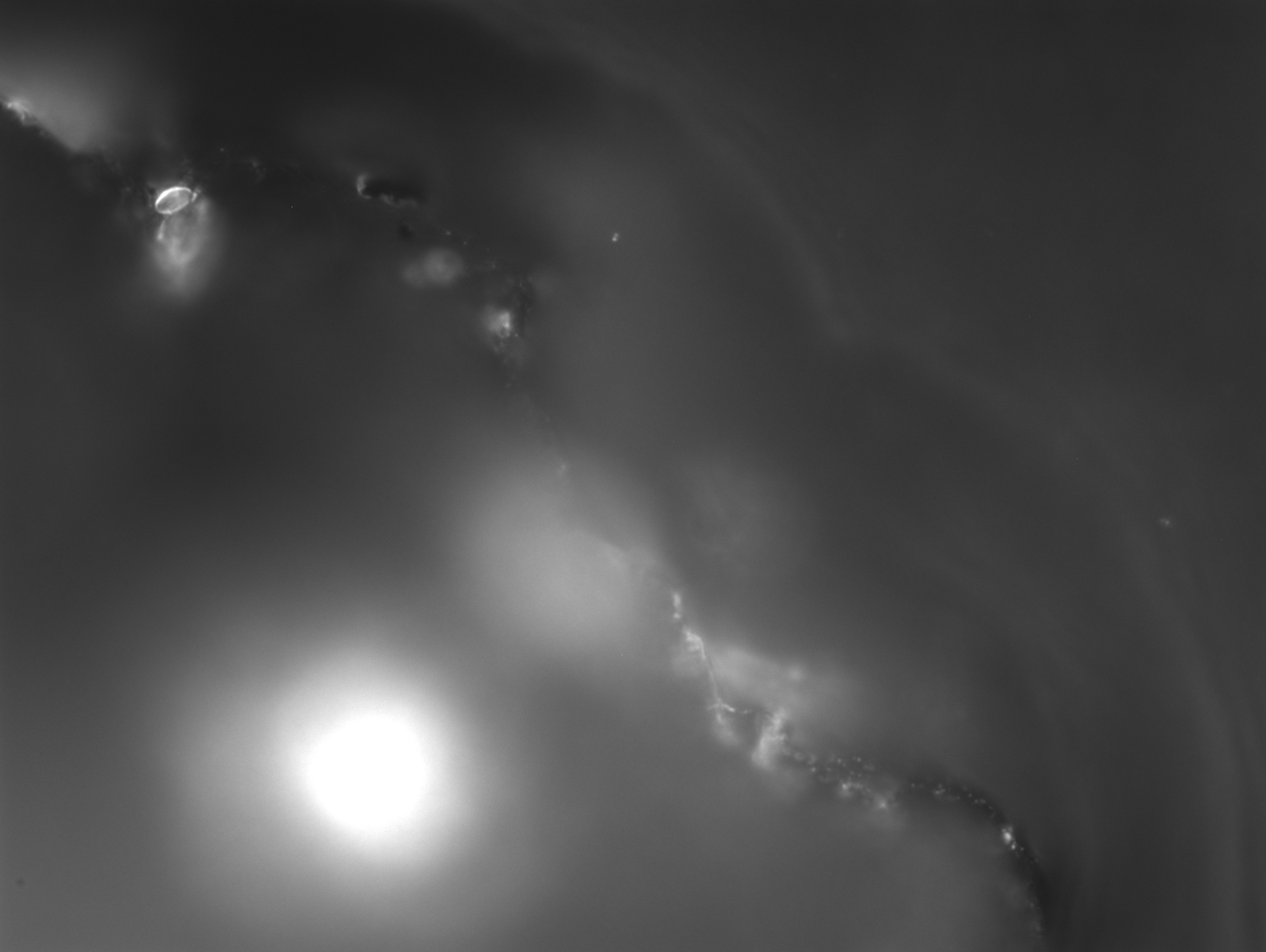

Supplement: Supplementary file 1 — Supplementary Material 1 [file 41598_2026_60022_MOESM1_ESM.zip › SI_Fig5_source images/A4_p1/1_57_21.553.tif]

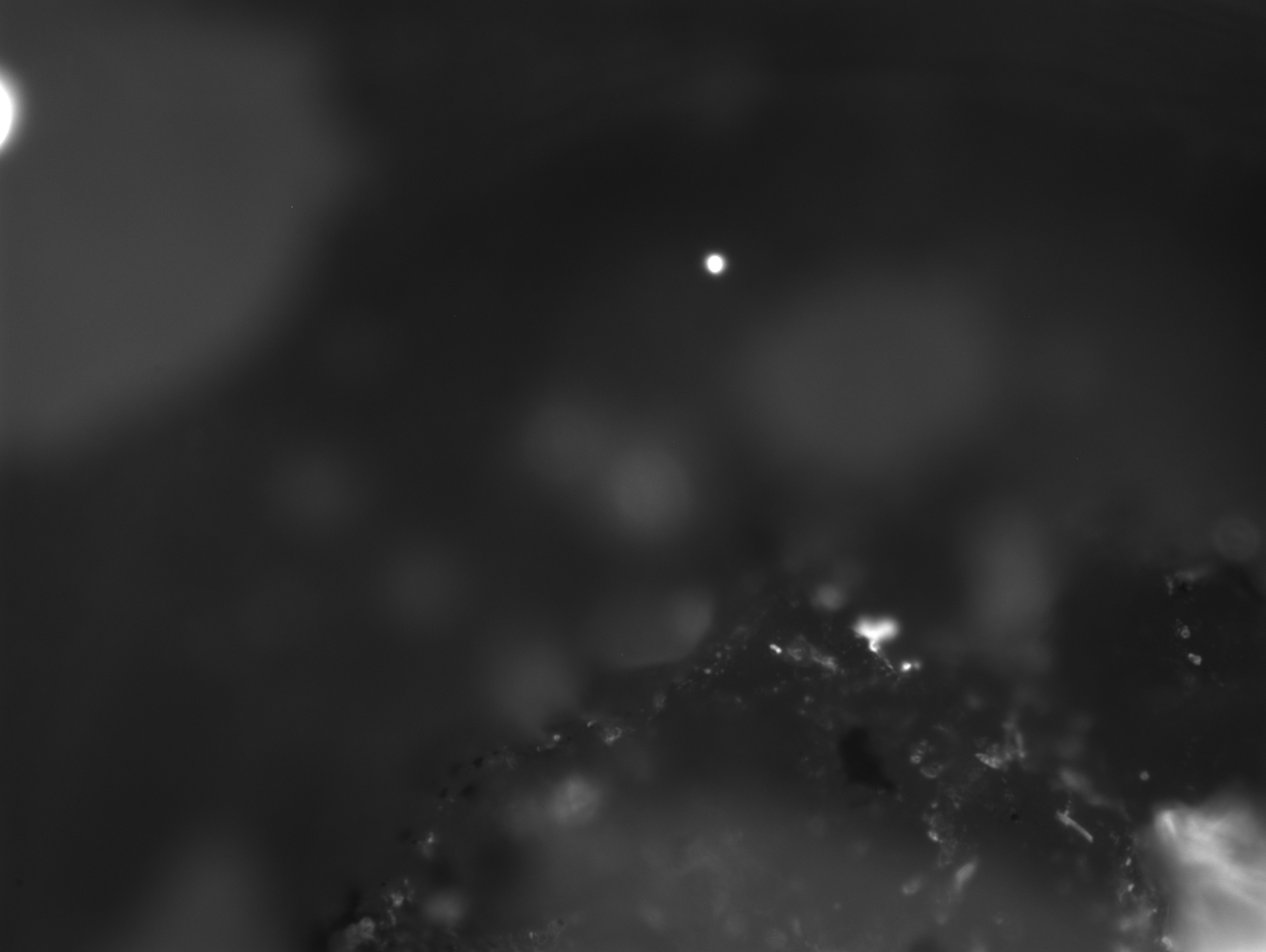

Supplement: Supplementary file 1 — Supplementary Material 1 [file 41598_2026_60022_MOESM1_ESM.zip › SI_Fig5_source images/A4_p1/2_36_27.123.tif]

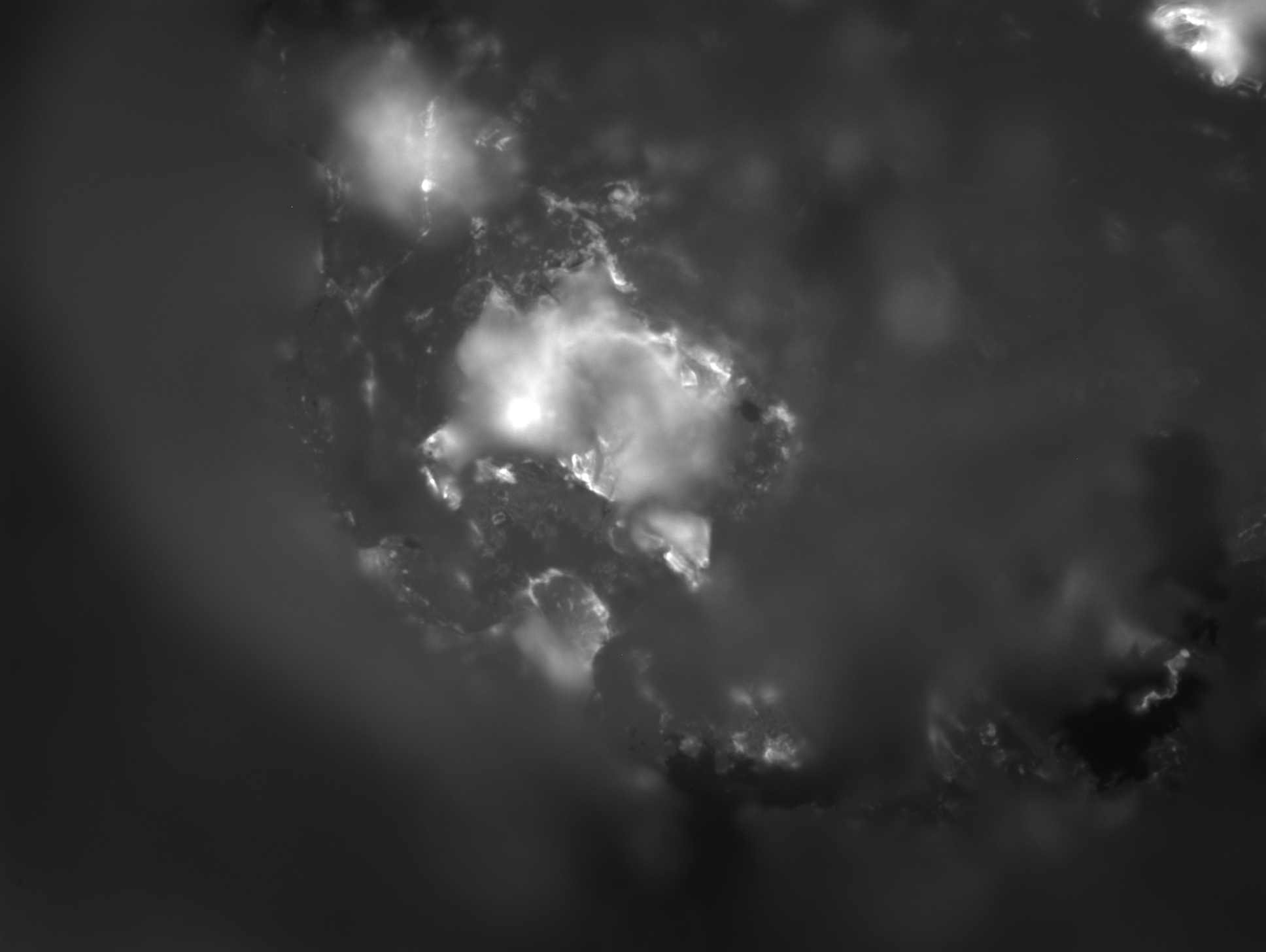

Supplement: Supplementary file 1 — Supplementary Material 1 [file 41598_2026_60022_MOESM1_ESM.zip › SI_Fig5_source images/A4_p1/3_36_46.675.tif]

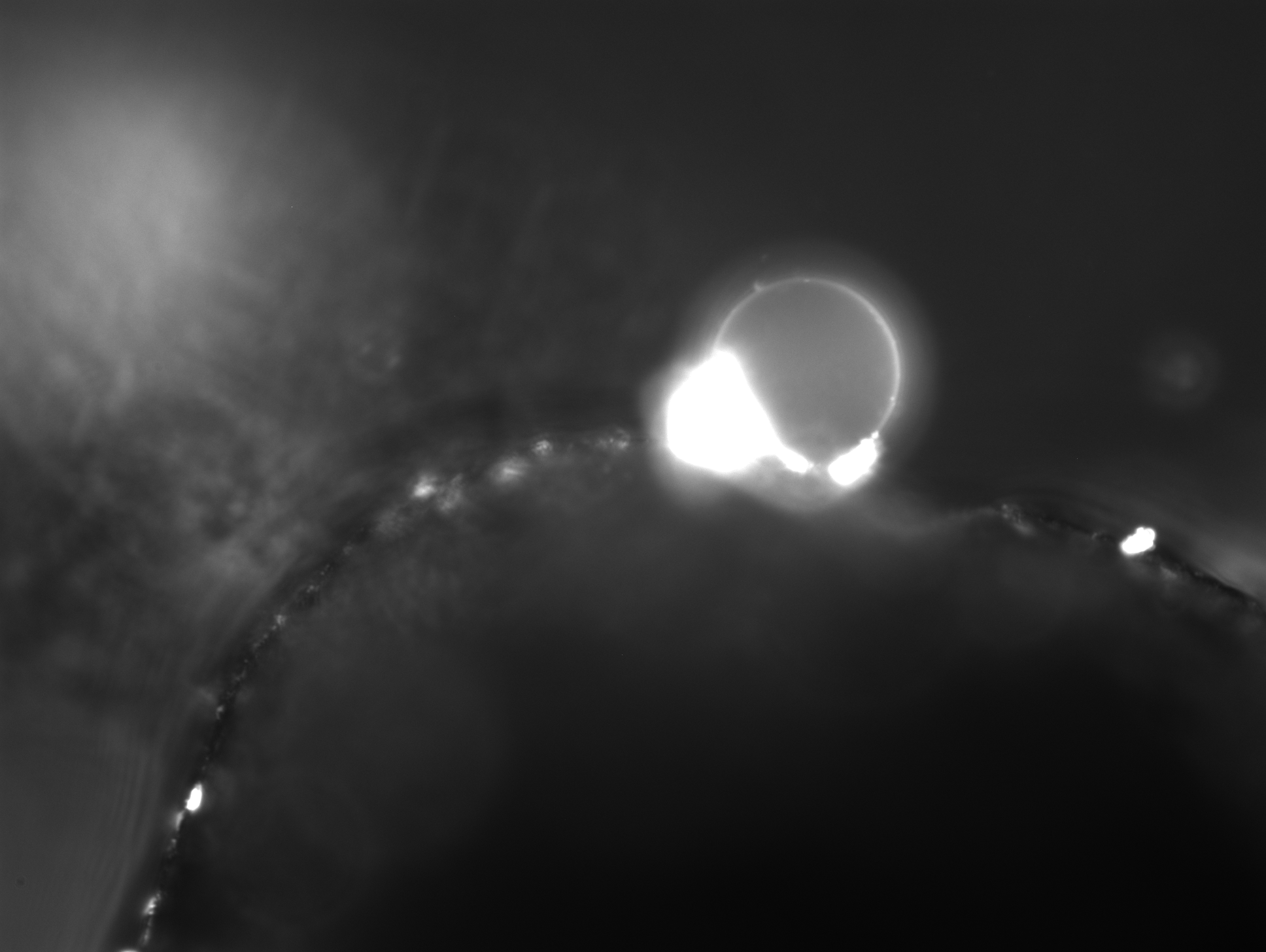

Supplement: Supplementary file 1 — Supplementary Material 1 [file 41598_2026_60022_MOESM1_ESM.zip › SI_Fig5_source images/A5_p2/1_1_58.986.tif]

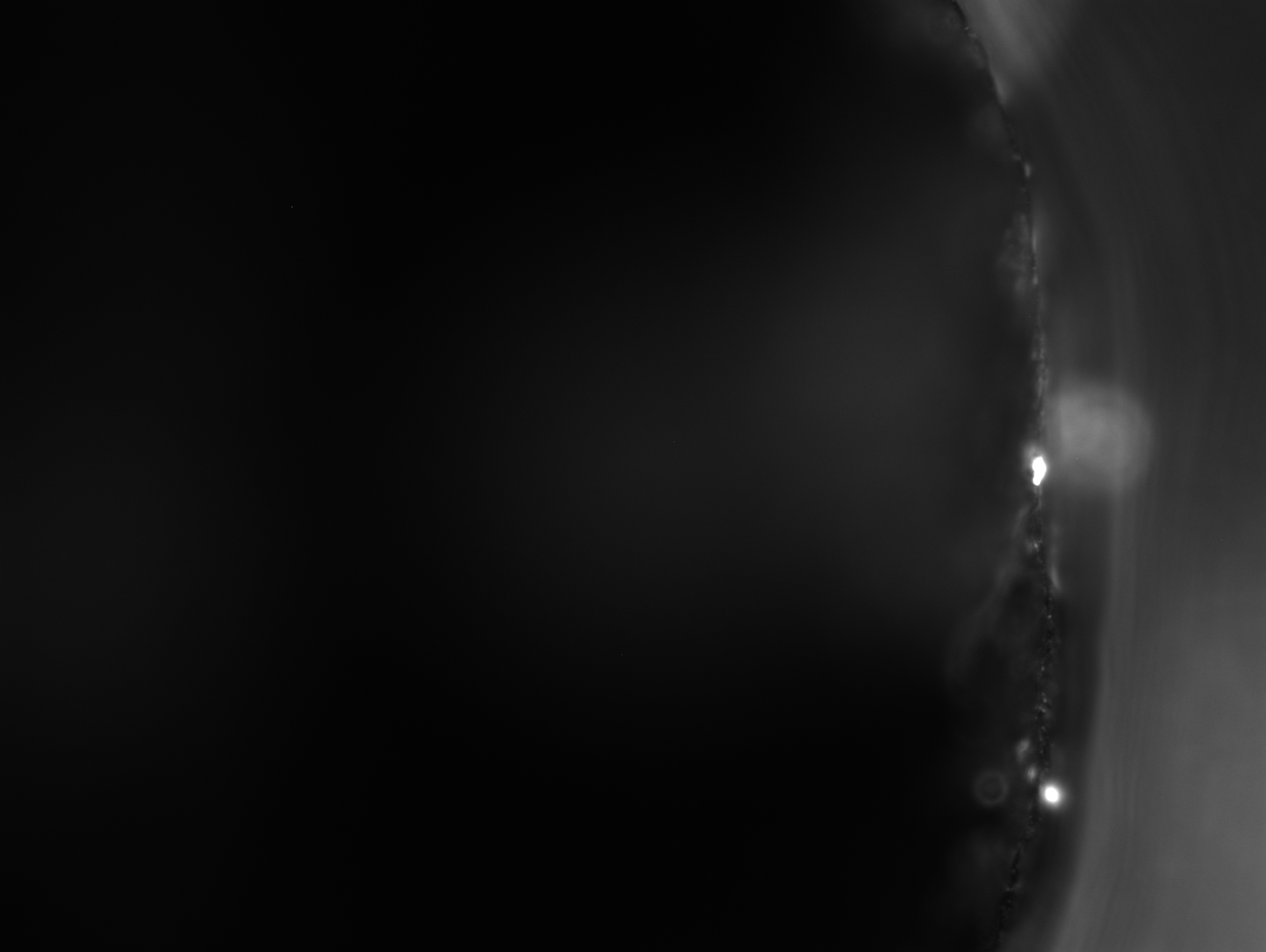

Supplement: Supplementary file 1 — Supplementary Material 1 [file 41598_2026_60022_MOESM1_ESM.zip › SI_Fig5_source images/A5_p2/2_81_48.363.tif]

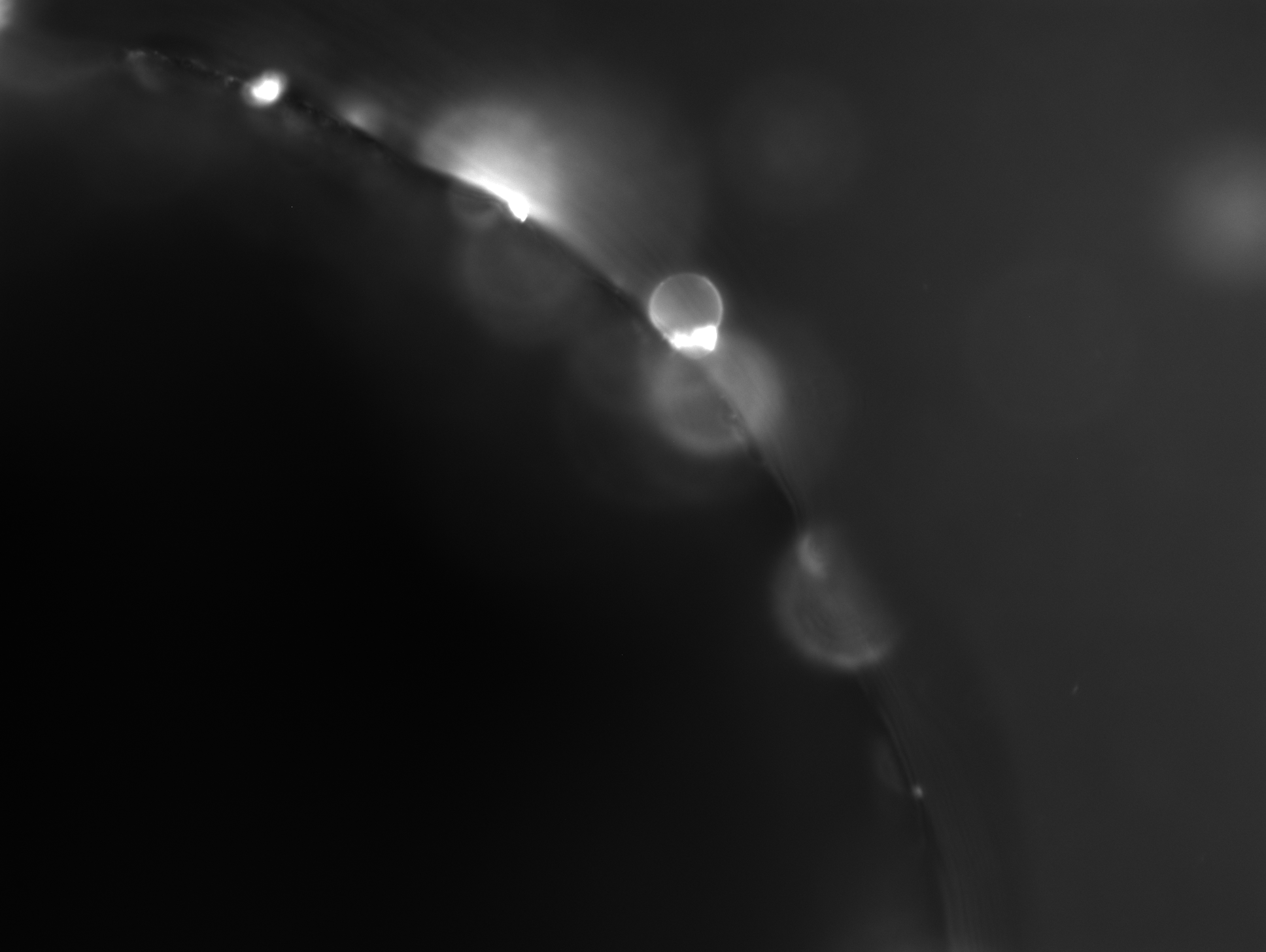

Supplement: Supplementary file 1 — Supplementary Material 1 [file 41598_2026_60022_MOESM1_ESM.zip › SI_Fig5_source images/A5_p2/3_69_21.530.tif]

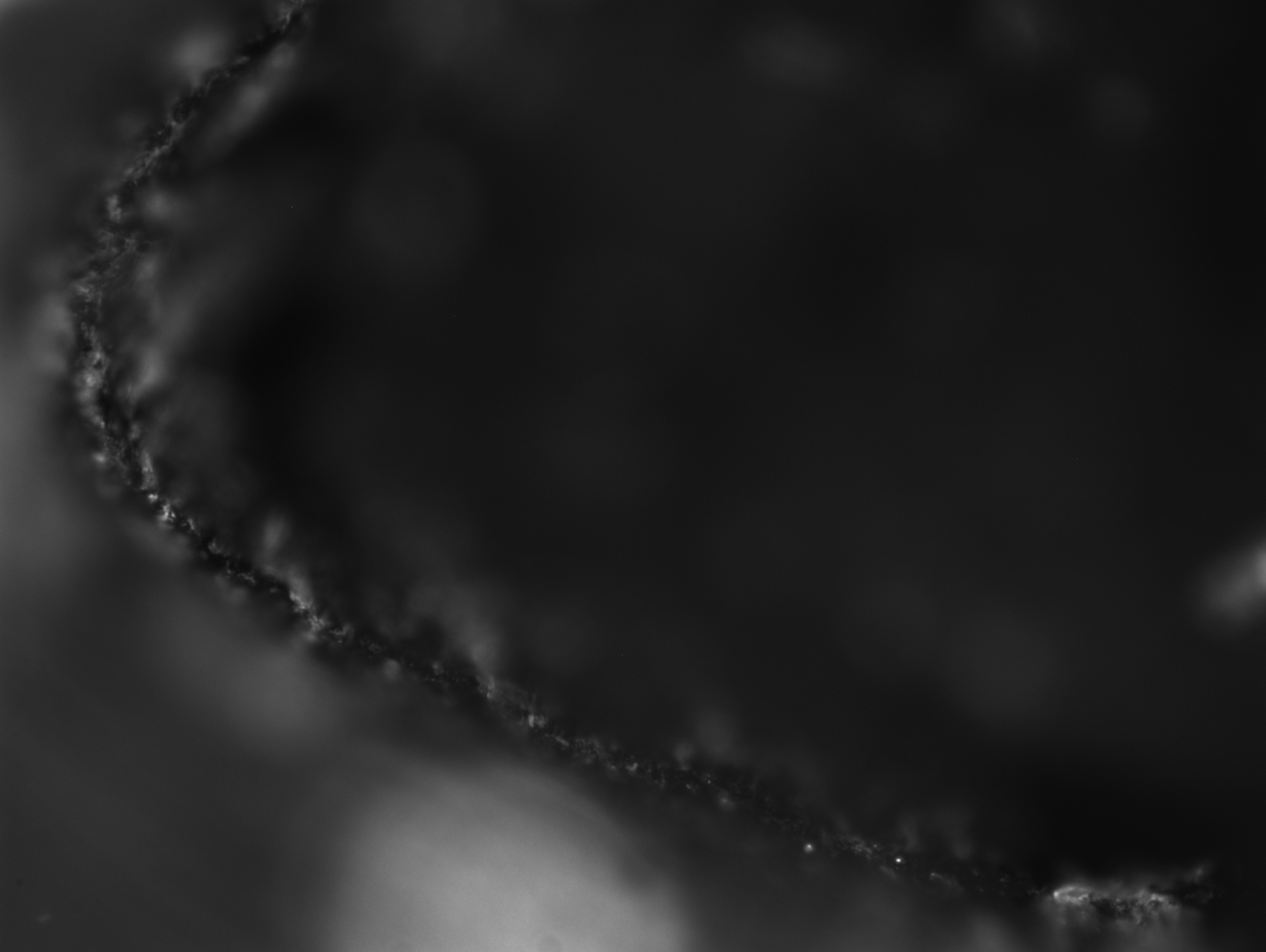

Supplement: Supplementary file 1 — Supplementary Material 1 [file 41598_2026_60022_MOESM1_ESM.zip › SI_Fig5_source images/A6_p3/1_58_56.395.tif]

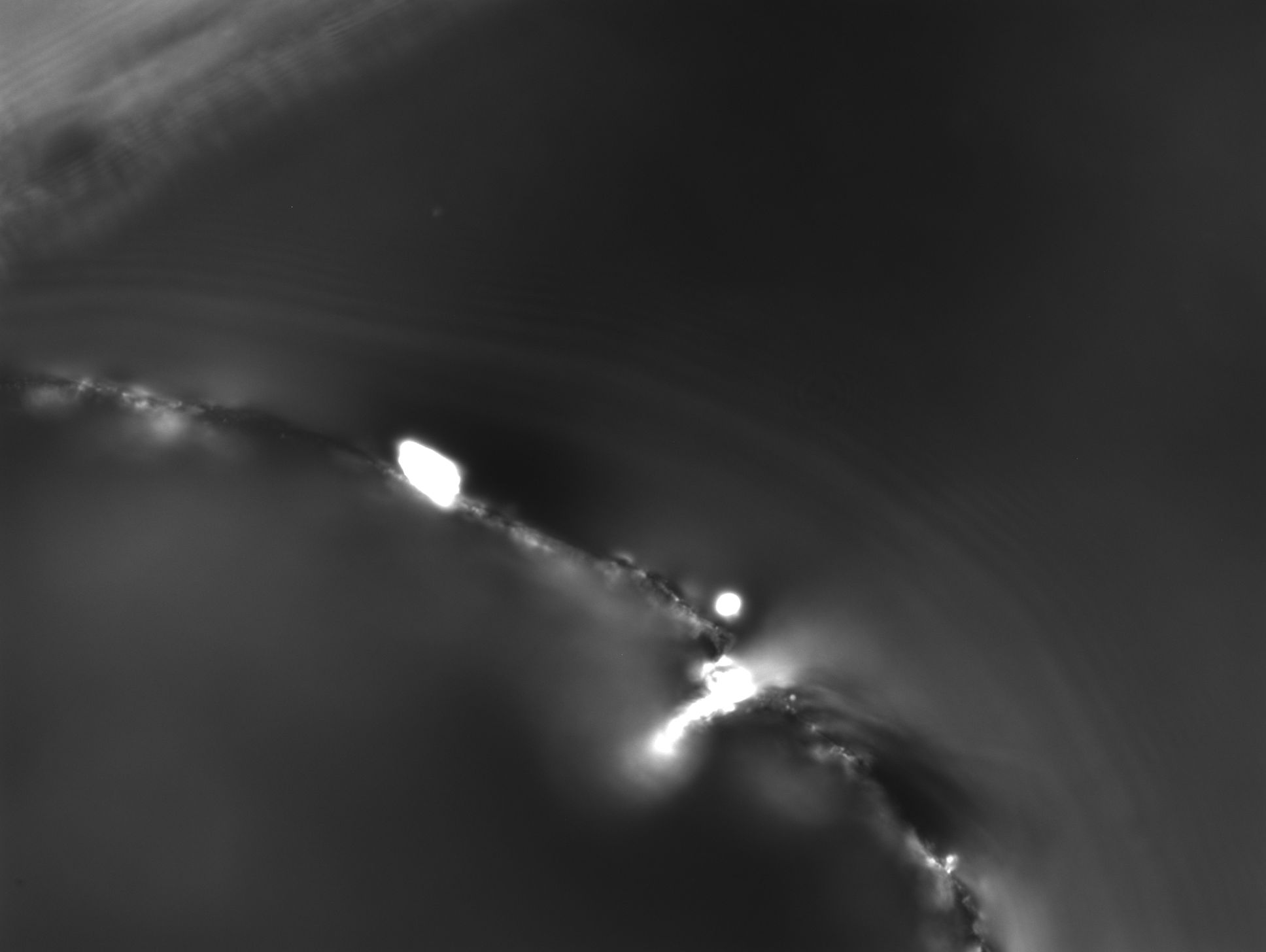

Supplement: Supplementary file 1 — Supplementary Material 1 [file 41598_2026_60022_MOESM1_ESM.zip › SI_Fig5_source images/A6_p3/2_72_06.402.tif]

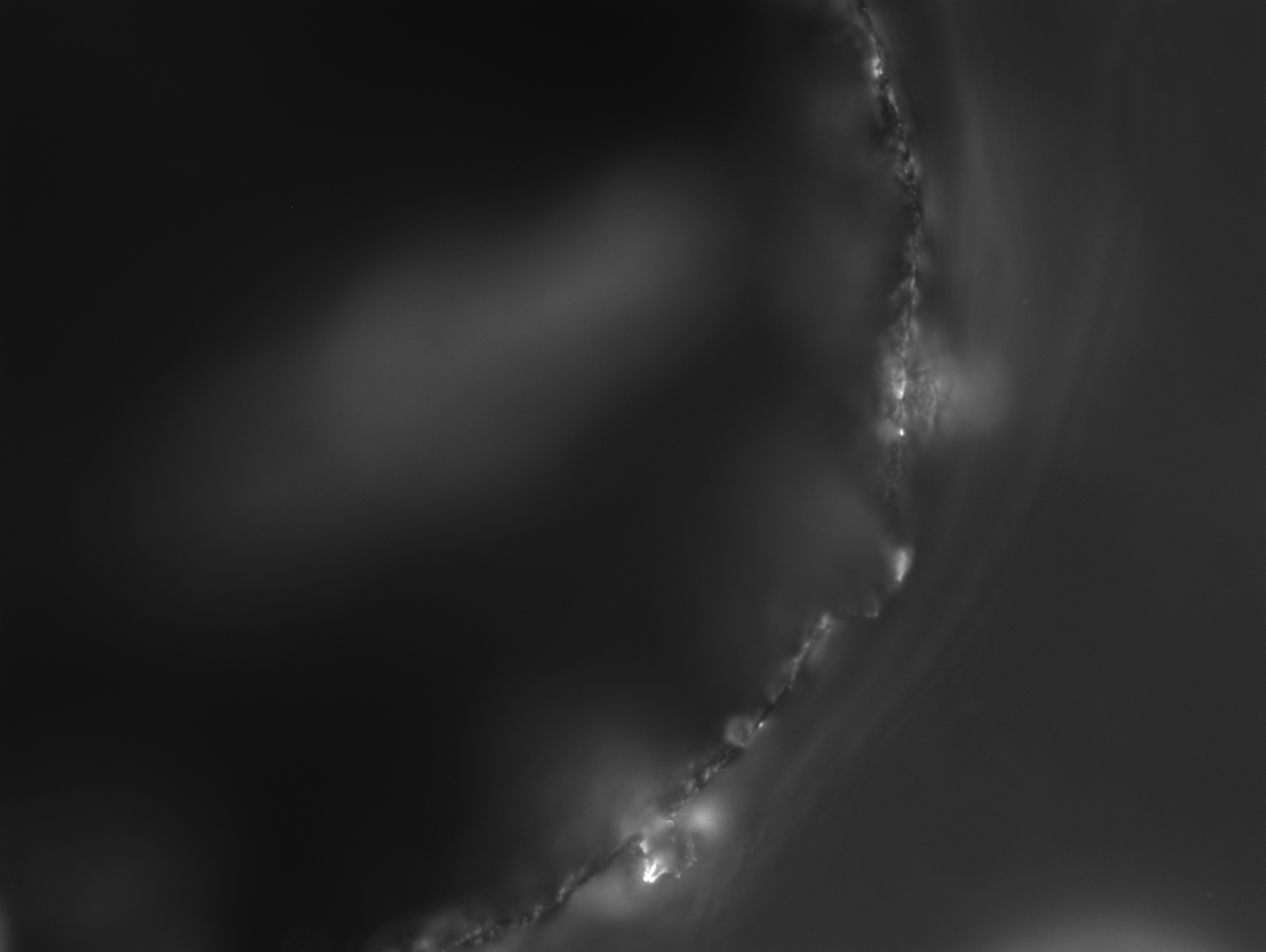

Supplement: Supplementary file 1 — Supplementary Material 1 [file 41598_2026_60022_MOESM1_ESM.zip › SI_Fig5_source images/A6_p3/3_107_32.506.tif]

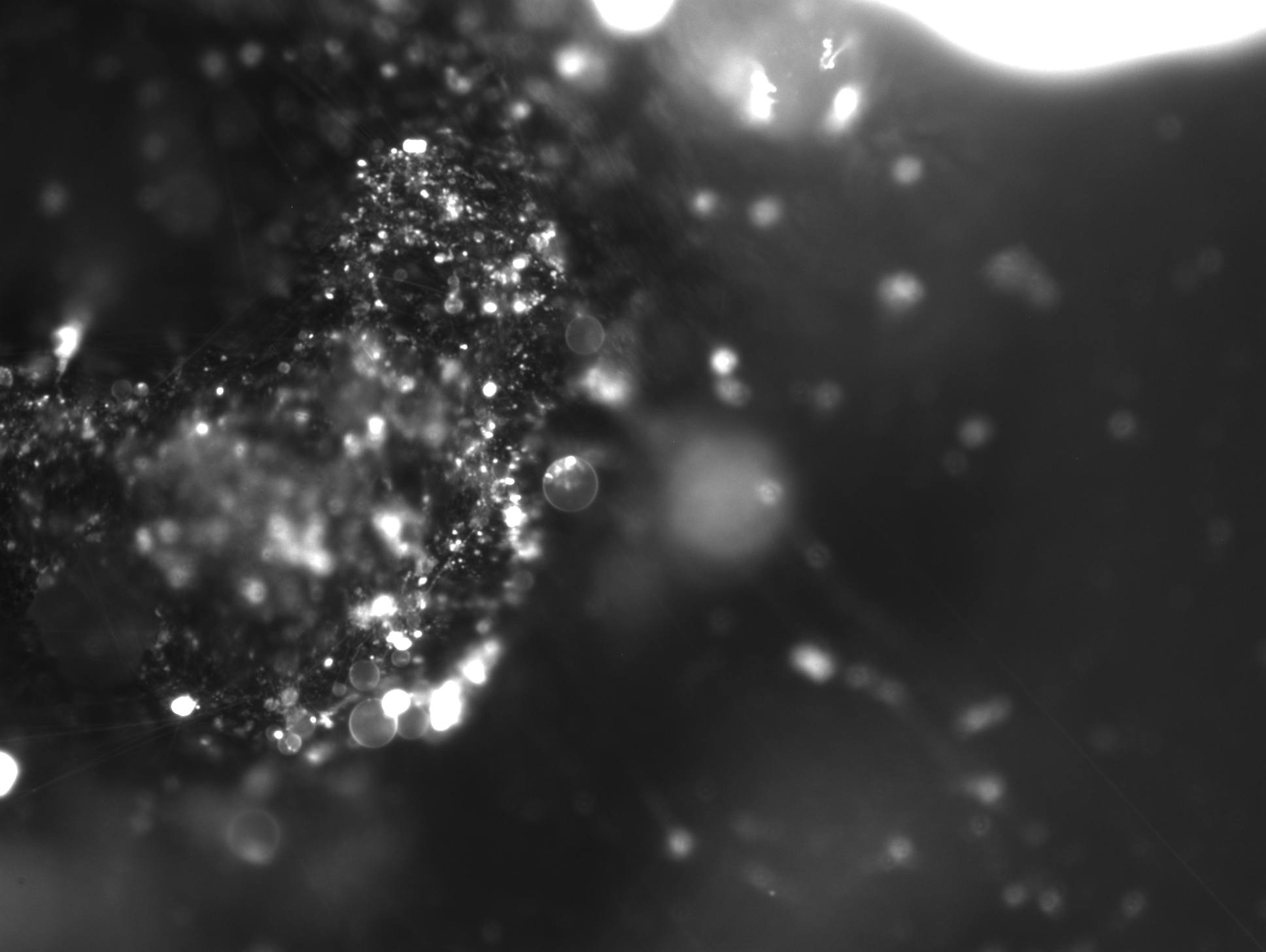

Supplement: Supplementary file 1 — Supplementary Material 1 [file 41598_2026_60022_MOESM1_ESM.zip › SI_Fig5_source images/A7_m1/1_20_42.053.tif]

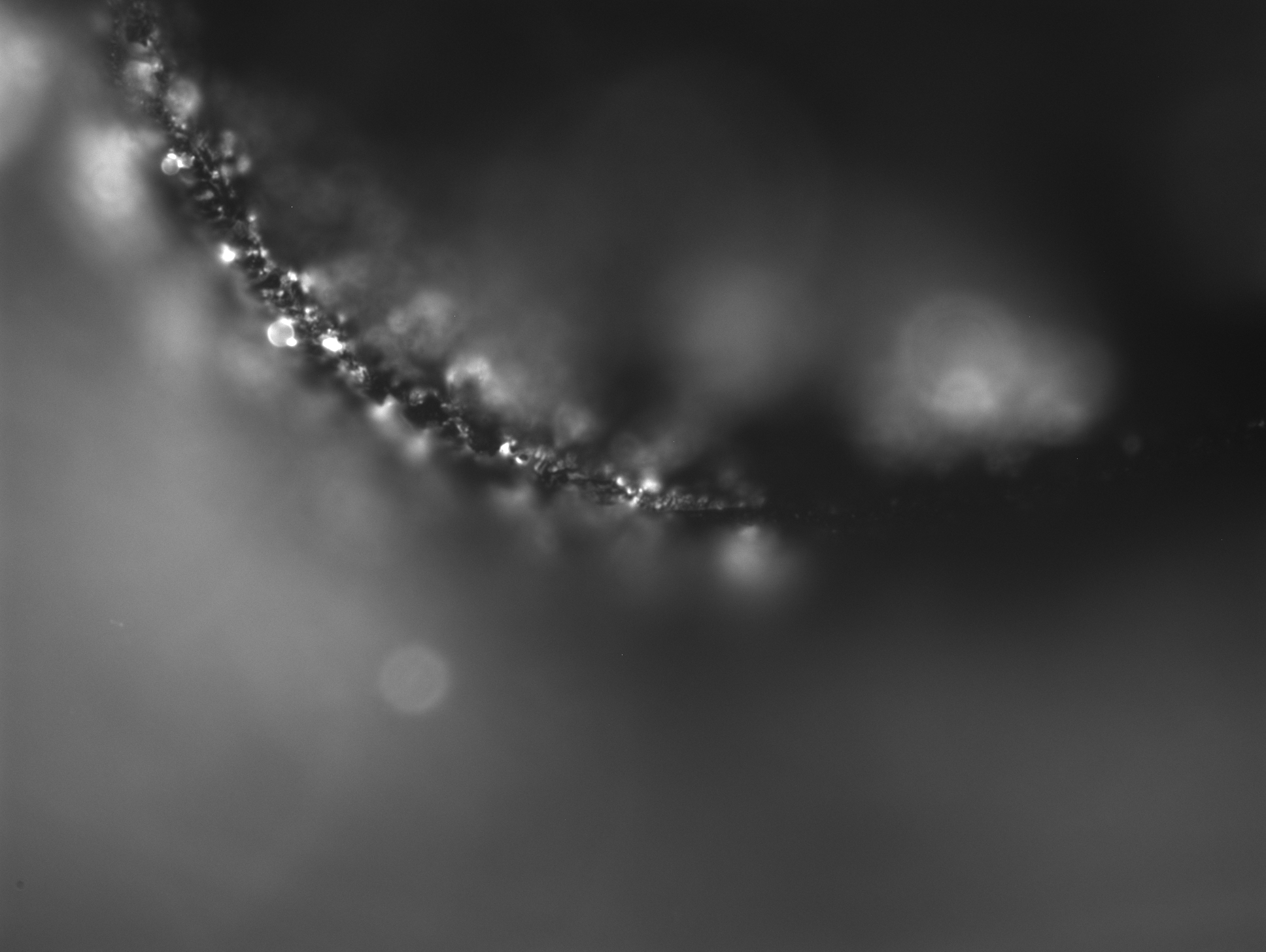

Supplement: Supplementary file 1 — Supplementary Material 1 [file 41598_2026_60022_MOESM1_ESM.zip › SI_Fig5_source images/A7_m1/2_74_54.700.tif]

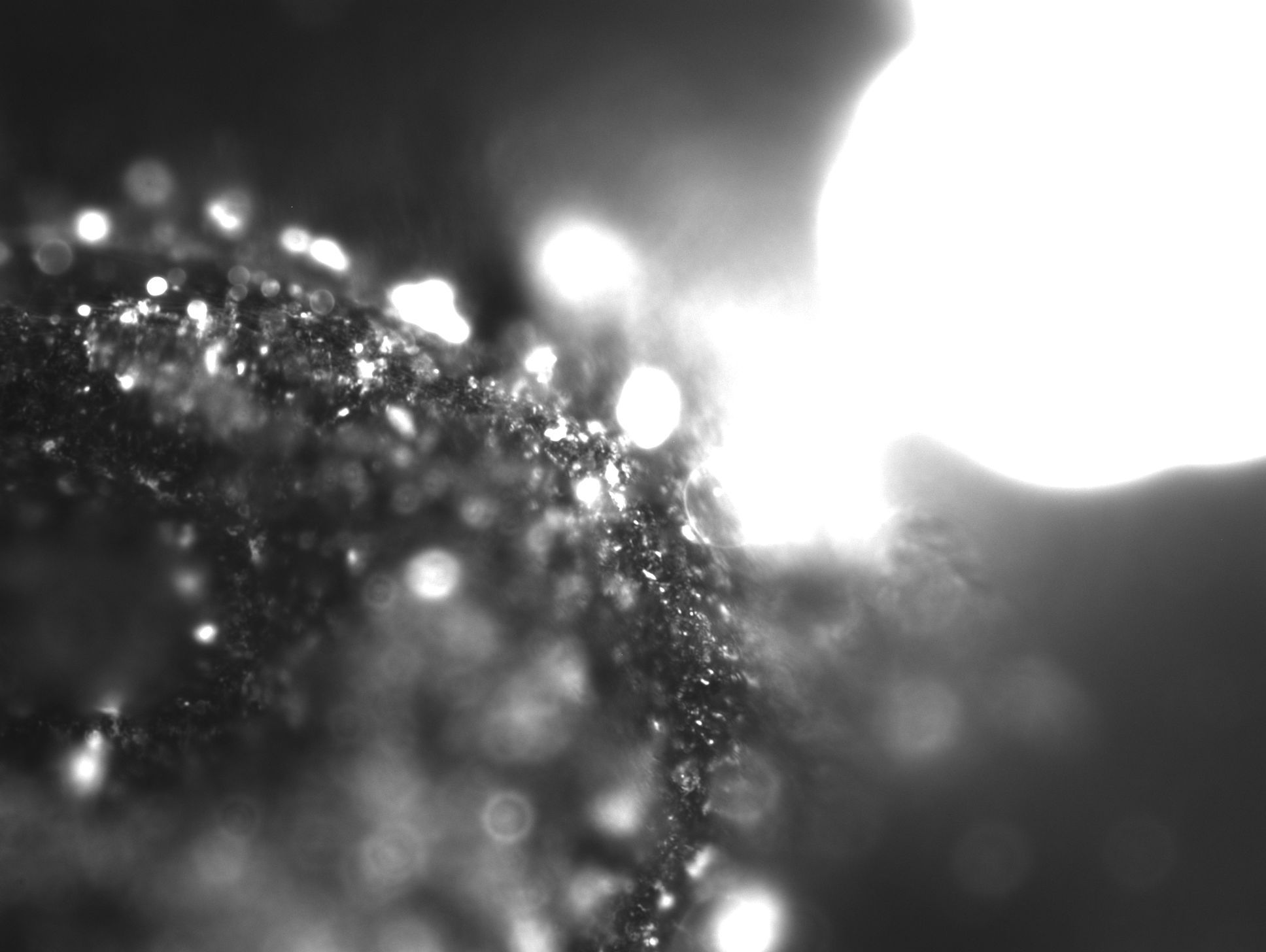

Supplement: Supplementary file 1 — Supplementary Material 1 [file 41598_2026_60022_MOESM1_ESM.zip › SI_Fig5_source images/A7_m1/3_12.094.tif]

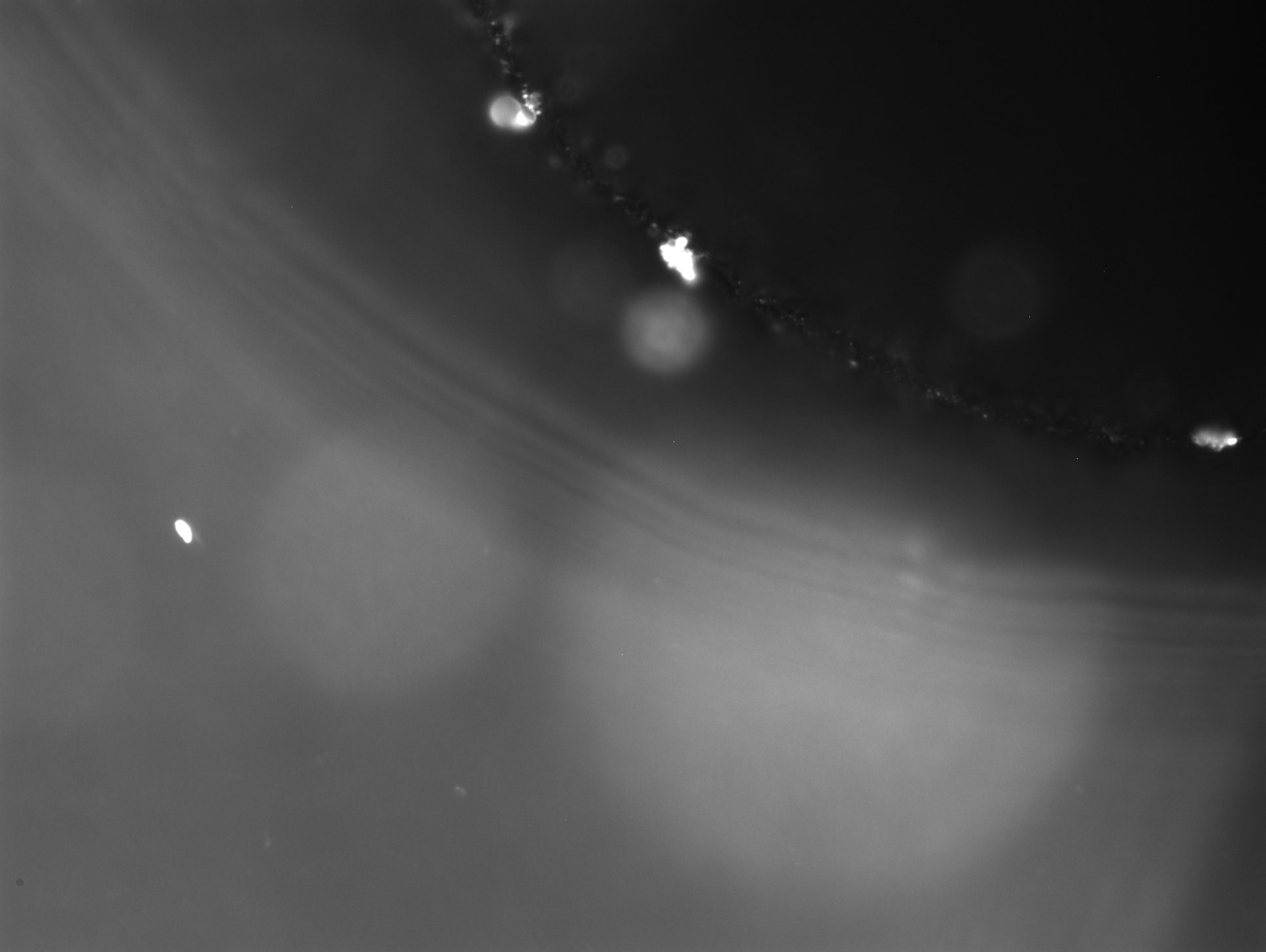

Supplement: Supplementary file 1 — Supplementary Material 1 [file 41598_2026_60022_MOESM1_ESM.zip › SI_Fig5_source images/A8_m2/1_44.502-1.tif]

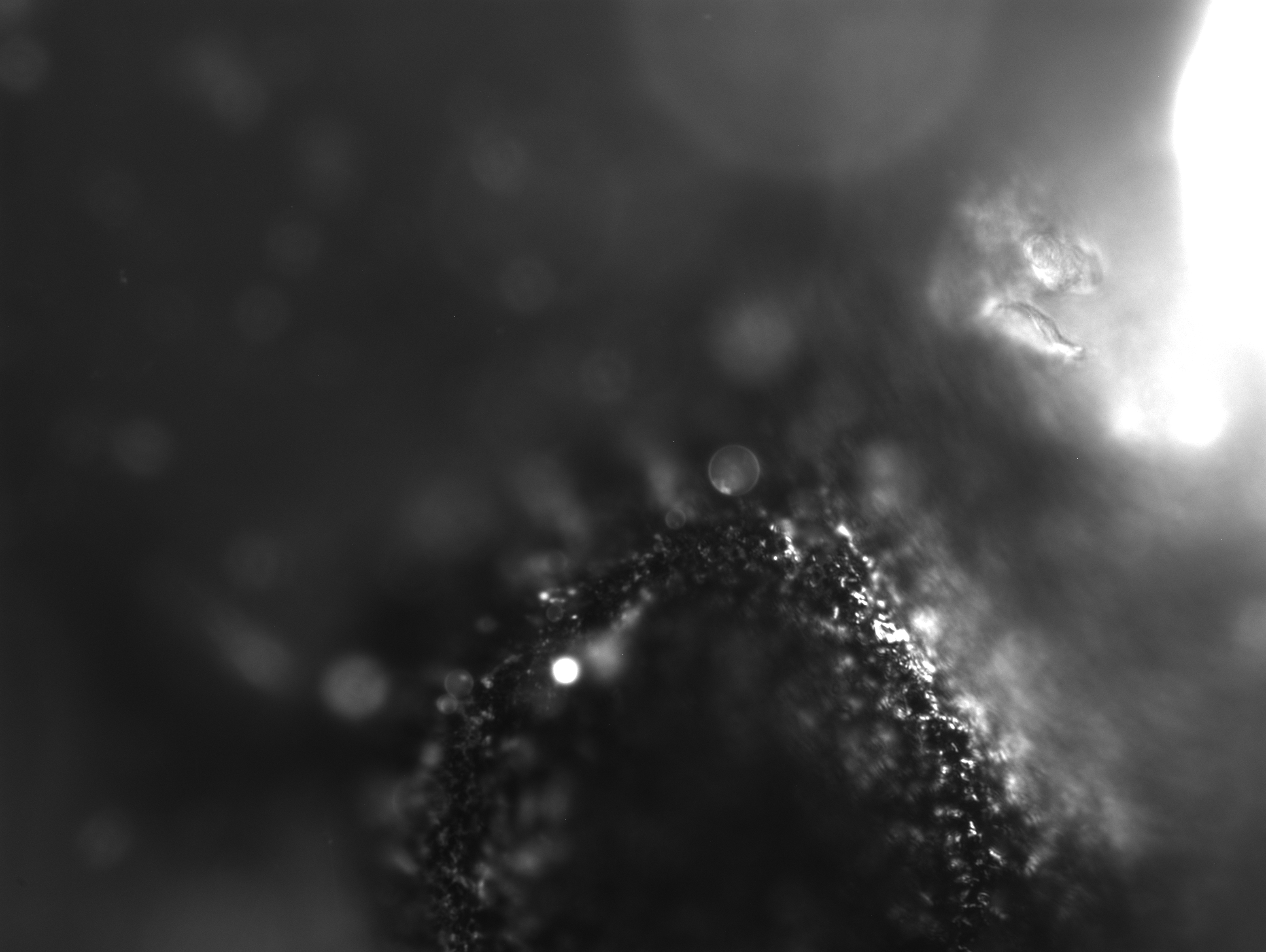

Supplement: Supplementary file 1 — Supplementary Material 1 [file 41598_2026_60022_MOESM1_ESM.zip › SI_Fig5_source images/A8_m2/2_52.230-1.tif]

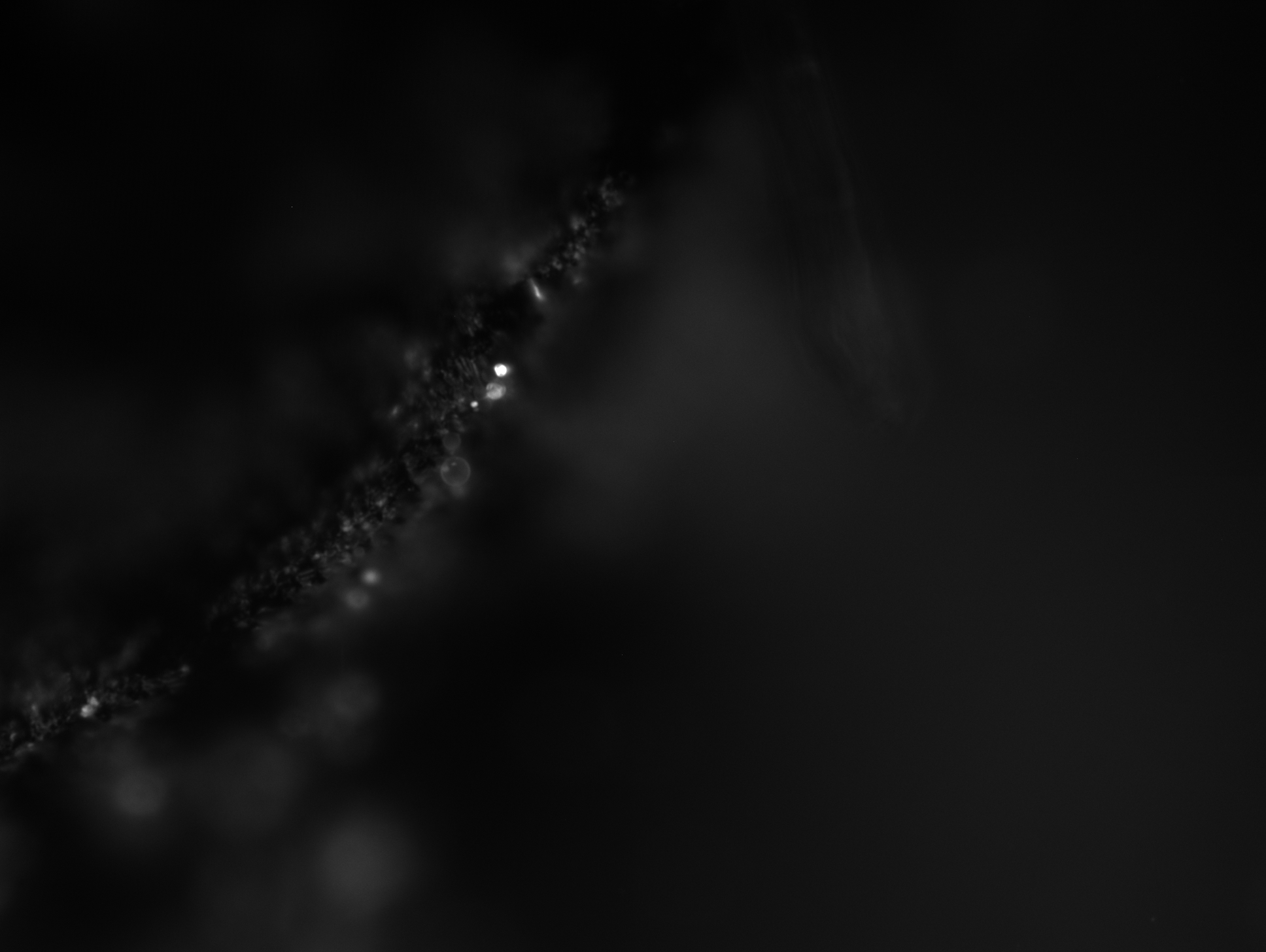

Supplement: Supplementary file 1 — Supplementary Material 1 [file 41598_2026_60022_MOESM1_ESM.zip › SI_Fig5_source images/A8_m2/3_22.838-1.tif]

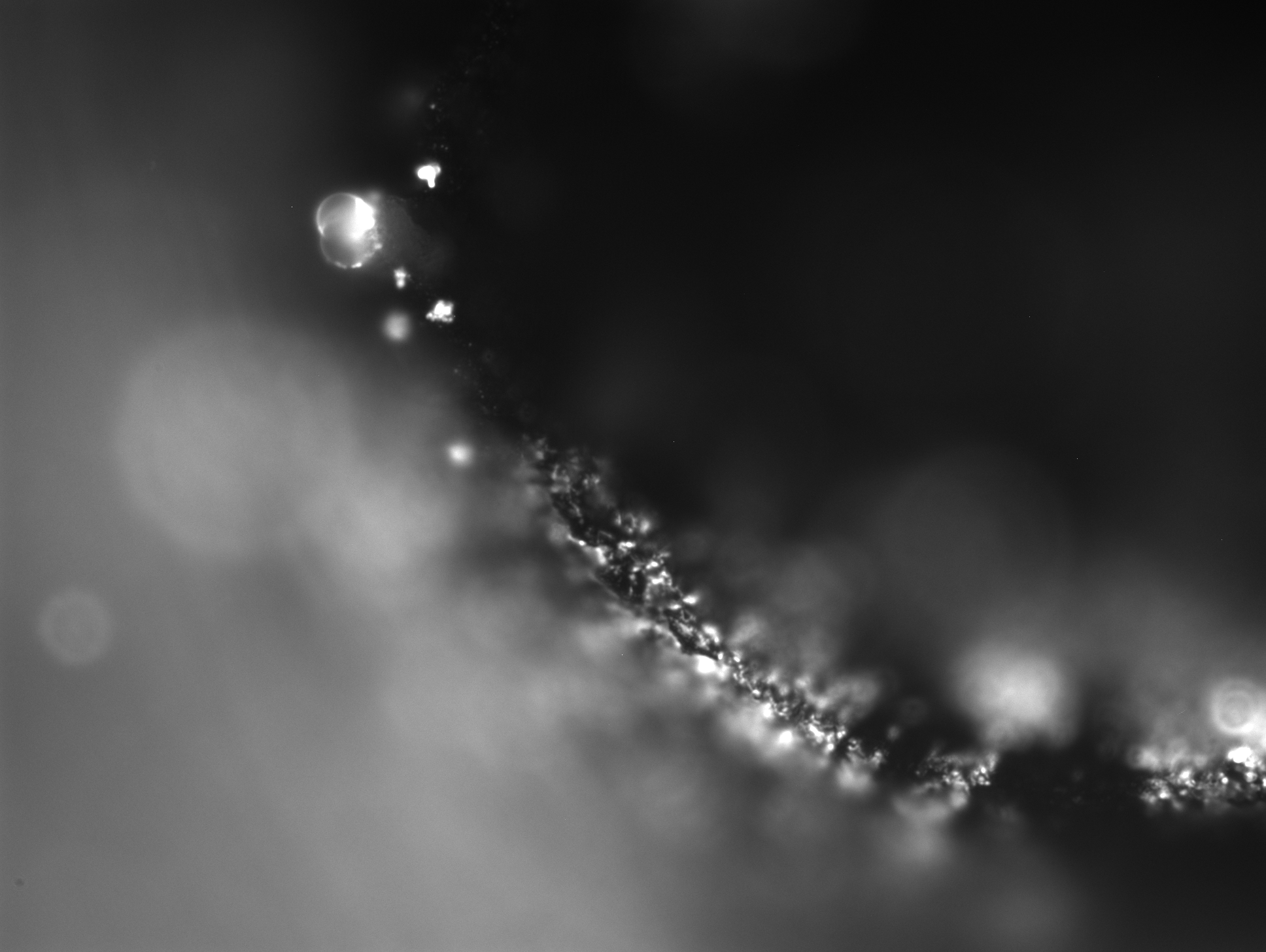

Supplement: Supplementary file 1 — Supplementary Material 1 [file 41598_2026_60022_MOESM1_ESM.zip › SI_Fig5_source images/A9_m3/1_40.581-1.tif]

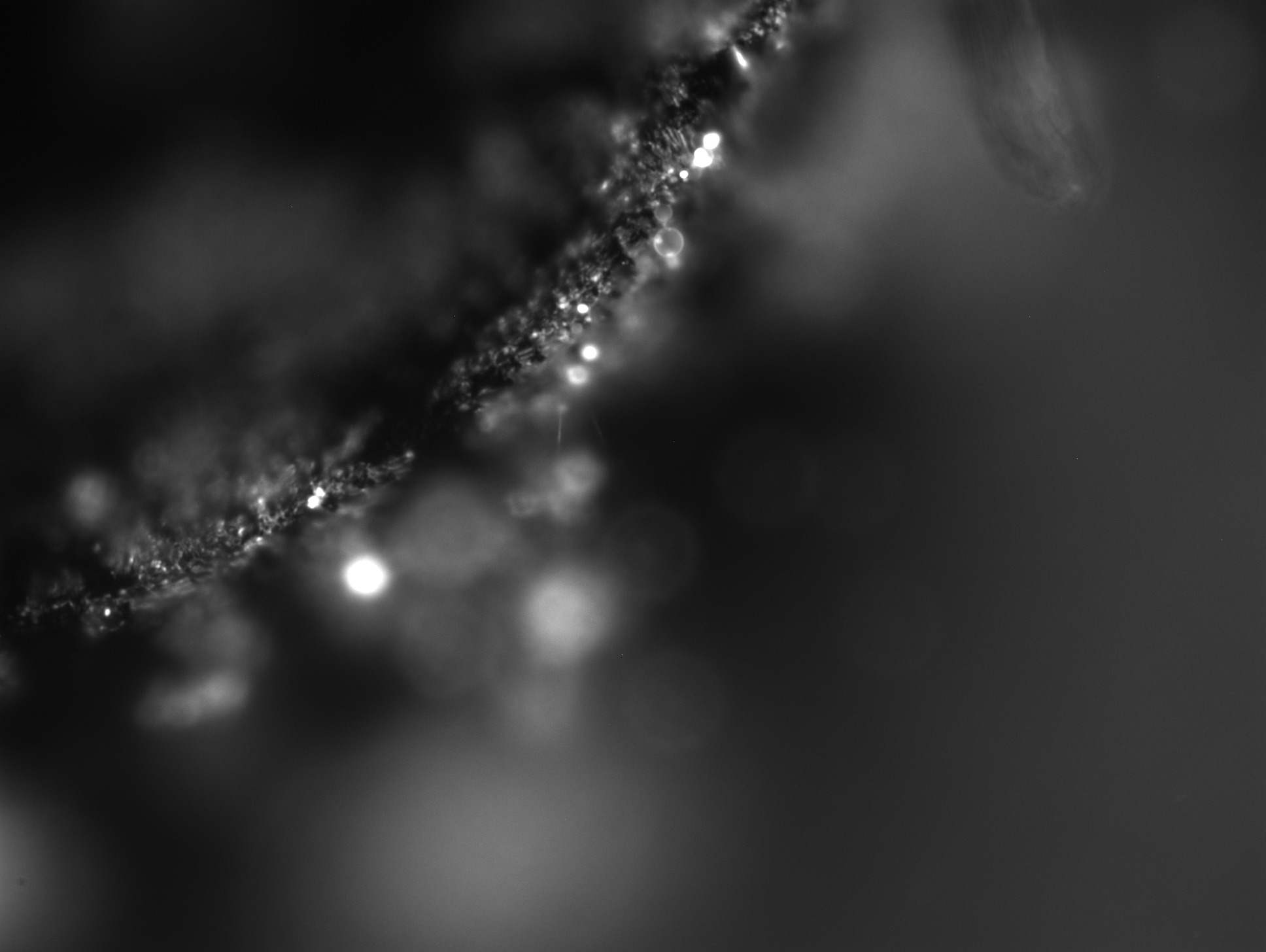

Supplement: Supplementary file 1 — Supplementary Material 1 [file 41598_2026_60022_MOESM1_ESM.zip › SI_Fig5_source images/A9_m3/2_29.742-1.tif]

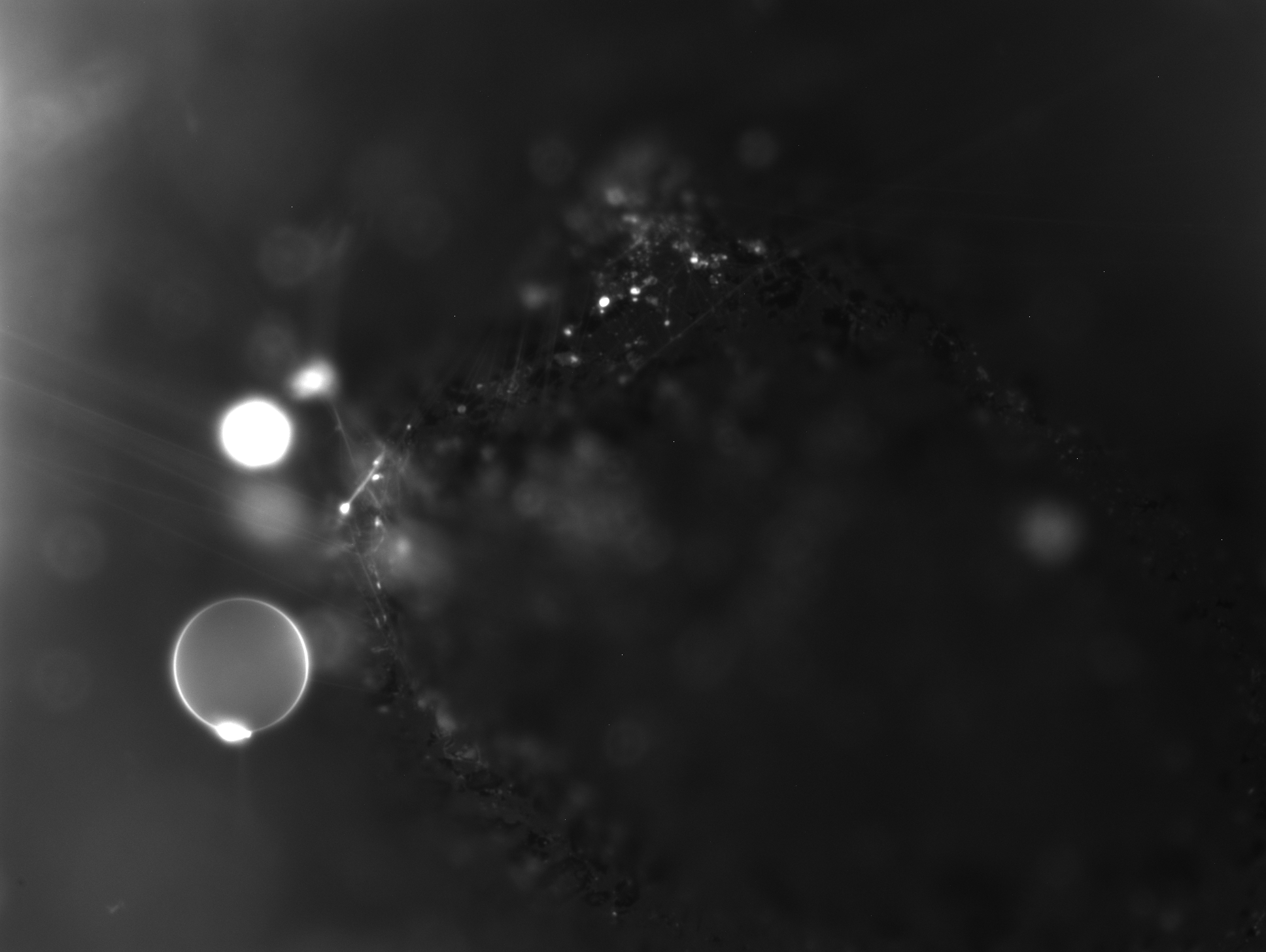

Supplement: Supplementary file 1 — Supplementary Material 1 [file 41598_2026_60022_MOESM1_ESM.zip › SI_Fig5_source images/A9_m3/3_25_58.710.tif]

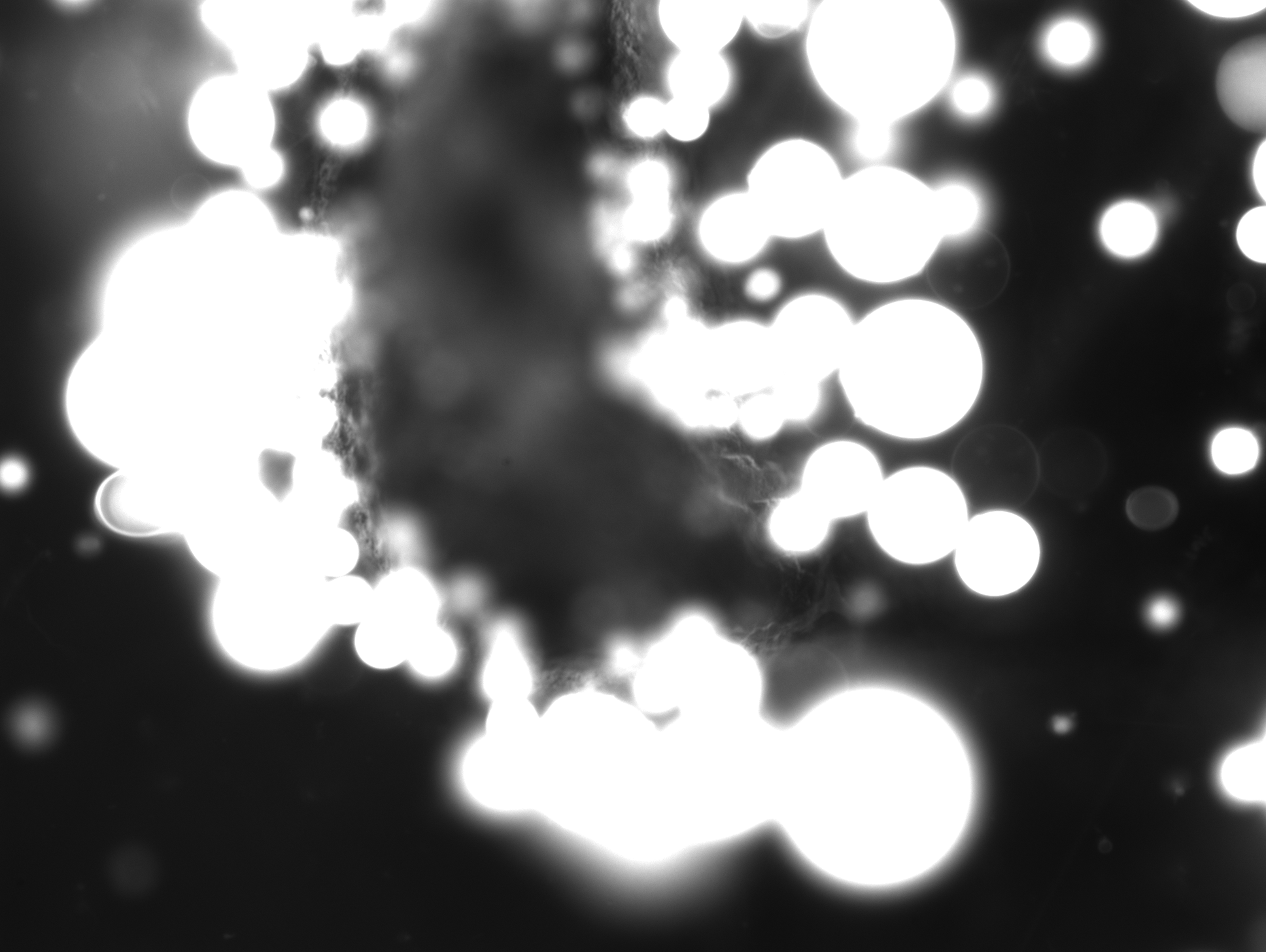

Supplement: Supplementary file 1 — Supplementary Material 1 [file 41598_2026_60022_MOESM1_ESM.zip › SI_Fig5_source images/B1_s4/1_203_30.714.tif]

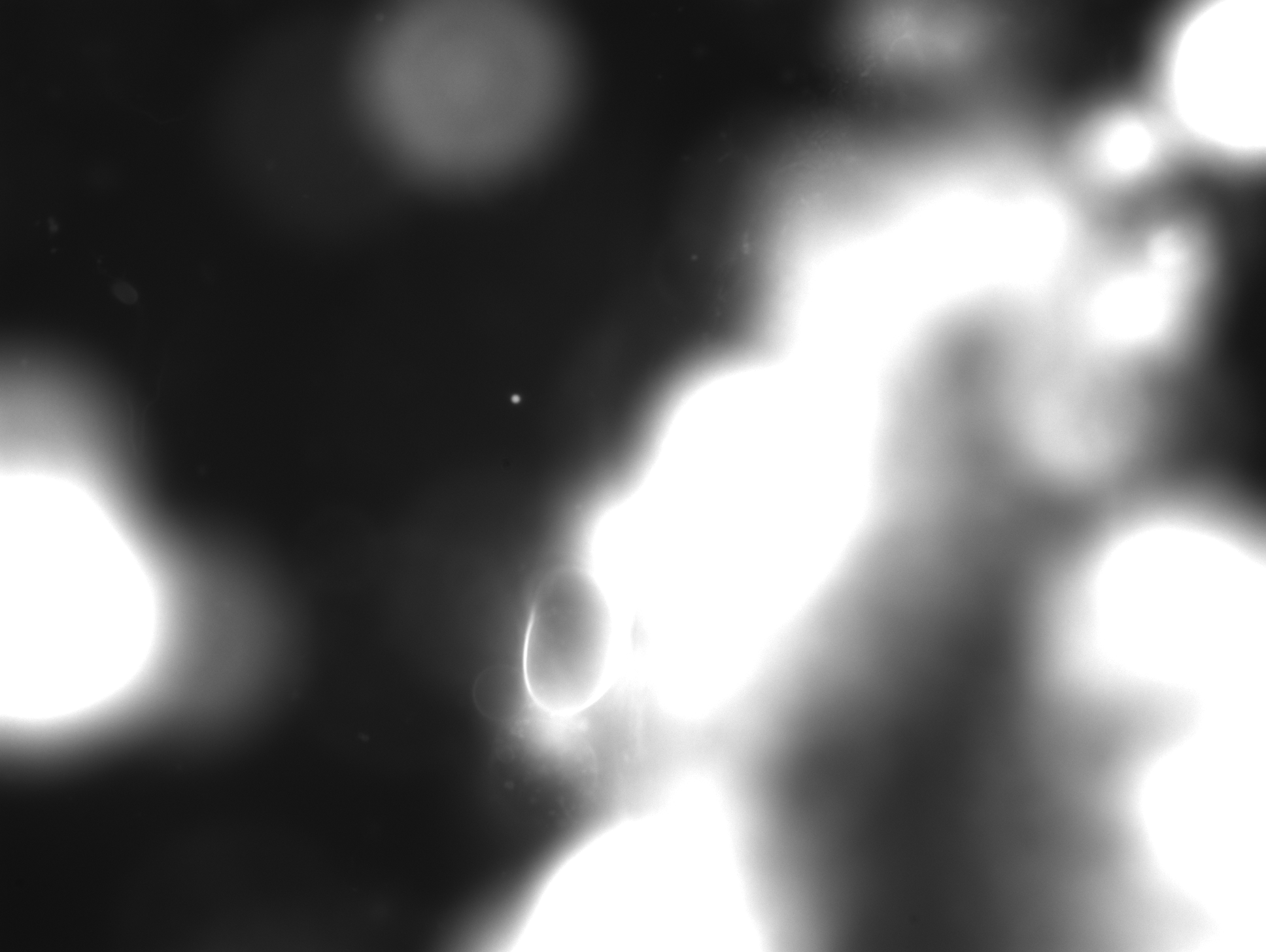

Supplement: Supplementary file 1 — Supplementary Material 1 [file 41598_2026_60022_MOESM1_ESM.zip › SI_Fig5_source images/B1_s4/2_692_30.714.tif]

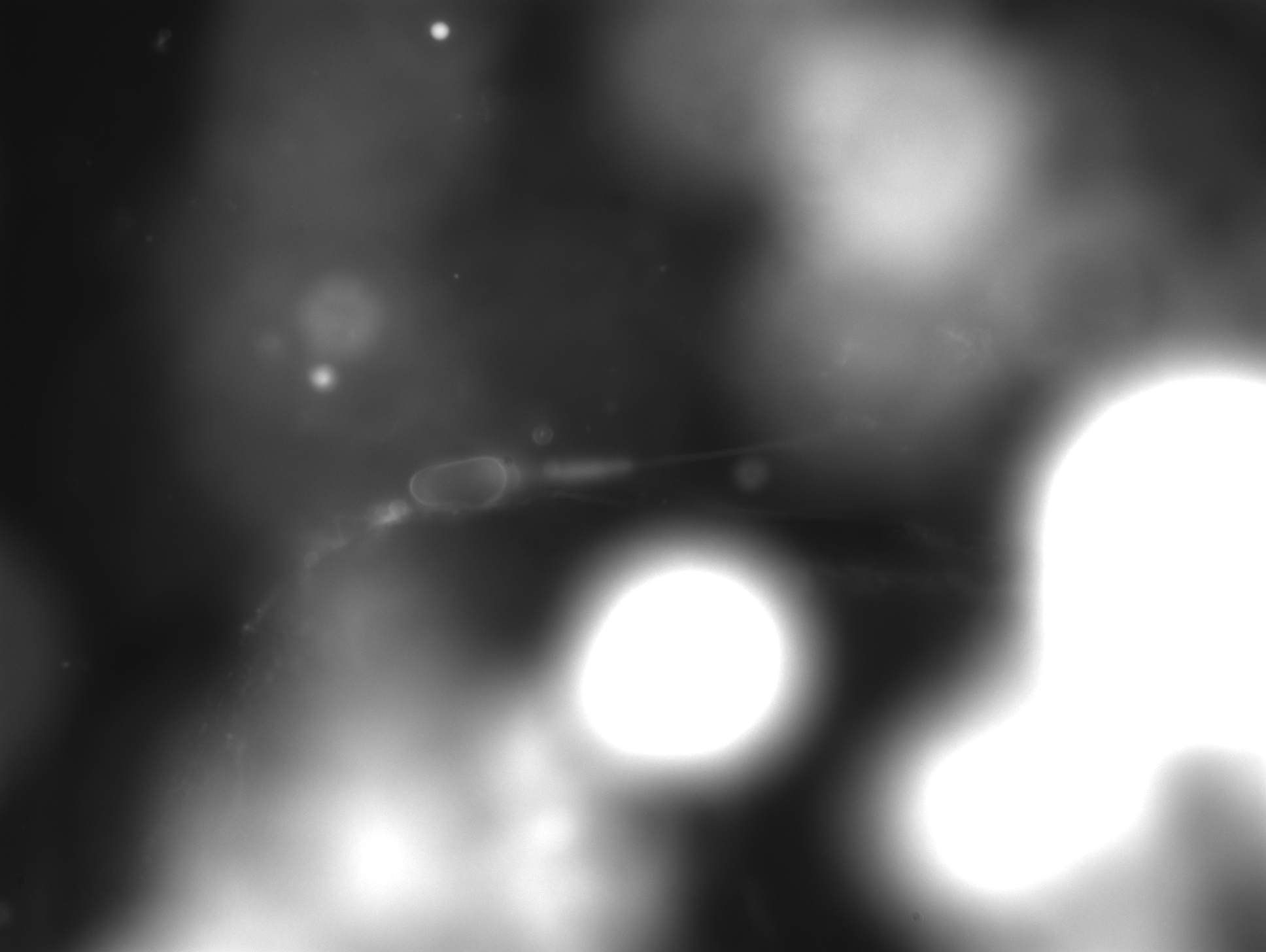

Supplement: Supplementary file 1 — Supplementary Material 1 [file 41598_2026_60022_MOESM1_ESM.zip › SI_Fig5_source images/B1_s4/3_975_30.714.tif]

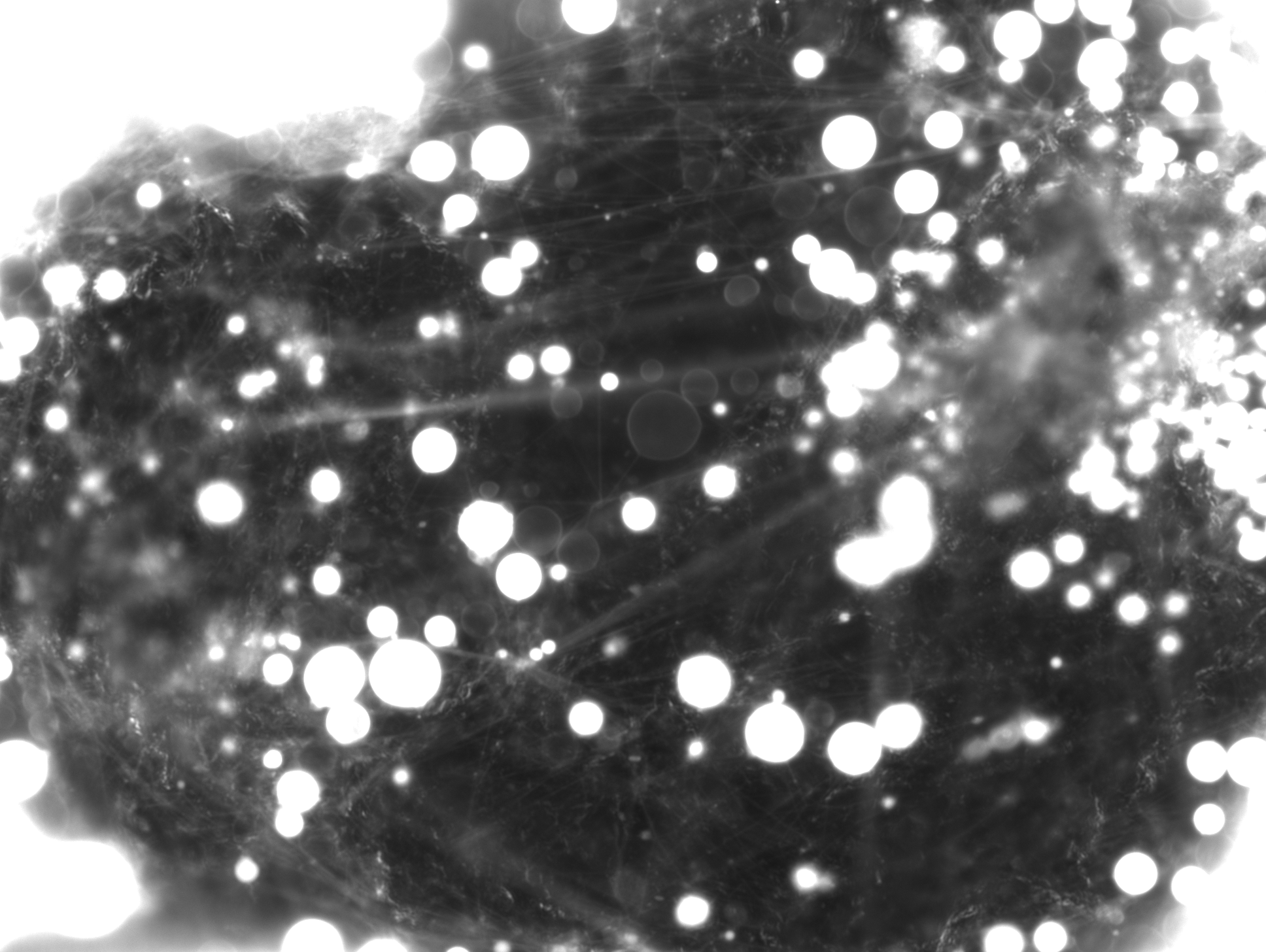

Supplement: Supplementary file 1 — Supplementary Material 1 [file 41598_2026_60022_MOESM1_ESM.zip › SI_Fig5_source images/B2_s5/1_52_54.811.tif]

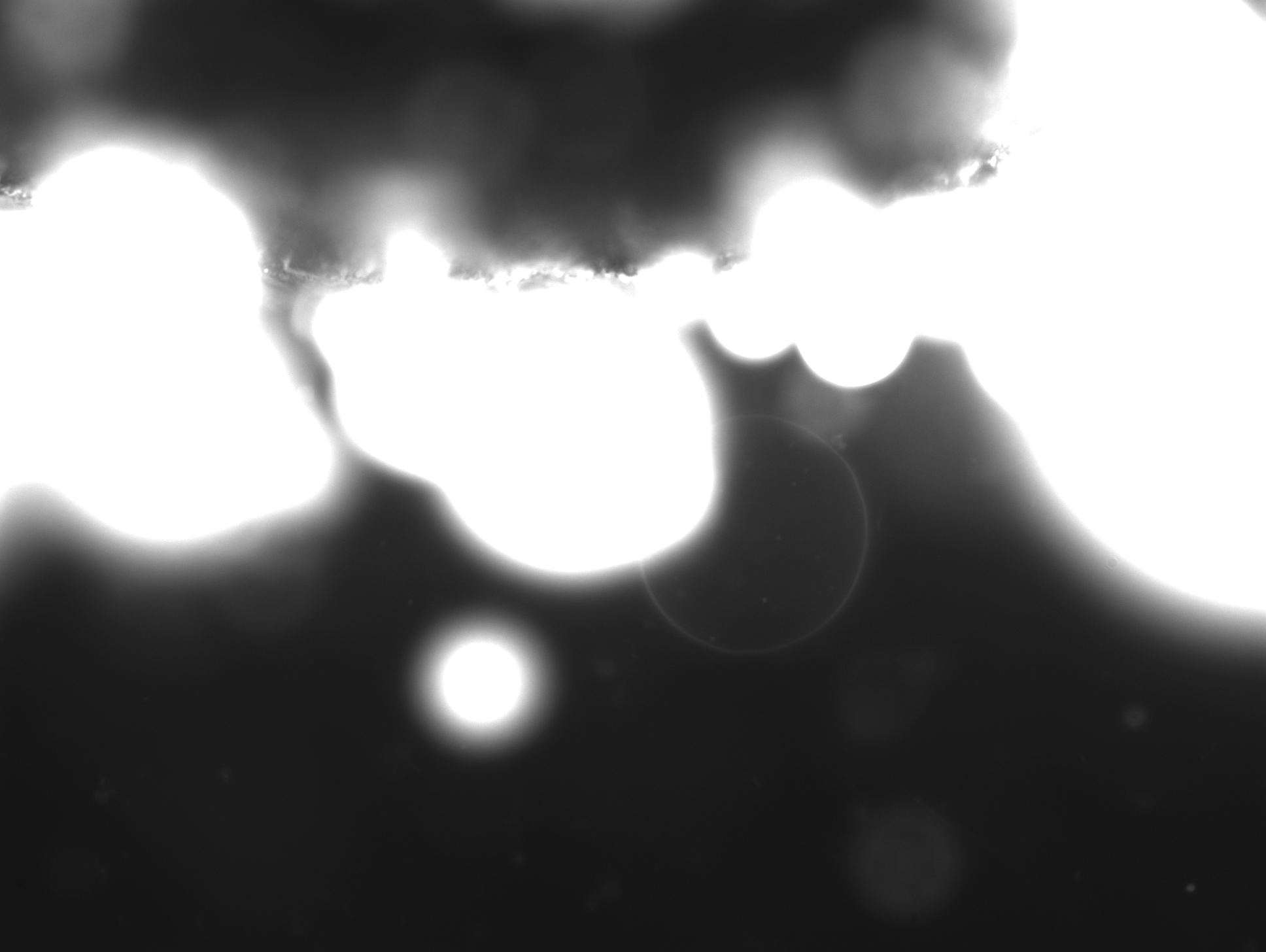

Supplement: Supplementary file 1 — Supplementary Material 1 [file 41598_2026_60022_MOESM1_ESM.zip › SI_Fig5_source images/B2_s5/2_416_54.811.tif]

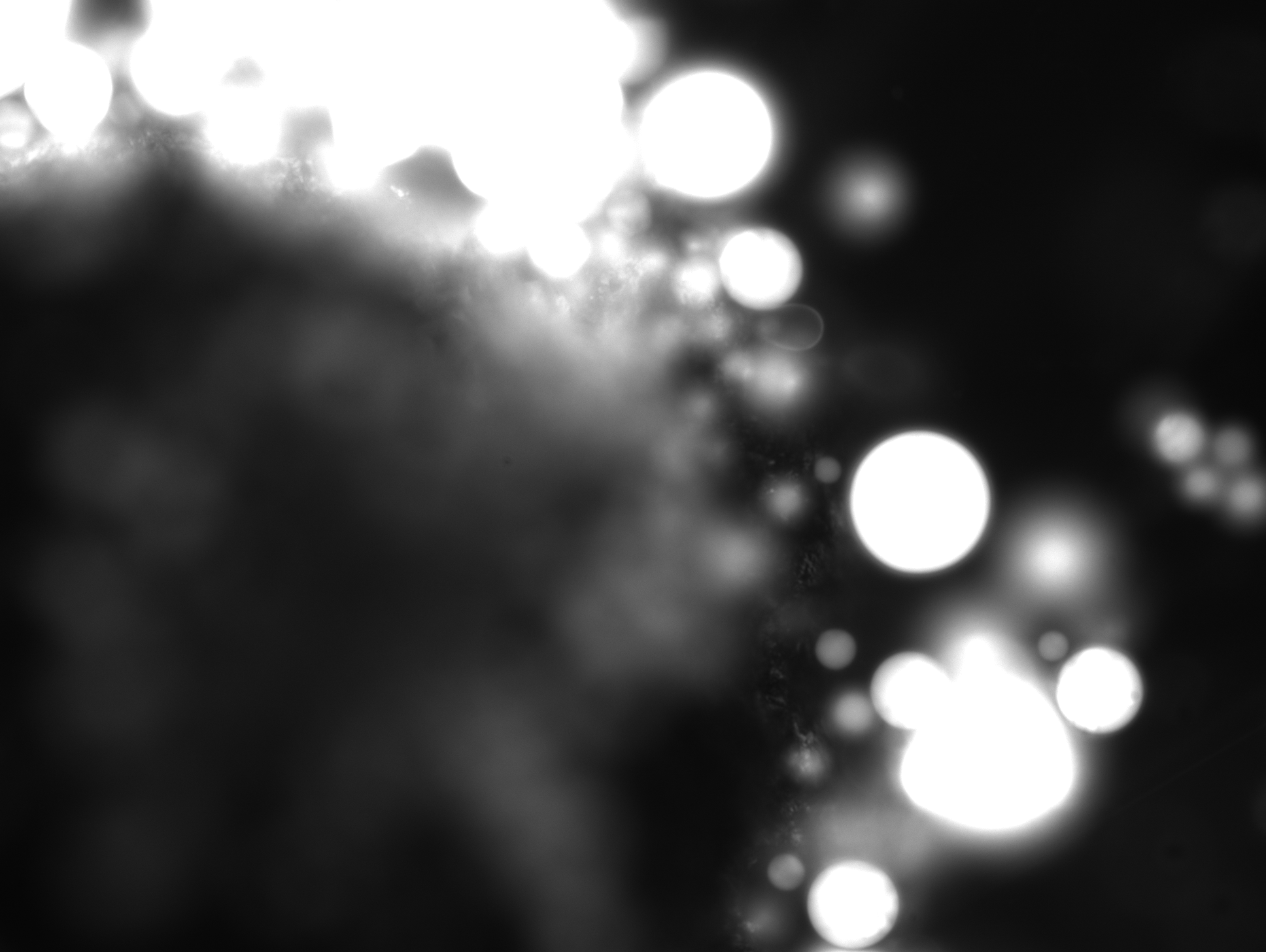

Supplement: Supplementary file 1 — Supplementary Material 1 [file 41598_2026_60022_MOESM1_ESM.zip › SI_Fig5_source images/B2_s5/3_701_30.458.tif]

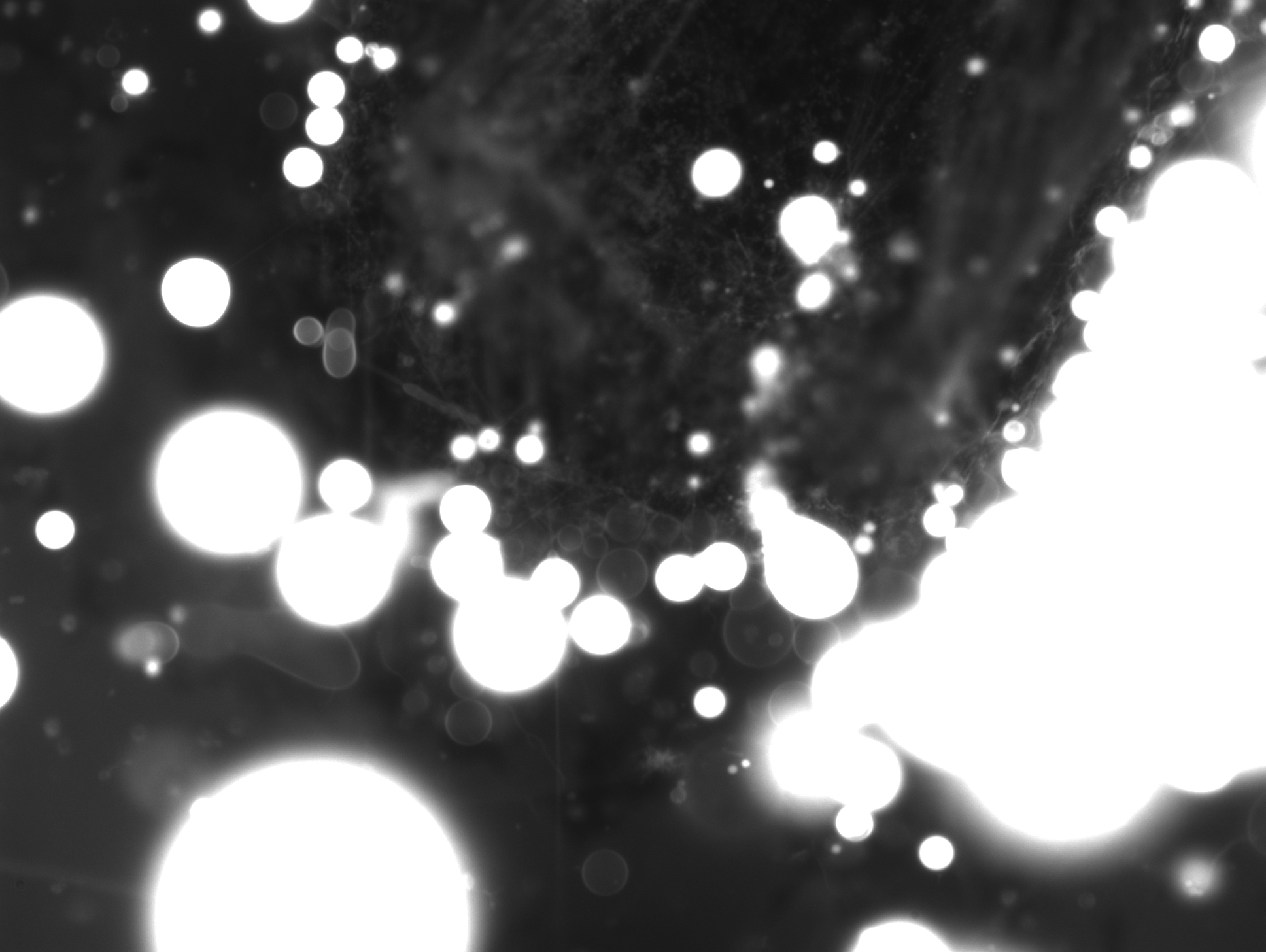

Supplement: Supplementary file 1 — Supplementary Material 1 [file 41598_2026_60022_MOESM1_ESM.zip › SI_Fig5_source images/B3_s6/1_40.002.tif]

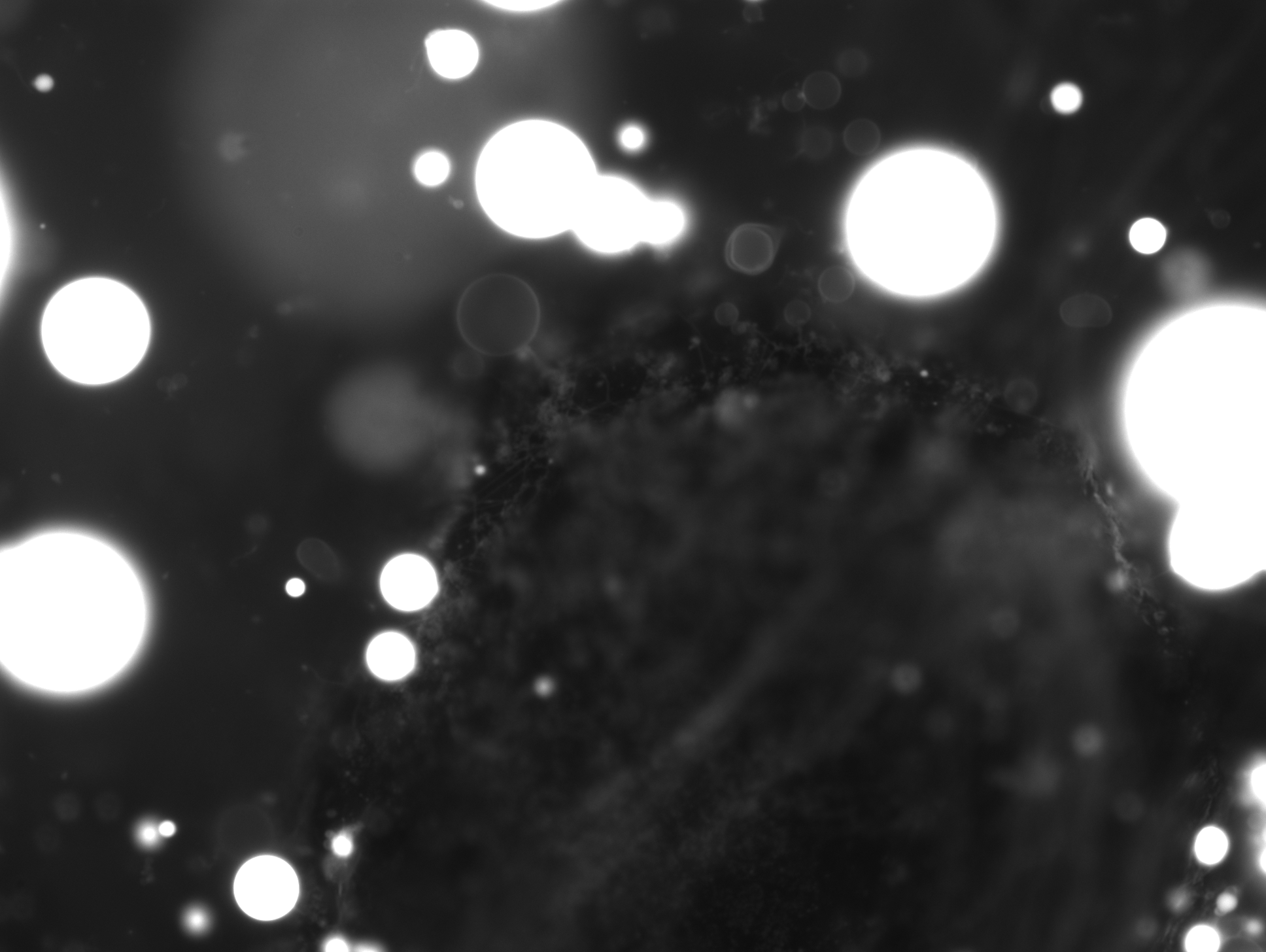

Supplement: Supplementary file 1 — Supplementary Material 1 [file 41598_2026_60022_MOESM1_ESM.zip › SI_Fig5_source images/B3_s6/2_195_03.795.tif]

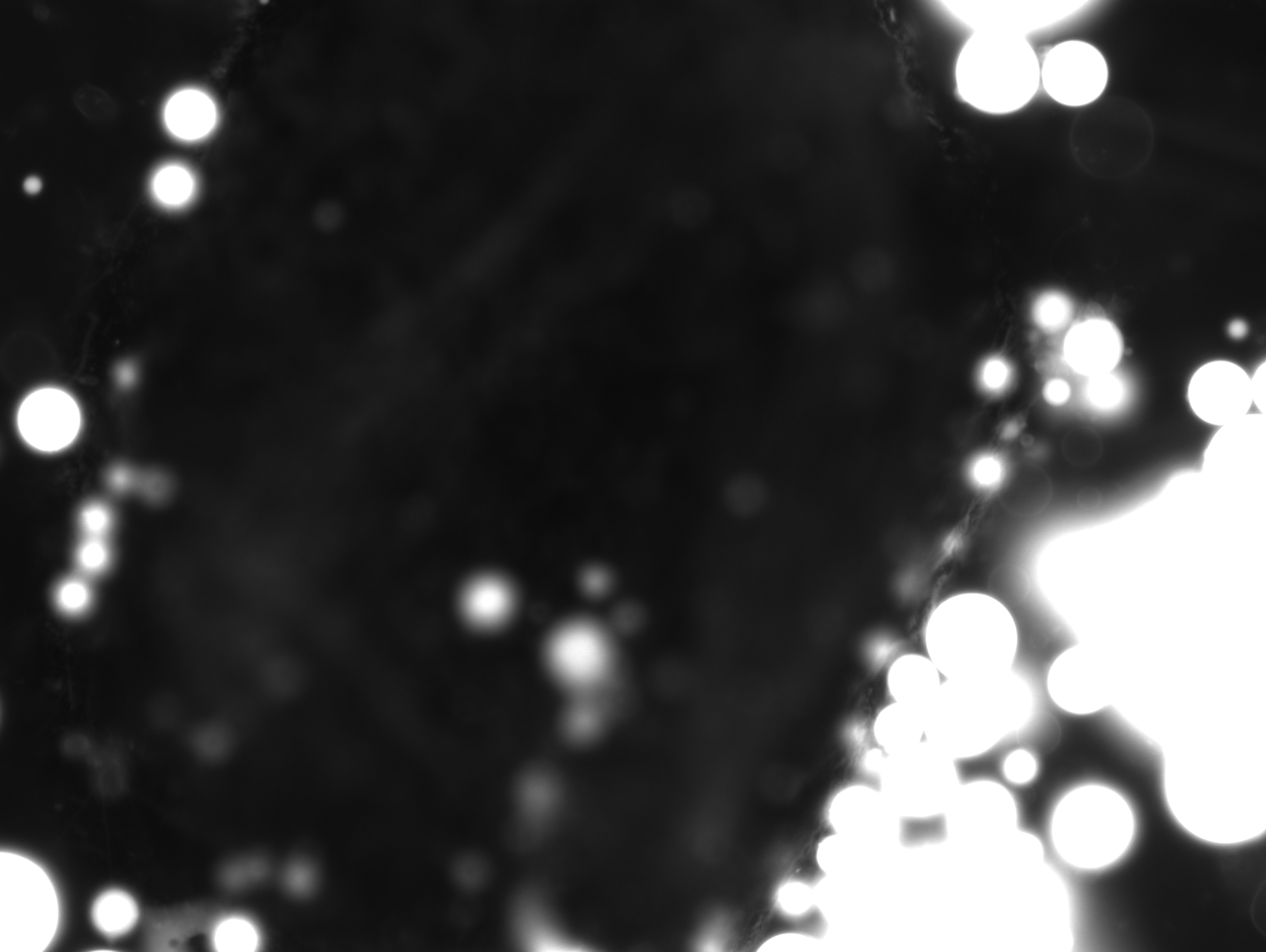

Supplement: Supplementary file 1 — Supplementary Material 1 [file 41598_2026_60022_MOESM1_ESM.zip › SI_Fig5_source images/B3_s6/3_146_27.308.tif]

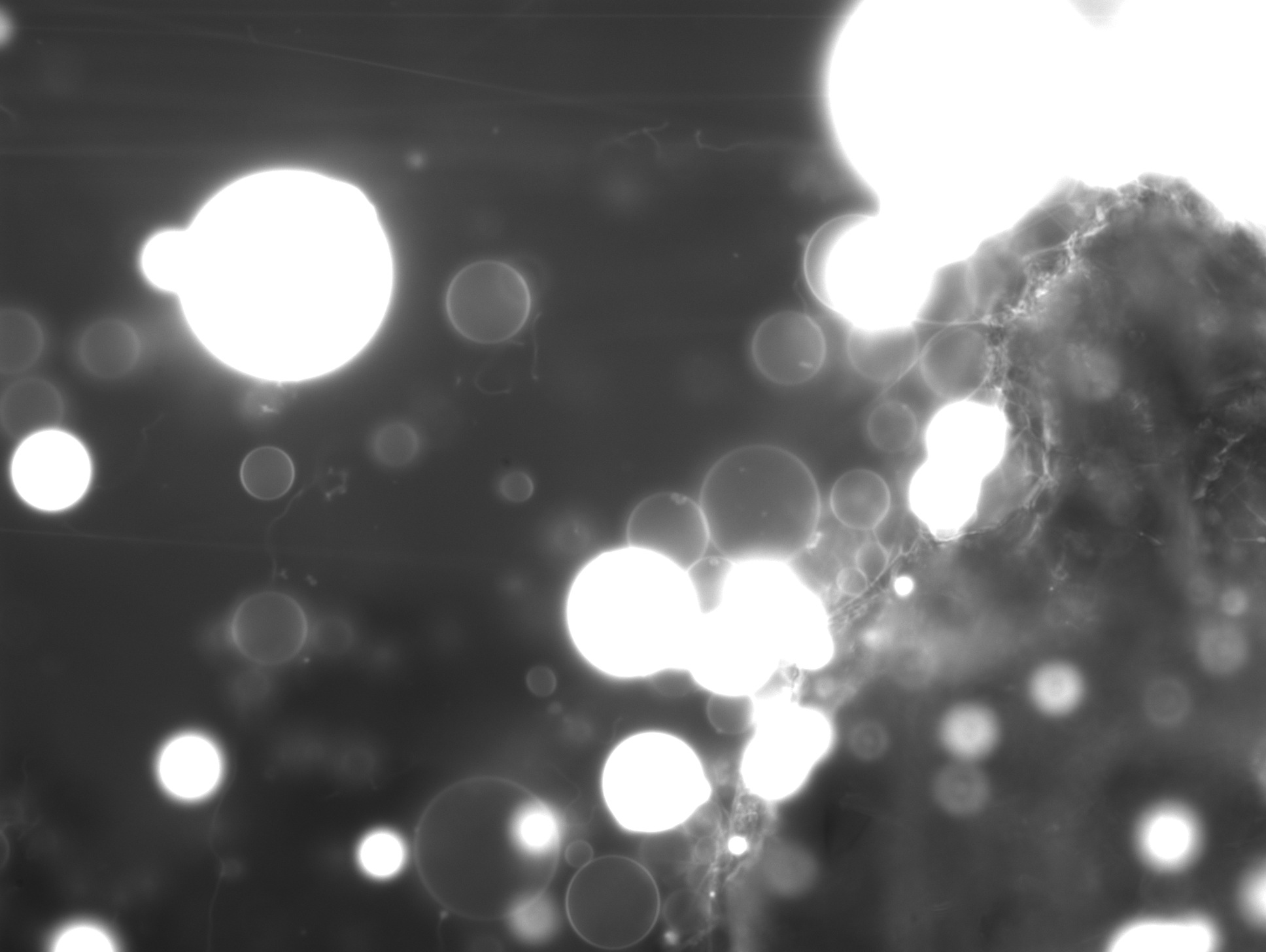

Supplement: Supplementary file 1 — Supplementary Material 1 [file 41598_2026_60022_MOESM1_ESM.zip › SI_Fig5_source images/B4_p1/1_16_39.580.tif]

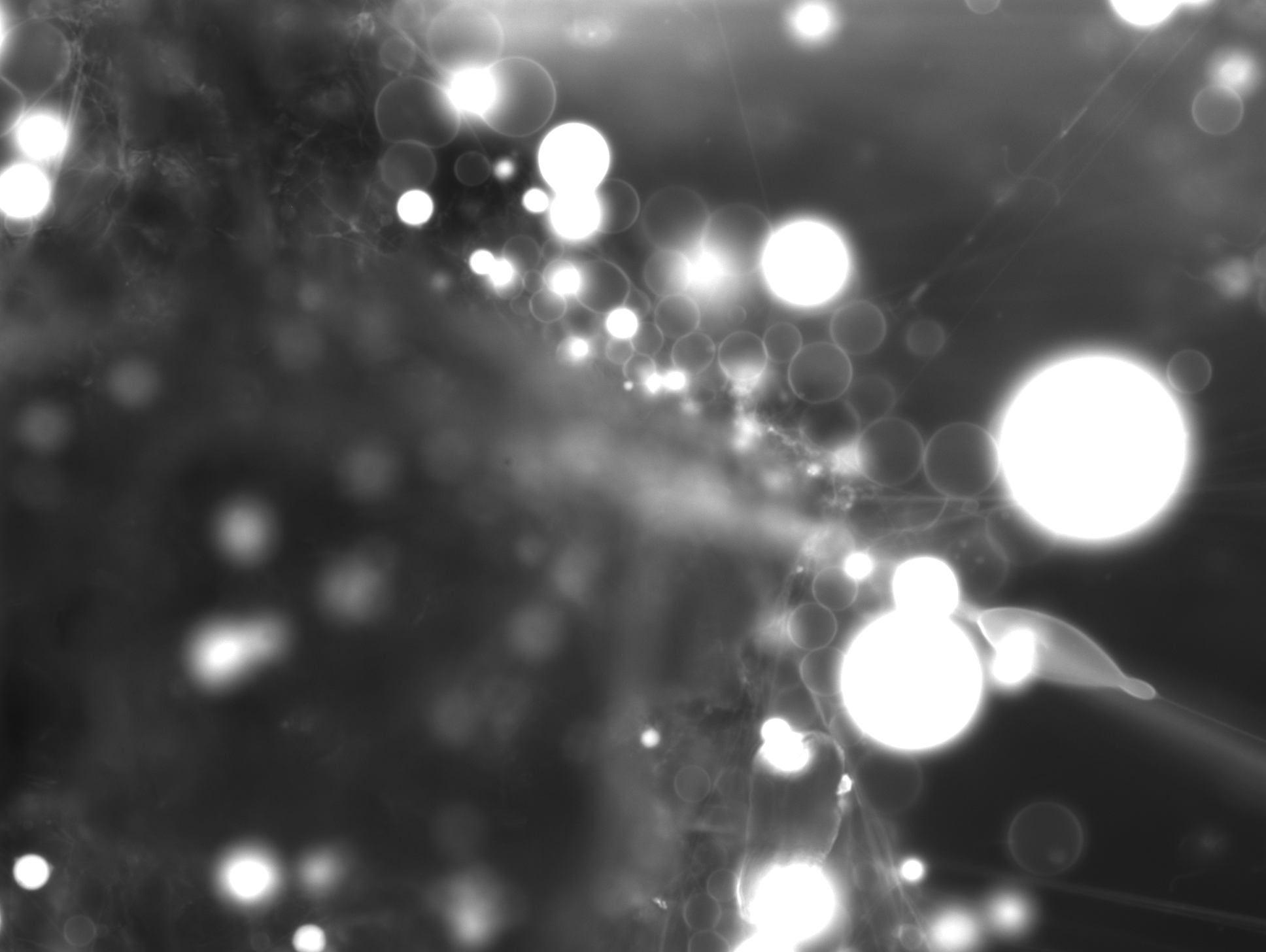

Supplement: Supplementary file 1 — Supplementary Material 1 [file 41598_2026_60022_MOESM1_ESM.zip › SI_Fig5_source images/B4_p1/2_17.188.tif]

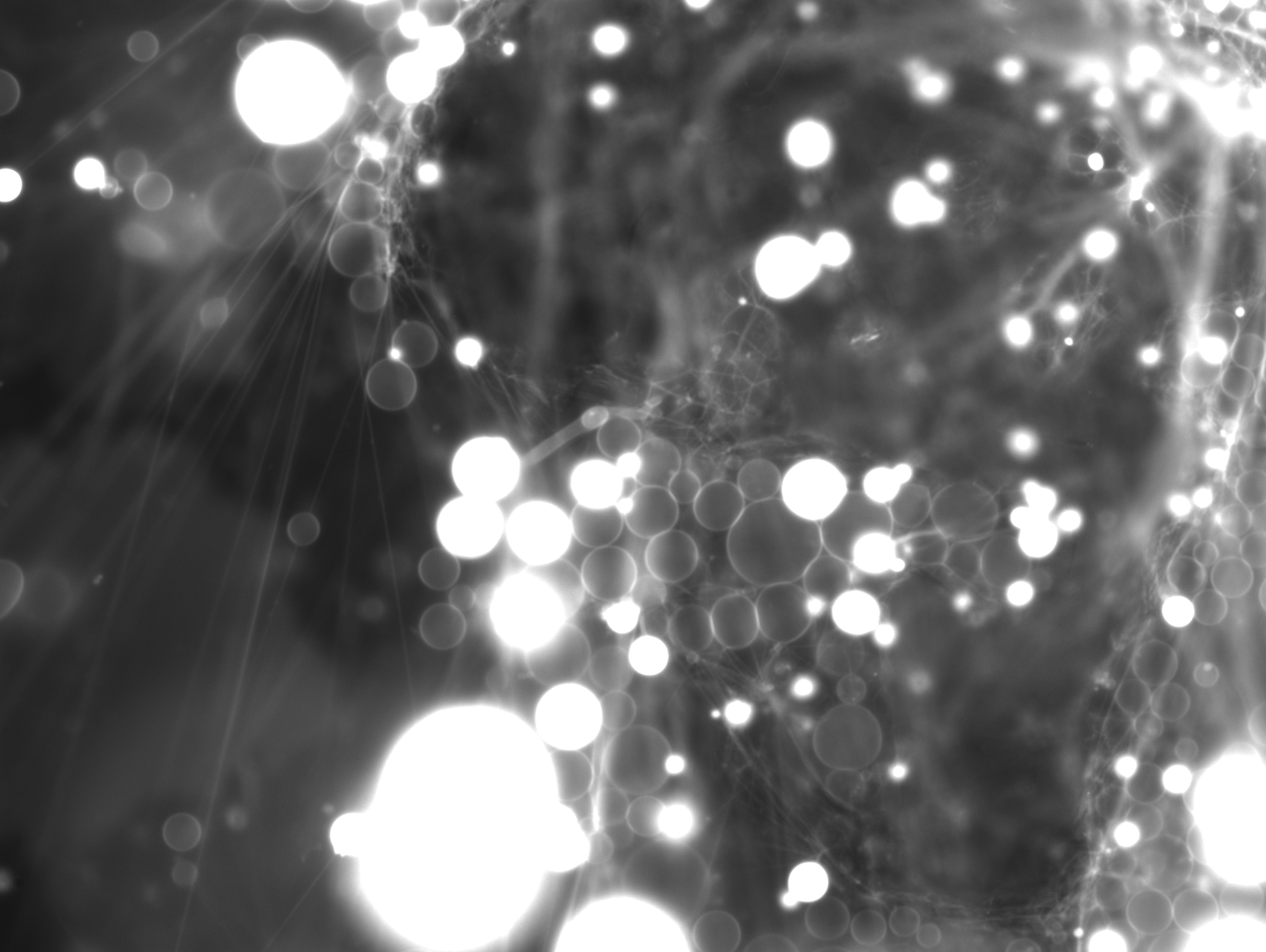

Supplement: Supplementary file 1 — Supplementary Material 1 [file 41598_2026_60022_MOESM1_ESM.zip › SI_Fig5_source images/B4_p1/3_94_21.620.tif]
