# Supplementary material for: Protocell formation on micrometeorites
Source: Sci Rep. 2026 Jul 11;16:21833. doi: 10.1038/s41598-026-60022-x (PMC13365509; doi:10.1038/s41598-026-60022-x)
Supplement: Supplementary file 2 — Supplementary Material 2 [file 41598_2026_60022_MOESM2_ESM.pdf]

Supporting Information

Protocell formation on micrometeorites

Aldo Jesorka[1,2], Esteban Pedrueza Villalmanzo[3], Ezgi Ciftcioglu[2], Piotr Jedrasik[3], Jon Larsen[4], Irep Gözen[1]*

[1]GOMOD AB, Göteborg, Sweden
[2]Department of Chemistry and Chemical Engineering, Chalmers University of Technology, SE-412 96, Gothenburg, Sweden
[3]Department of Microtechnology and Nanoscience, Nanofabrication Laboratory (MC2), Chalmers University of Technology, SE-412 58, Gothenburg, Sweden
[4]Project Stardust, Oslo, Norway

*Corresponding author, E-mail: irep.gozen@gomod.eu

1. Extended Fig. 6

Complete images corresponding to the panels in Fig. 6 are shown below. The panels are marked in yellow frames in the image and the contour of the particles are shown by orange dashed lines.

Full image corresponding to Fig. 6A:

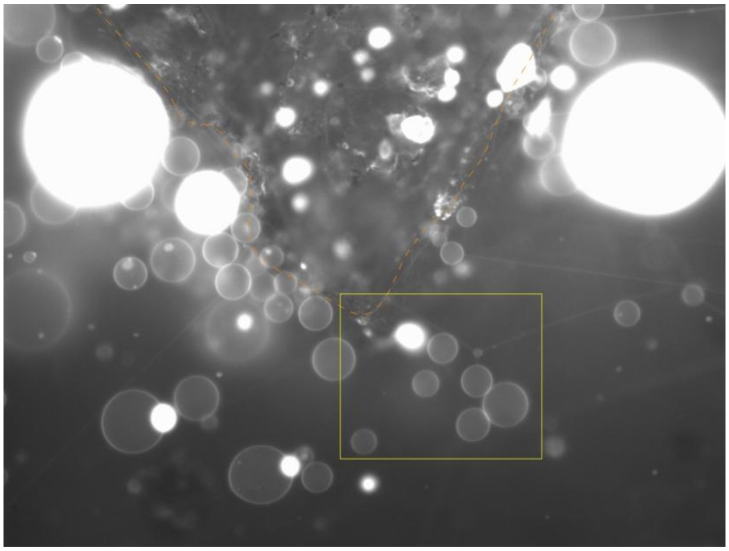

Full image corresponding to Fig. 6B and 6C:

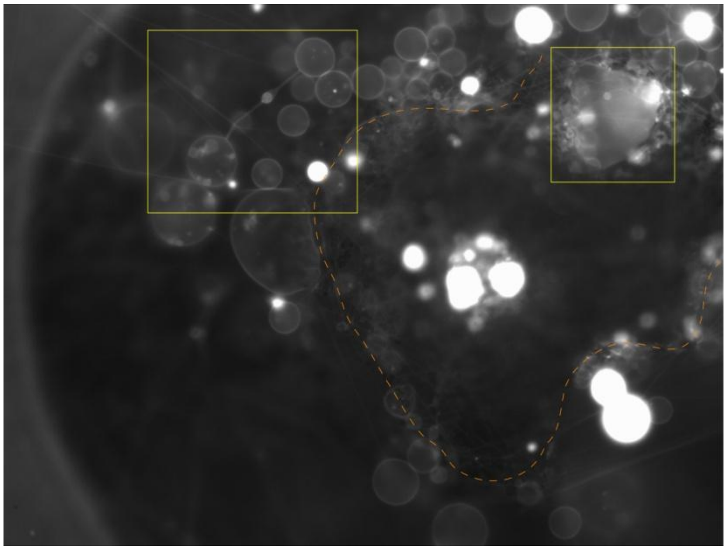

Full image corresponding to Fig. 6D:

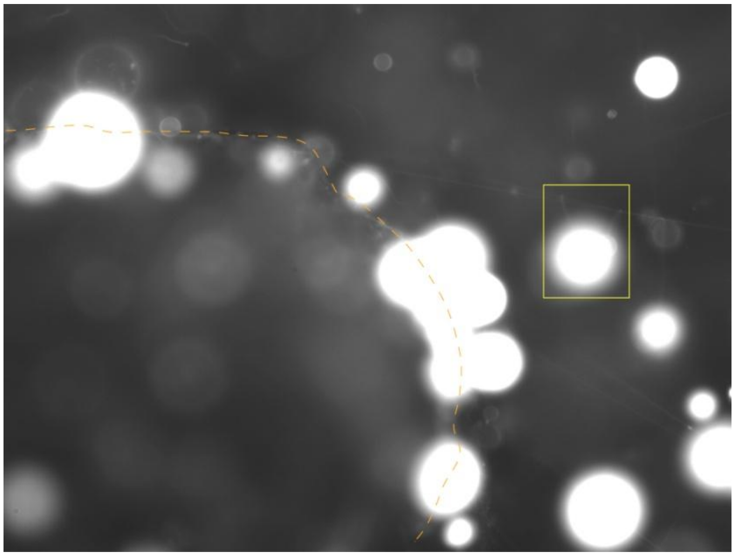

Full image corresponding to Fig. 6F:

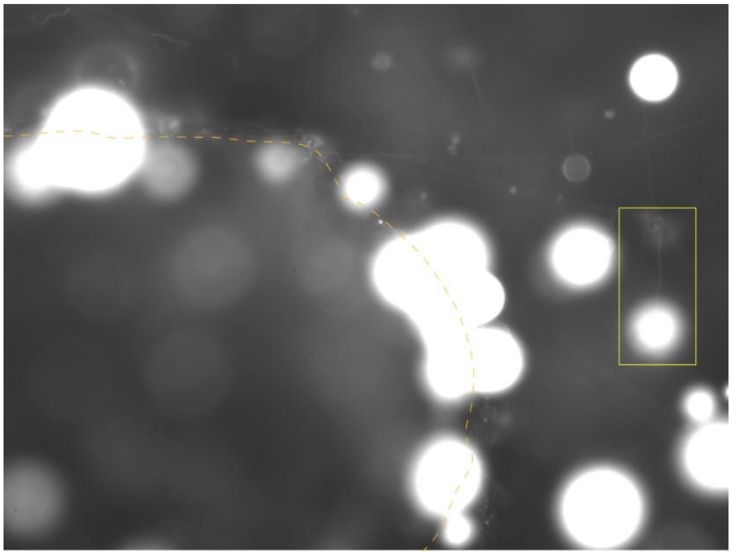

2. Analyses of all particles used in the experiments

In this section the SEM images of reference particles, raw EDX spectra and analysis data for all particles are provided:

Micrometeorite 1: Corresponds to the micrometeorite shown in Fig. 2 panels B-E-H

- Exposure to archaeal lipids: Fig. 5A - m1
- Exposure to E.coli lipids: Fig. 5B - m1
- Exposure to plant lipids mixture: Fig. 5C - m1

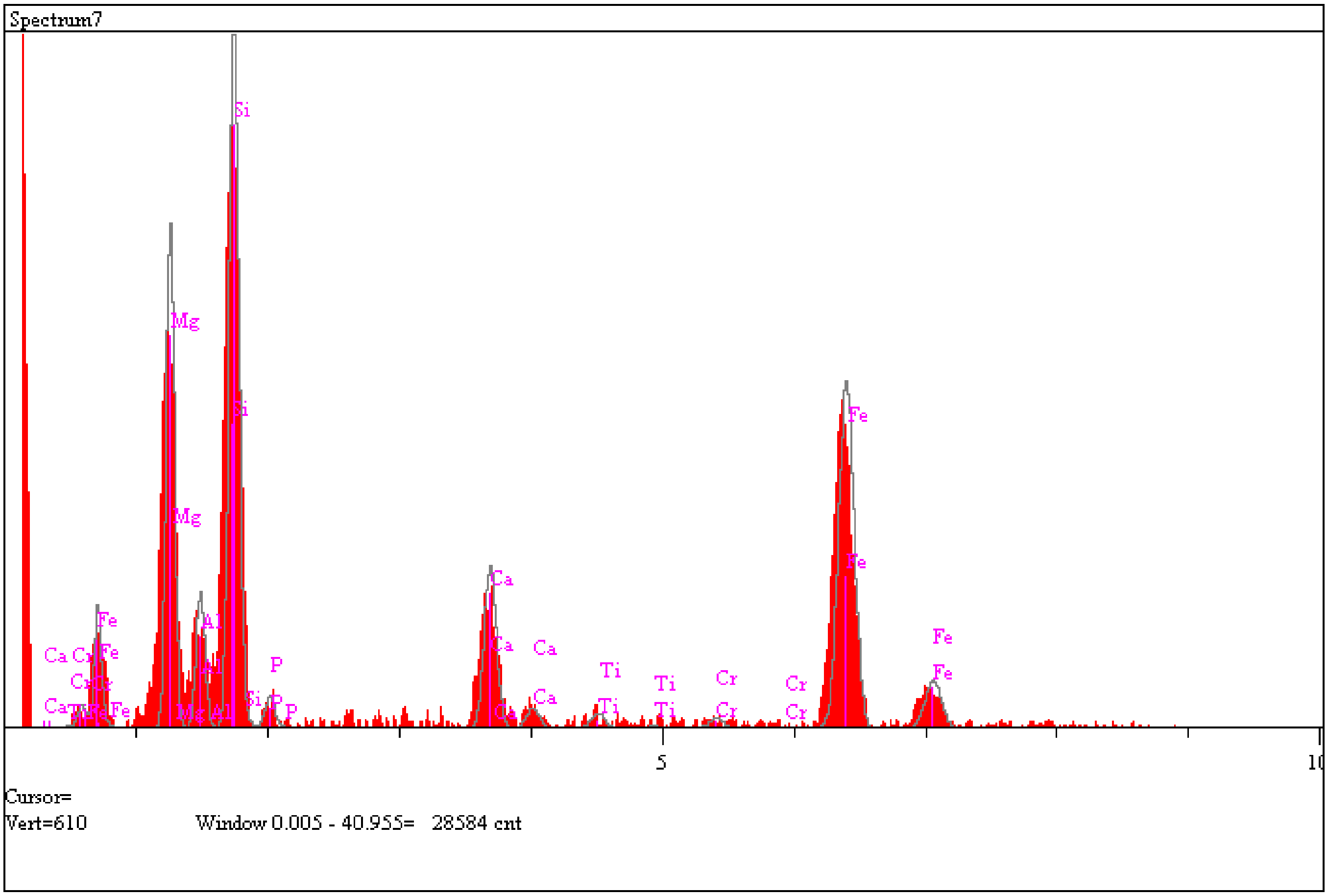

Elt.	Line	Intensity (c/s)	Error 2-sig	Atomic %	K-Ratio	
N	Ka	0.00	0.000	0.000	0.0000	
Mg	Ka	57.90	1.965	11.256	0.0421	
Al	Ka	16.28	1.042	2.793	0.0129	
Si	Ka	99.00	2.569	15.808	0.0876	
P	Ka	4.32	0.537	0.697	0.0044	
Ca	Ka	27.25	1.348	5.424	0.0550	
Ti	Ka	2.66	0.421	0.673	0.0079	
Cr	Ka	2.00	0.365	0.690	0.0099	

Fe	Ka	74.05	2.222	62.659	0.7802	
				100.000		Total

Micrometeorite 2: Corresponds to the micrometeorite shown in Fig. 2 panels A-D-G

- Exposure to archaeal lipids: Fig. 5A – m2
- Exposure to E.coli lipids: Fig. 5B – m2
- Exposure to plant lipids mixture: Fig. 5C – m2
- Fluorescence micrograph in Fig. 3I and Fig. 3G corresponds to this particle.

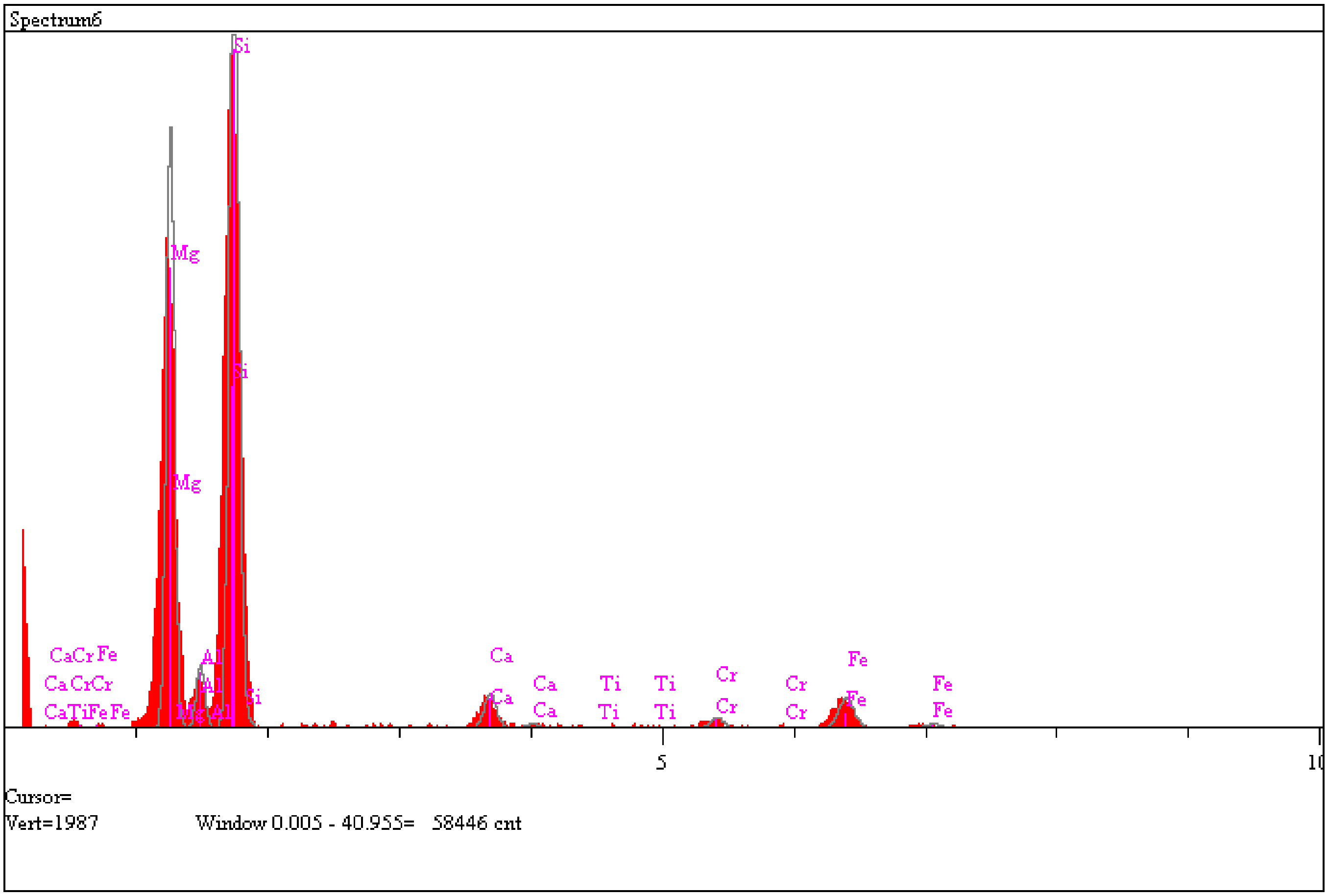

Elt.	Line	Intensity	Error	Atomic	K-Ratio	

		(c/s)	2-sig	%		
Mg	Ka	224.03	3.864	28.483	0.1992	
Al	Ka	24.54	1.279	3.203	0.0238	
Si	Ka	360.64	4.903	45.666	0.3907	
Ca	Ka	18.72	1.117	3.308	0.0462	
Ti	Ka	0.69	0.215	0.162	0.0025	
Cr	Ka	6.57	0.662	2.308	0.0399	
Fe	Ka	21.18	1.188	15.539	0.2731	
Ni	Ka	0.57	0.194	1.330	0.0245	
				100.000		Total

Micrometeorite 3: Corresponds to the micrometeorite shown in Fig. 2 panels C-F-I

- Exposure to archaeal lipids: Fig. 5A – m3
- Exposure to E.coli lipids: Fig. 5B – m3
- Exposure to plant lipids mixture: Fig. 5C – m3
- Fluorescence micrograph in Fig. 3H corresponds to this particle.

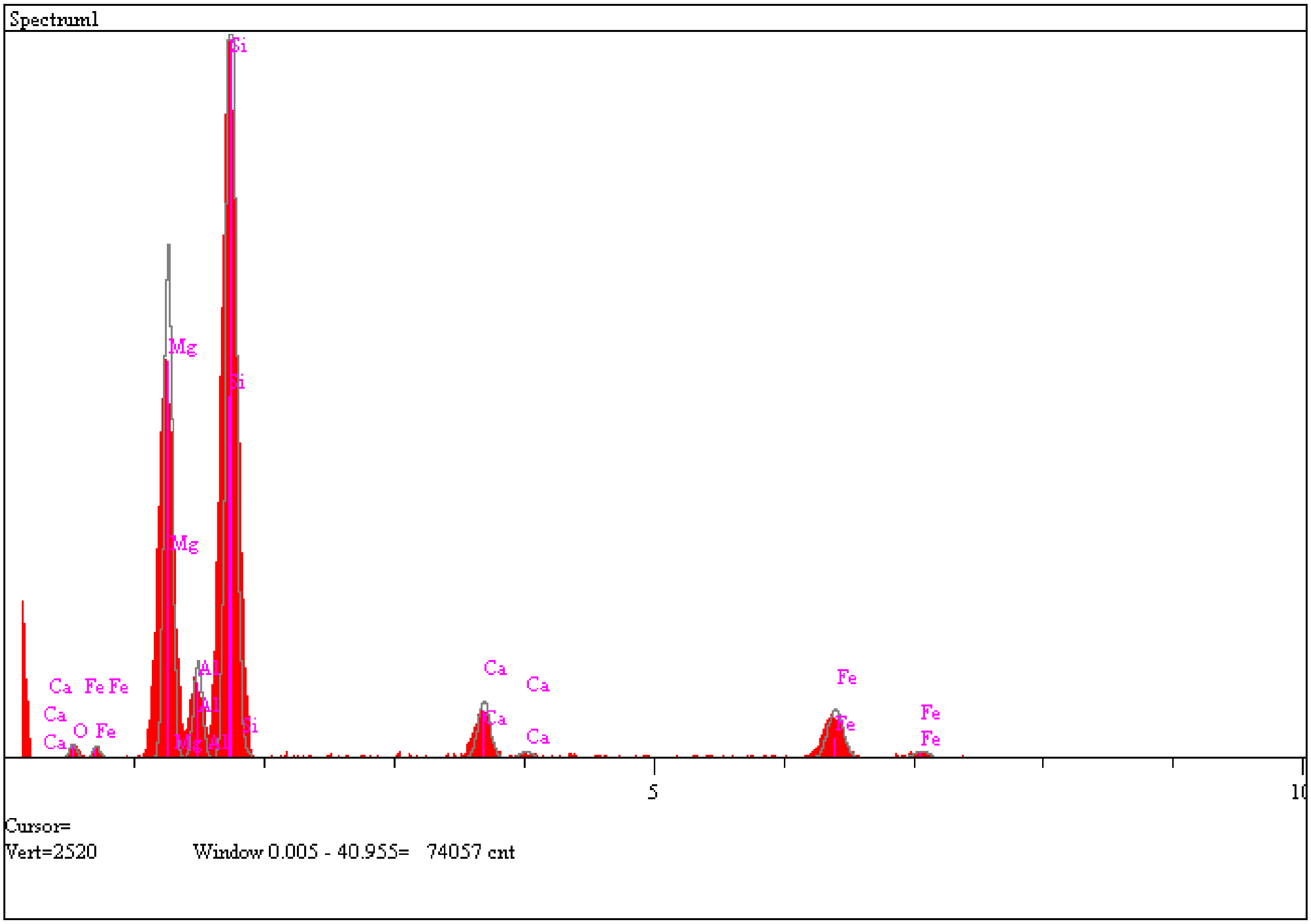

Elt.	Line	Intensity (c/s)	Error 2-sig	Atomic %	K-Ratio	
N	Ka	0.00	0.000	0.000	0.0000	
O	Ka	5.32	0.596	1.565	0.0049	
Mg	Ka	232.28	3.935	22.709	0.1486	
Al	Ka	45.65	1.744	4.408	0.0319	
Si	Ka	466.27	5.575	43.874	0.3634	
Ca	Ka	38.12	1.594	4.973	0.0677	
Fe	Ka	41.36	1.660	22.471	0.3836	

				100.000		Total

Model Micrometeorite 1:

- Exposure to archaeal lipids: Fig. 5A – p1
- Exposure to E.coli lipids: Fig. 5B – p1
- Exposure to plant lipids mixture: Fig. 5C – p1
- Fluorescence micrograph in Fig. 3E corresponds to this particle.

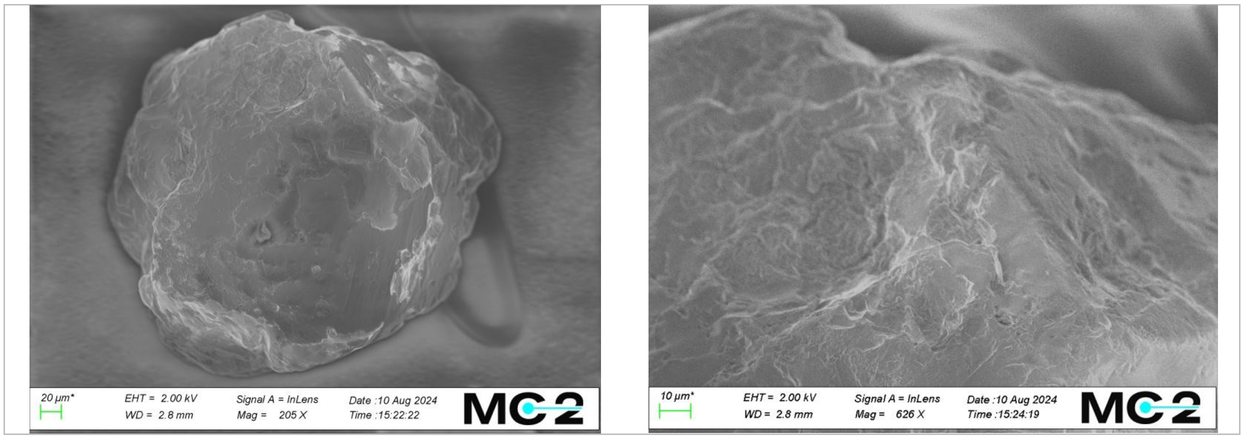

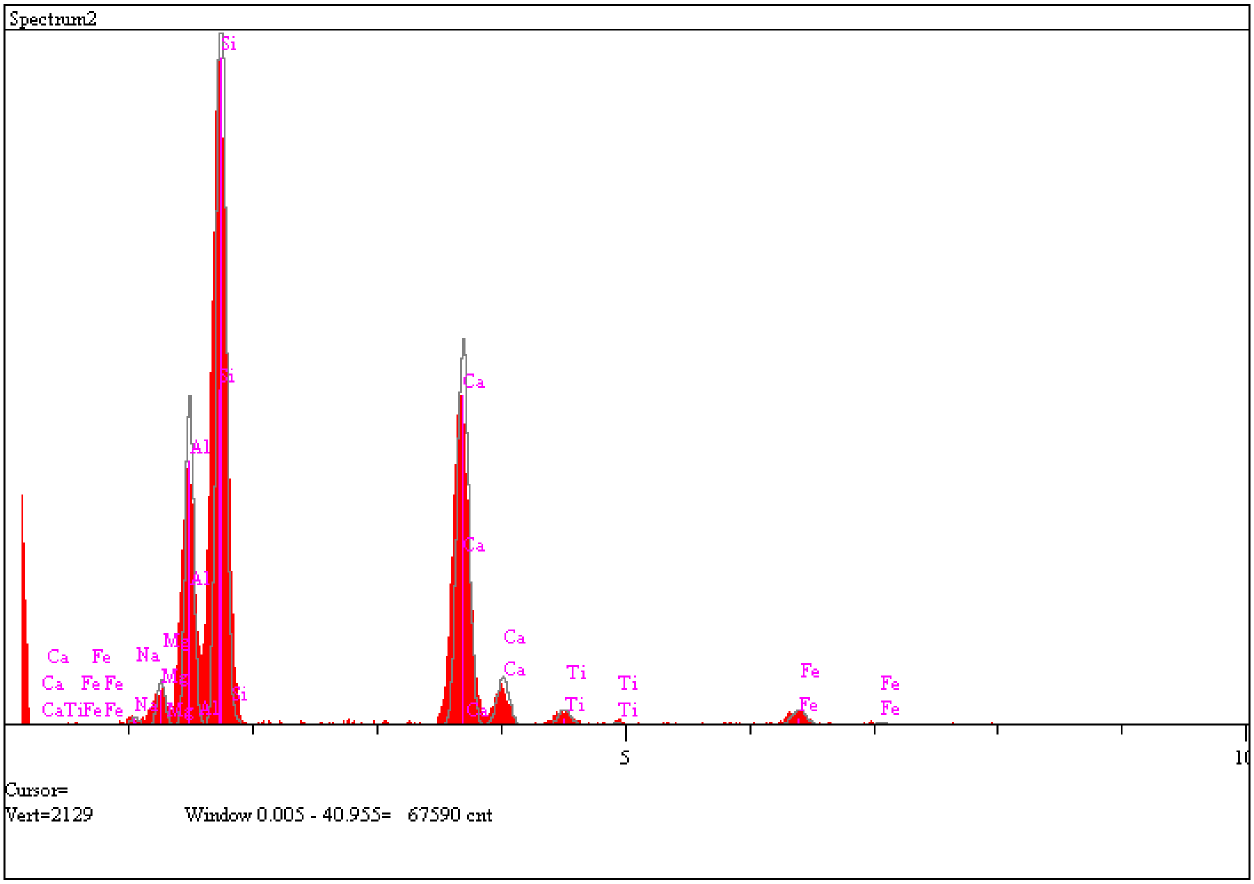

Elt.	Line	Intensity (c/s)	Error 2-sig	Atomic %	K-Ratio	
Na	Ka	3.35	0.472	0.448	0.0022	
Mg	Ka	17.89	1.092	1.922	0.0121	
Al	Ka	137.26	3.025	13.628	0.1012	
Si	Ka	381.74	5.044	39.625	0.3137	
Ca	Ka	227.95	3.898	34.640	0.4268	
Ti	Ka	10.20	0.825	2.151	0.0281	
Fe	Ka	11.85	0.889	7.586	0.1159	
				100.000		Total

Model Micrometeorite 2:

- Exposure to archaeal lipids: Fig. 5A – p2
- Exposure to E.coli lipids: Fig. 5B – p2
- Exposure to plant lipids mixture: Fig. 5C – p2
- Fluorescence micrograph in Fig. 3D corresponds to this particle.

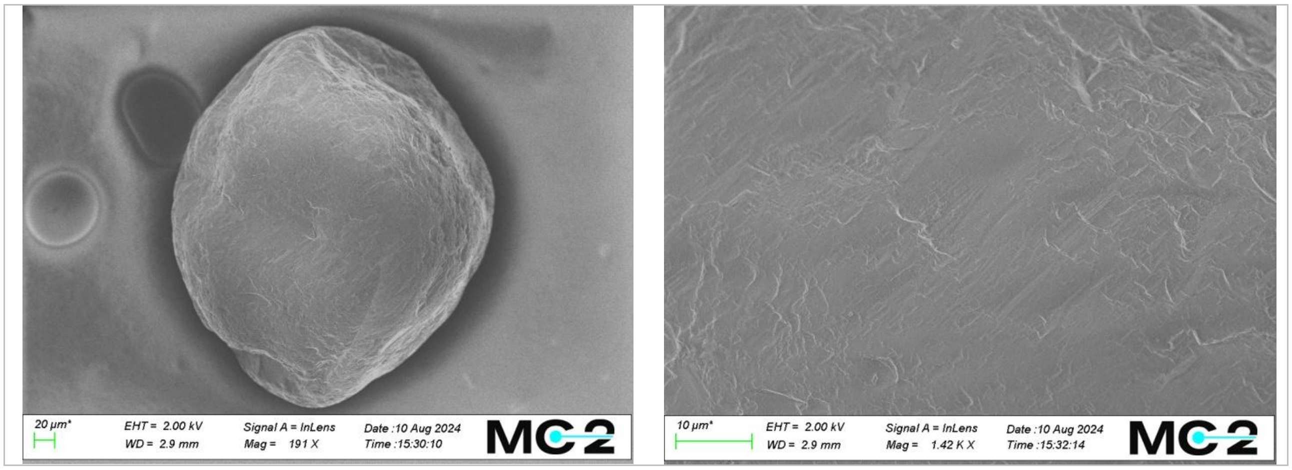

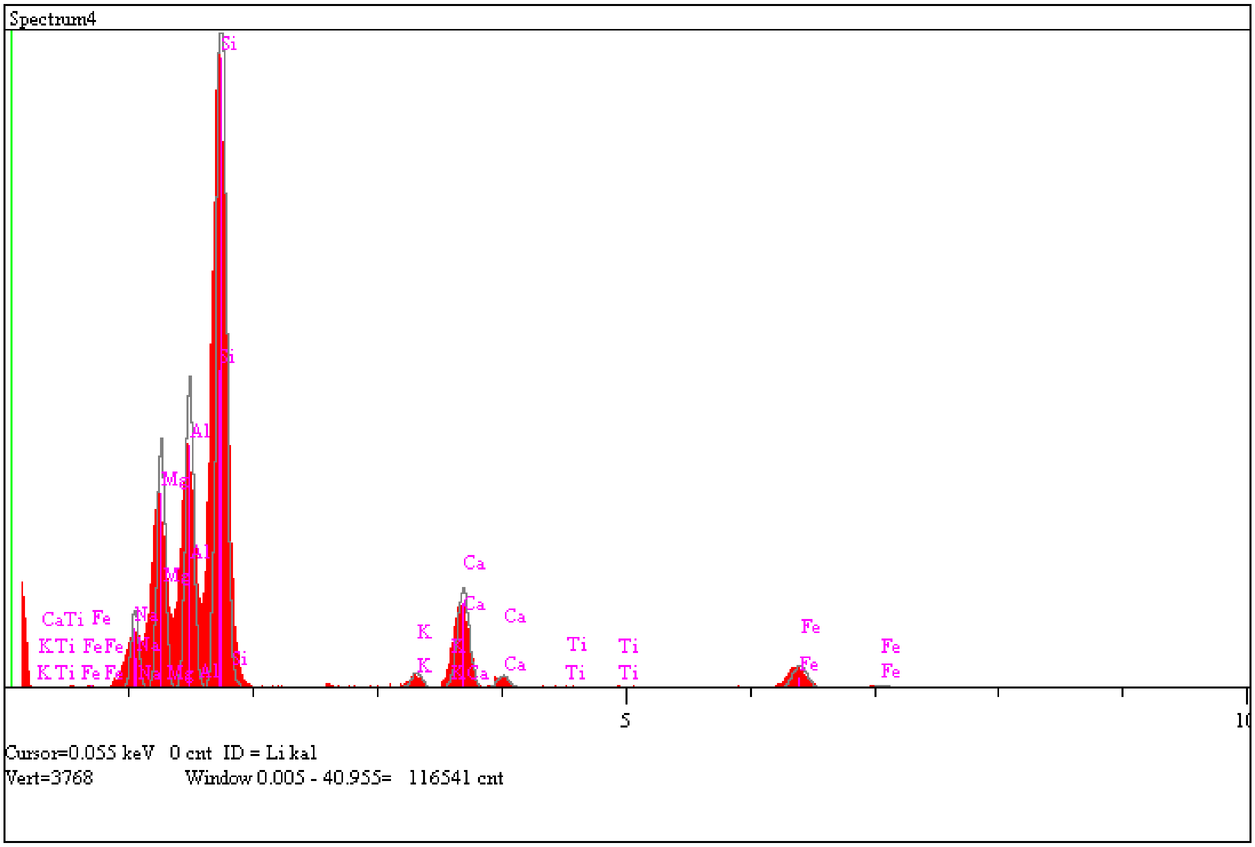

Elt.	Line	Intensity (c/s)	Error 2-sig	Atomic %	K-Ratio	
Na	Ka	54.48	1.906	4.231	0.0248	
Mg	Ka	186.45	3.525	12.033	0.0870	
Al	Ka	242.70	4.022	15.208	0.1238	
Si	Ka	681.32	6.739	44.572	0.3874	
K	Ka	16.25	1.041	1.346	0.0179	
Ca	Ka	110.47	2.714	10.267	0.1431	
Ti	Ka	2.09	0.374	0.260	0.0040	
Fe	Ka	31.32	1.445	12.083	0.2120	
				100.000		Total

Model Micrometeorite 3:

- Exposure to archaeal lipids: Fig. 5A – p3
- Exposure to E.coli lipids: Fig. 5B – p3
- Exposure to plant lipids mixture: Fig. 5C – p3
- Fluorescence micrograph in Fig. 3F corresponds to this particle.

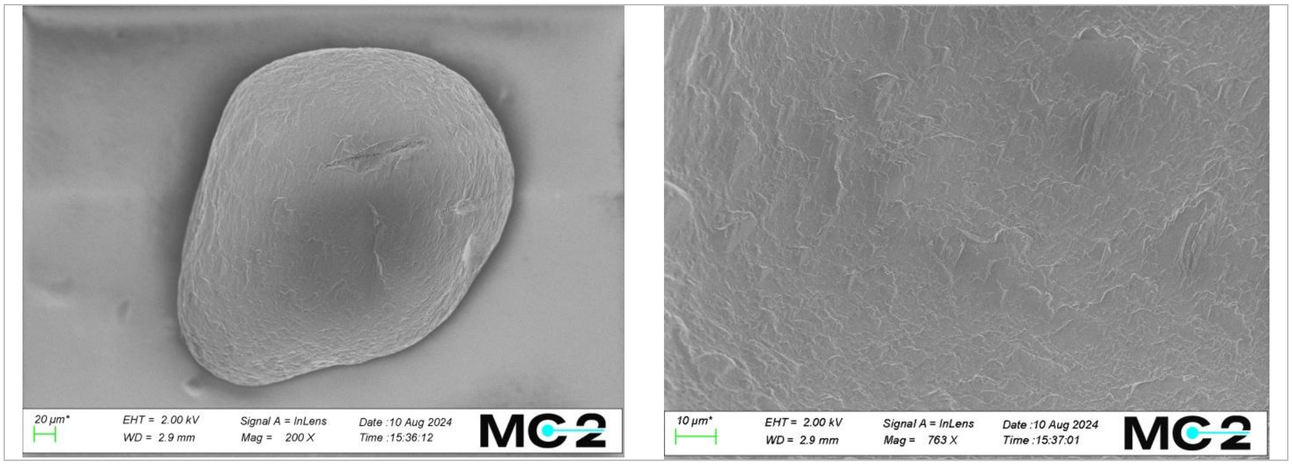

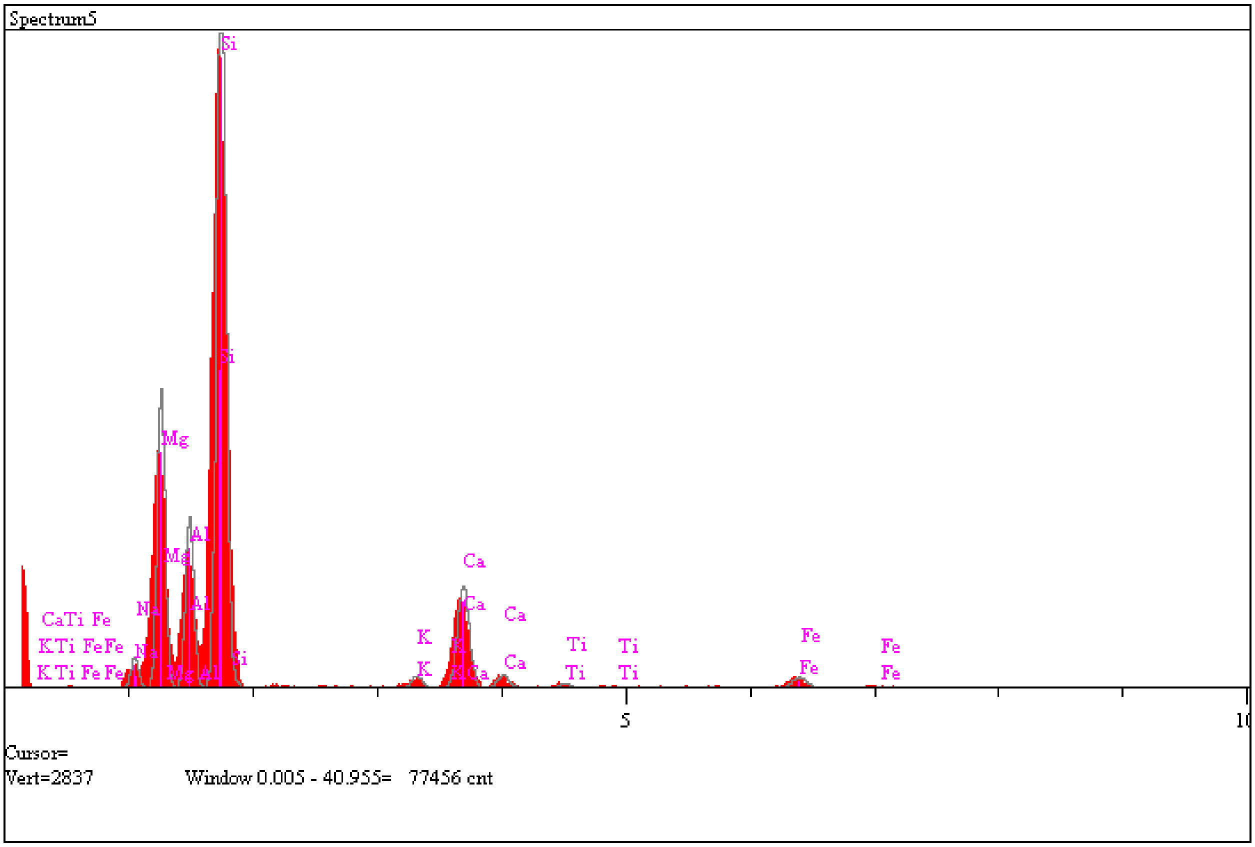

Elt.	Line	Intensity (c/s)	Error 2-sig	Atomic %	K-Ratio	
Na	Ka	16.32	1.043	1.871	0.0117	
Mg	Ka	168.88	3.355	16.159	0.1241	
Al	Ka	100.70	2.591	9.685	0.0809	
Si	Ka	511.69	5.840	50.775	0.4580	
K	Ka	9.34	0.789	1.214	0.0162	
Ca	Ka	85.28	2.384	12.478	0.1739	
Ti	Ka	4.19	0.528	0.826	0.0126	
Fe	Ka	11.52	0.876	6.993	0.1227	
				100.000		Total

Natural particle 1 (with no plasma treatment)

- Exposure to archaeal lipids, Fig. 5A - s1

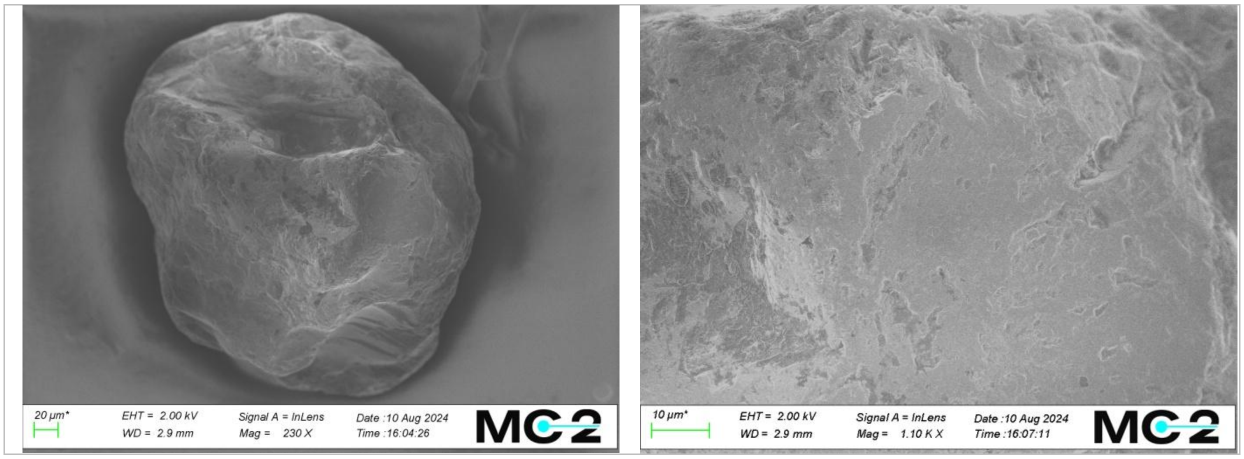

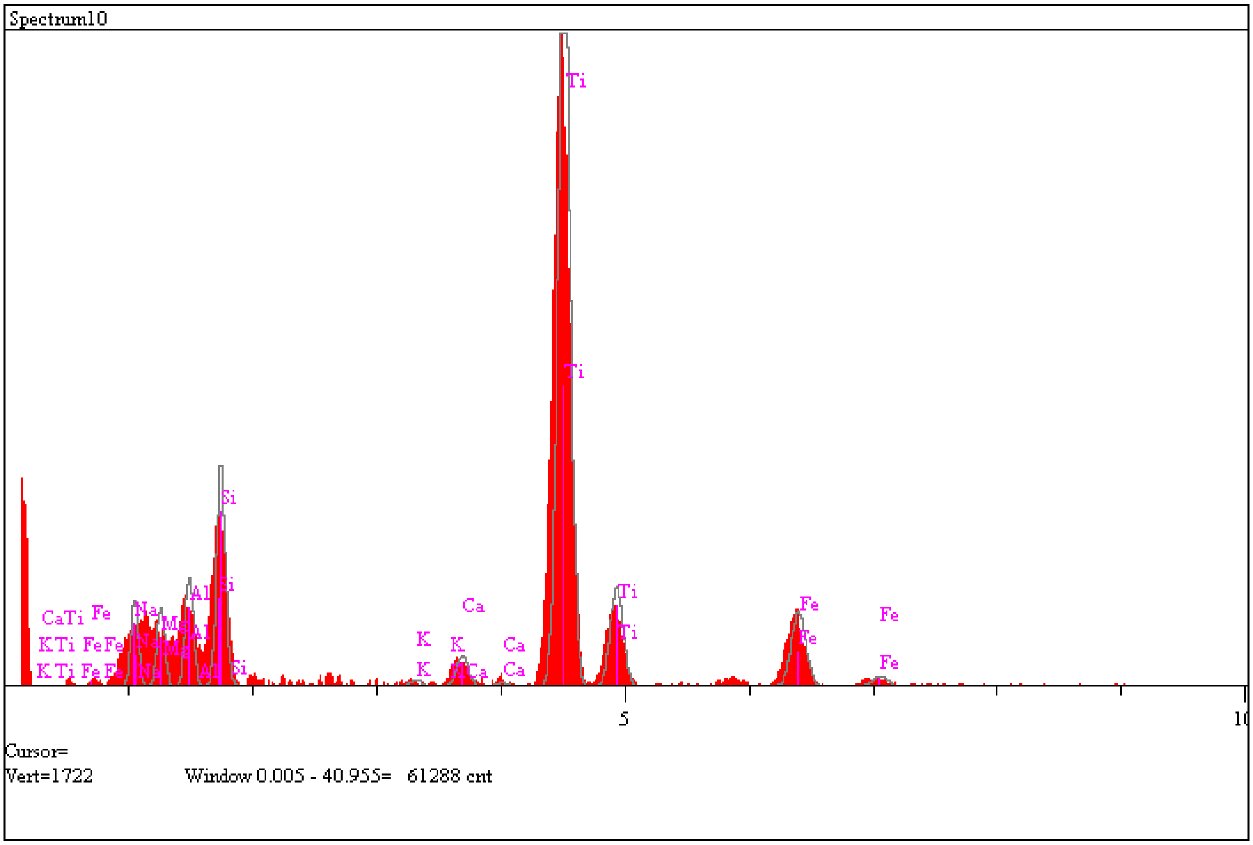

Elt.	Line	Intensity (c/s)	Error 2-sig	Atomic %	K-Ratio	
Na	Ka	27.64	1.357	3.530	0.0104	
Mg	Ka	27.05	1.343	2.658	0.0105	
Al	Ka	39.15	1.615	3.380	0.0166	
Si	Ka	83.98	2.366	6.906	0.0396	
K	Ka	3.36	0.473	0.306	0.0031	
Ca	Ka	15.95	1.031	1.537	0.0171	
Ti	Ka	400.53	5.167	59.078	0.6332	
Fe	Ka	48.03	1.789	22.606	0.2696	
				100.000		Total

Natural particle 2 (with no plasma treatment)

- Exposure to archaeal lipids, Fig. 5A – s2
- Fluorescence micrograph in Fig. 3A corresponds to this particle.

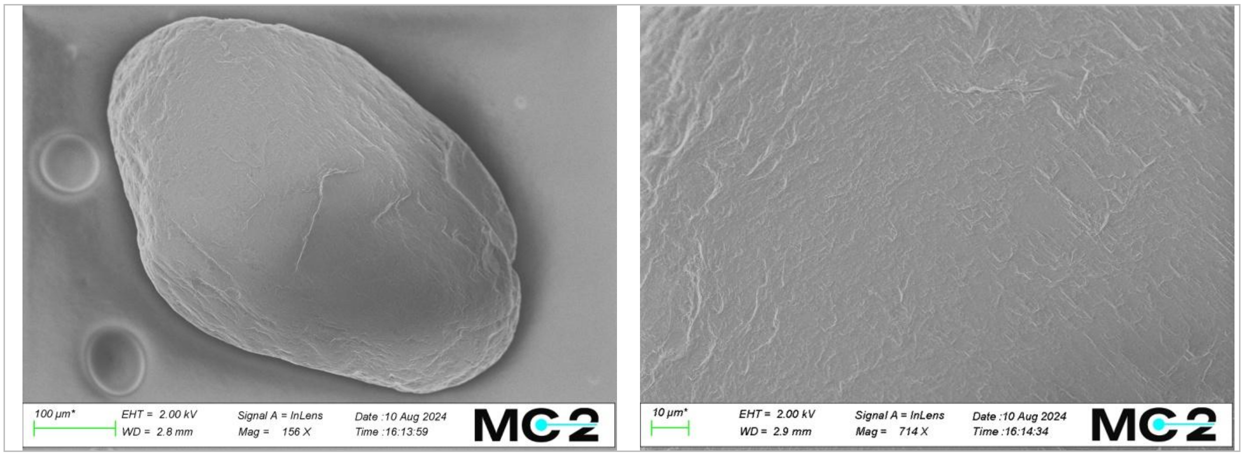

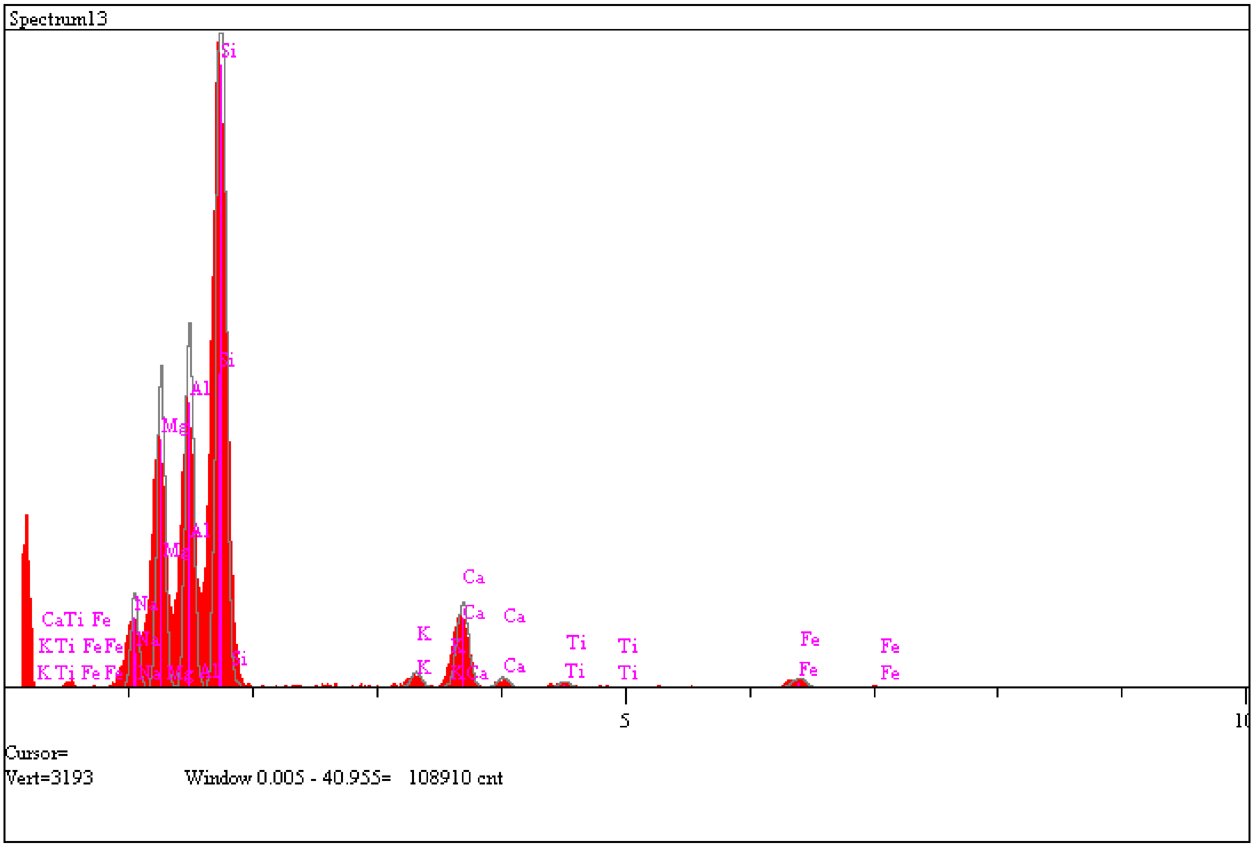

Elt.	Line	Intensity (c/s)	Error 2-sig	Atomic %	K-Ratio	
Na	Ka	56.65	1.943	4.793	0.0327	
Mg	Ka	204.25	3.690	14.846	0.1213	
Al	Ka	241.03	4.009	17.712	0.1564	
Si	Ka	579.99	6.218	45.625	0.4196	
K	Ka	14.22	0.973	1.432	0.0199	
Ca	Ka	80.40	2.315	9.080	0.1325	
Ti	Ka	6.32	0.649	0.959	0.0153	
Fe	Ka	11.87	0.889	5.554	0.1022	
				100.000		Total

Natural particle 3 (with no plasma treatment)

- Exposure to archaeal lipids, Fig. 5A – s3
- Fluorescence micrograph in Fig. 3C corresponds to this particle.

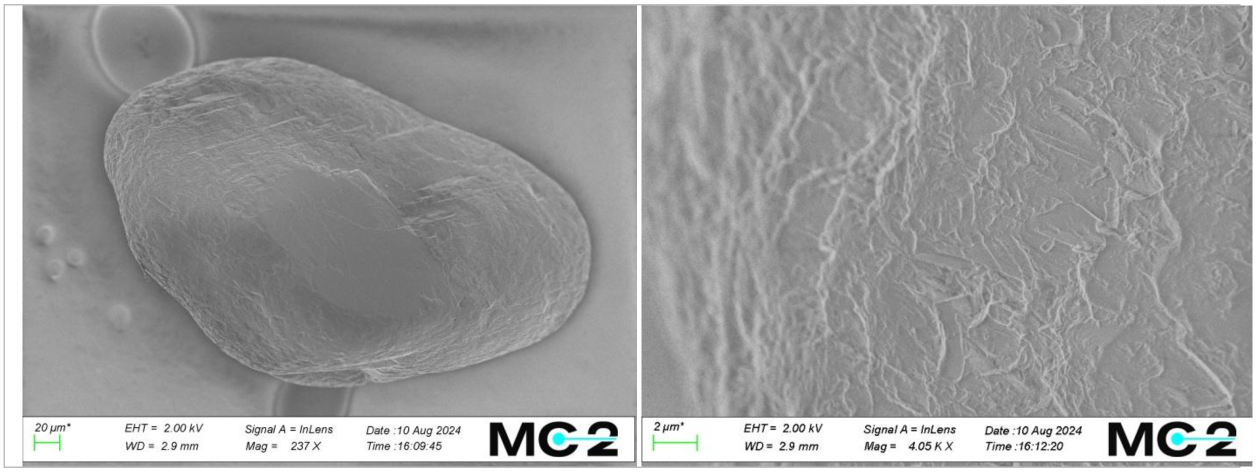

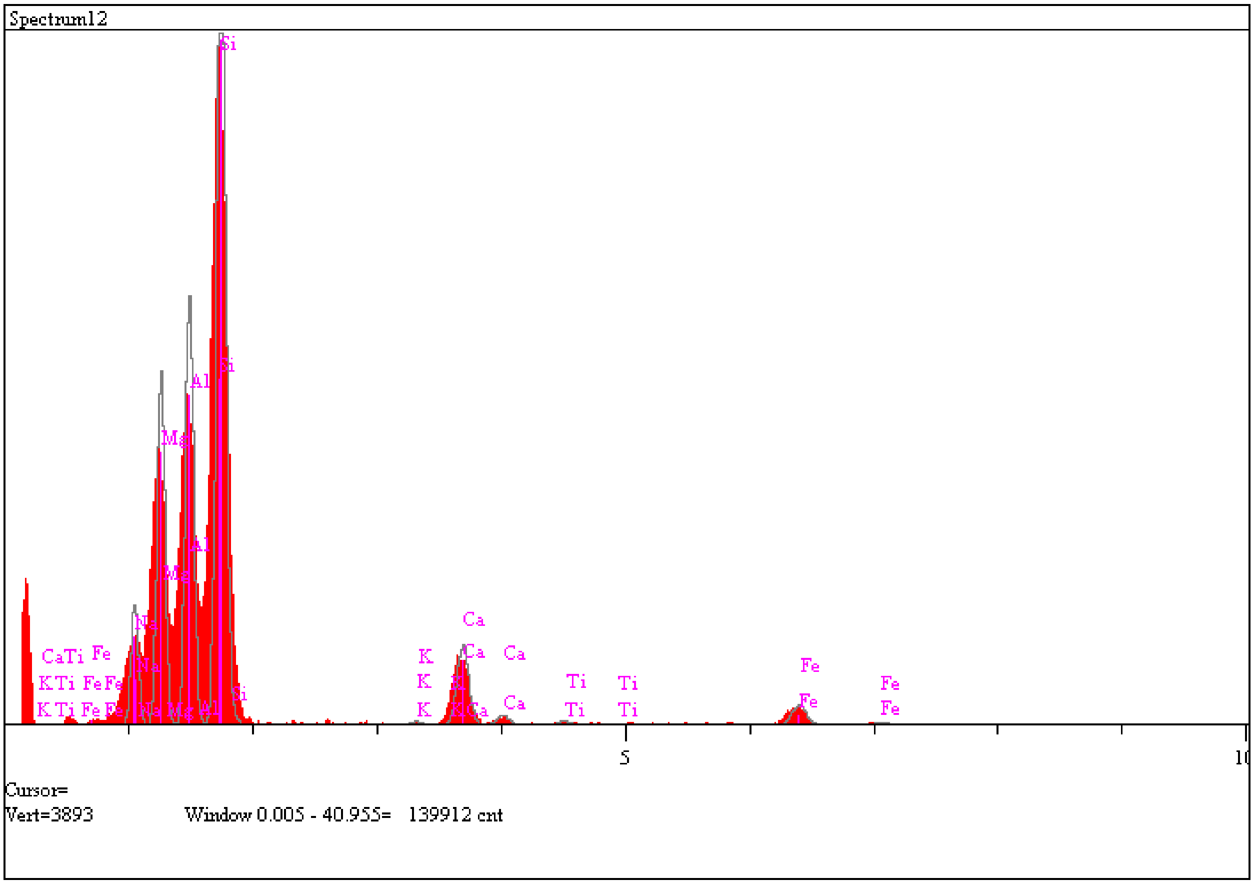

Elt.	Line	Intensity (c/s)	Error 2-sig	Atomic %	K-Ratio	
Na	Ka	82.70	2.348	5.558	0.0361	
Mg	Ka	259.90	4.162	14.850	0.1165	
Al	Ka	327.34	4.671	18.650	0.1603	
Si	Ka	713.03	6.894	43.163	0.3893	
K	Ka	3.75	0.500	0.286	0.0040	
Ca	Ka	86.38	2.399	7.327	0.1074	
Ti	Ka	4.65	0.556	0.527	0.0085	
Fe	Ka	27.37	1.351	9.640	0.1779	
				100.000		Total

Natural particle 4 (with no plasma treatment)

- Exposure to E.coli lipids, Fig. 5B – s4

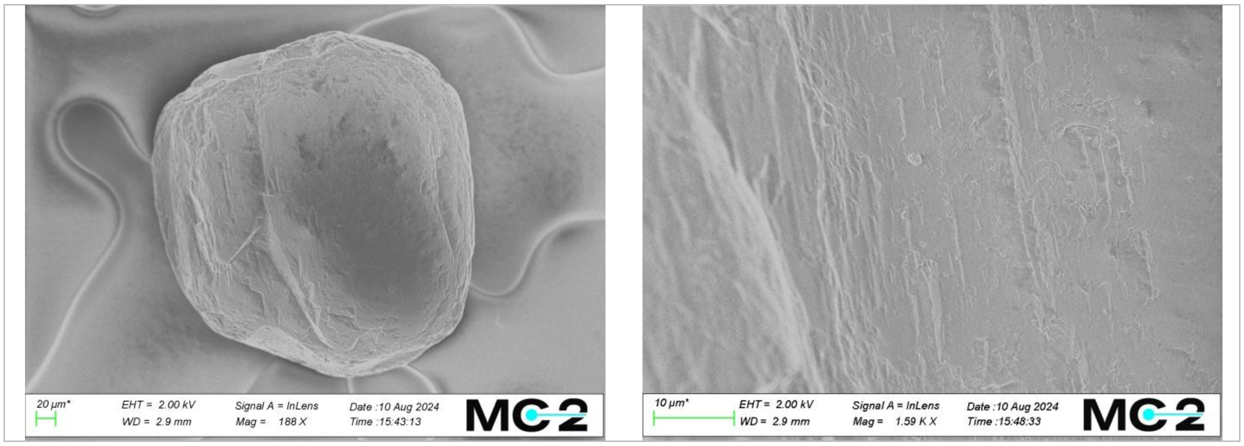

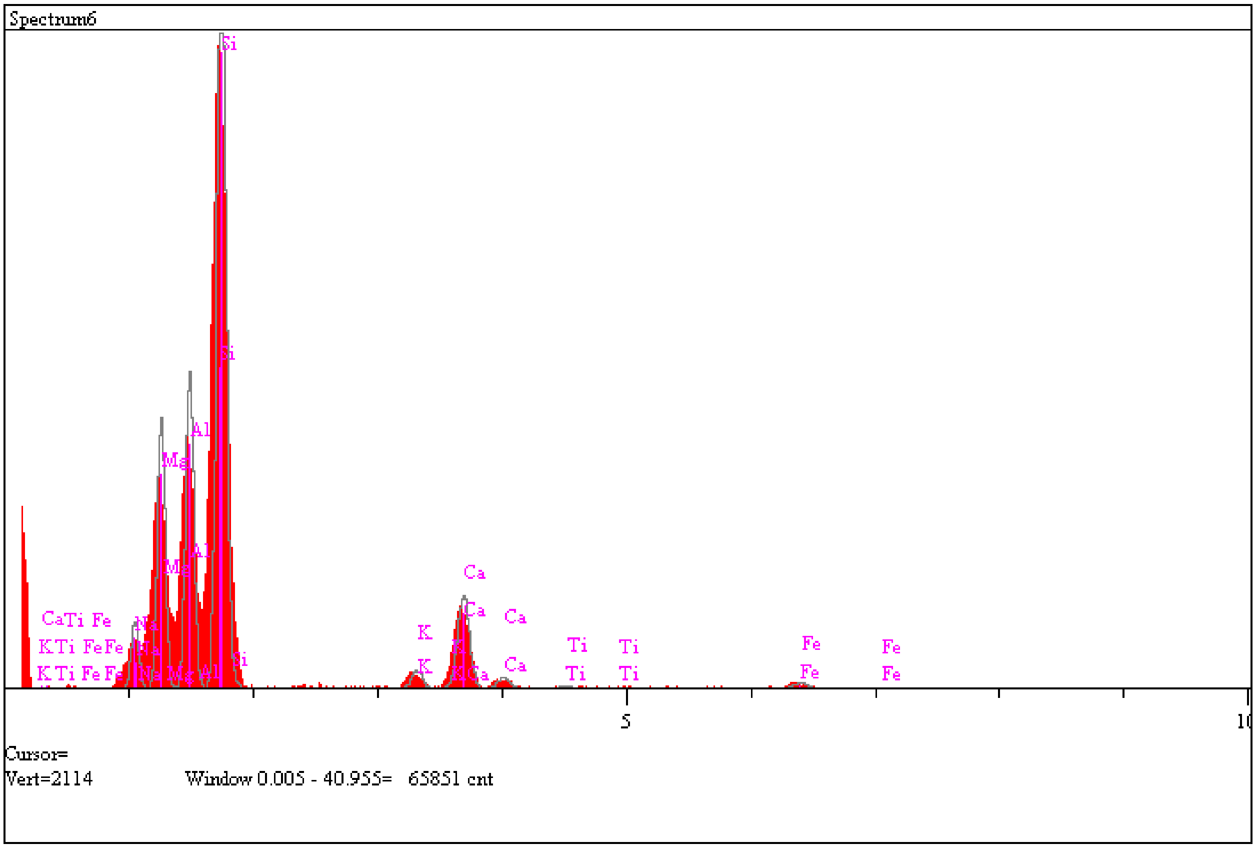

Elt.	Line	Intensity (c/s)	Error 2-sig	Atomic %	K-Ratio	
Na	Ka	26.69	1.334	3.667	0.0254	
Mg	Ka	114.05	2.757	13.462	0.1116	
Al	Ka	138.74	3.041	16.562	0.1484	
Si	Ka	385.09	5.066	49.476	0.4591	
K	Ka	10.88	0.852	1.826	0.0251	
Ca	Ka	57.68	1.961	10.885	0.1567	
Ti	Ka	1.82	0.349	0.464	0.0073	
Fe	Ka	4.69	0.559	3.658	0.0665	
				100.000		Total

Natural particle 5 (with no plasma treatment)

- Exposure to E.coli lipids, Fig. 5B – s5

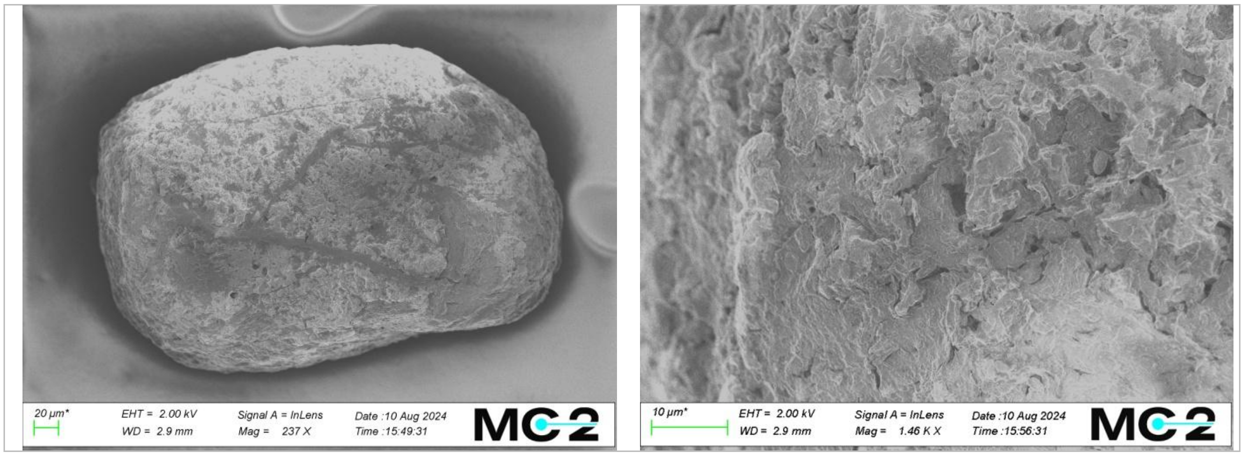

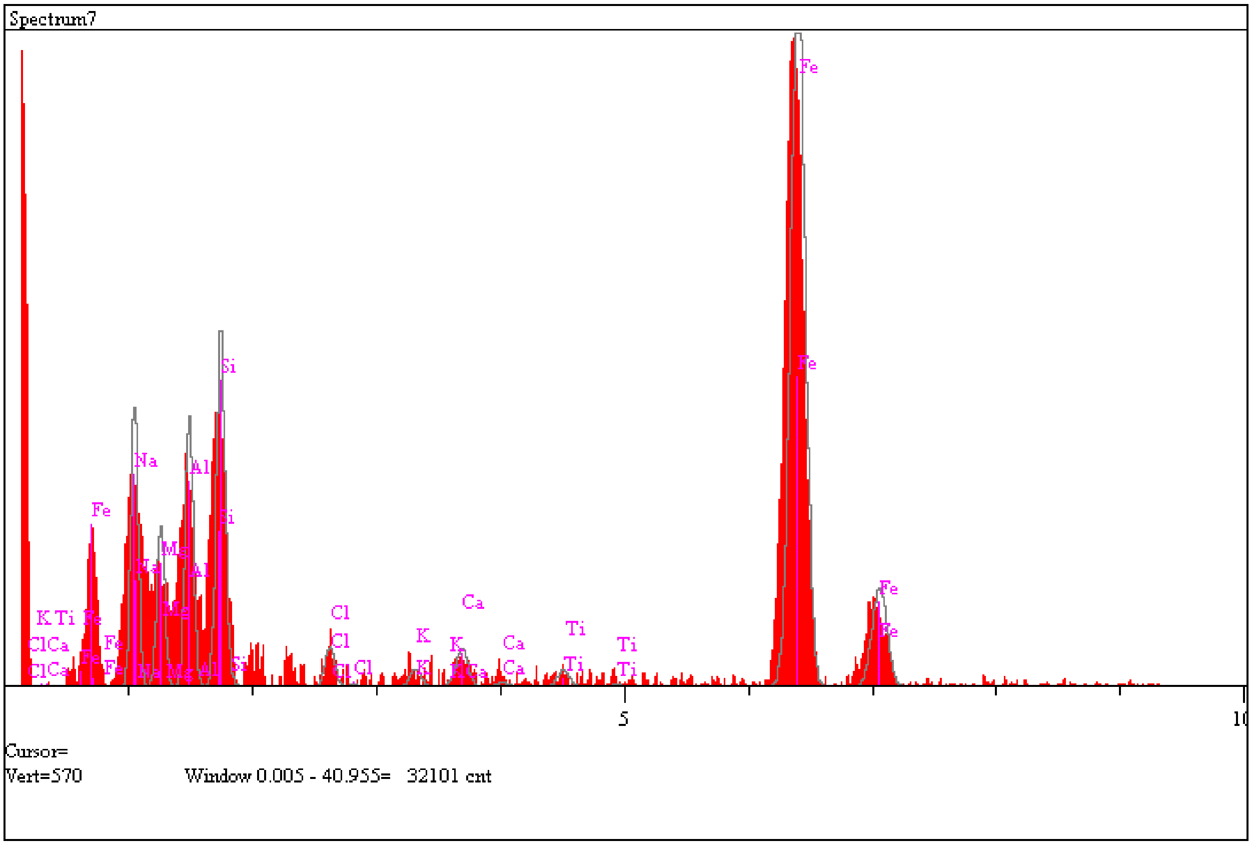

Elt.	Line	Intensity (c/s)	Error 2-sig	Atomic %	K-Ratio	
Na	Ka	30.10	1.416	5.441	0.0124	
Mg	Ka	18.24	1.103	2.456	0.0077	
Al	Ka	31.87	1.457	3.650	0.0147	
Si	Ka	44.68	1.726	4.717	0.0230	
Cl	Ka	5.72	0.617	0.586	0.0043	
K	Ka	2.86	0.437	0.331	0.0029	
Ca	Ka	6.27	0.647	0.788	0.0074	
Ti	Ka	2.92	0.441	0.442	0.0050	
Fe	Ka	150.33	3.165	81.589	0.9226	
				100.000		Total

Natural particle 6 (with no plasma treatment)

- Exposure to E.coli lipids, Fig. 5B – s6
- Fluorescence micrograph in Fig. 3B corresponds to this particle.
-

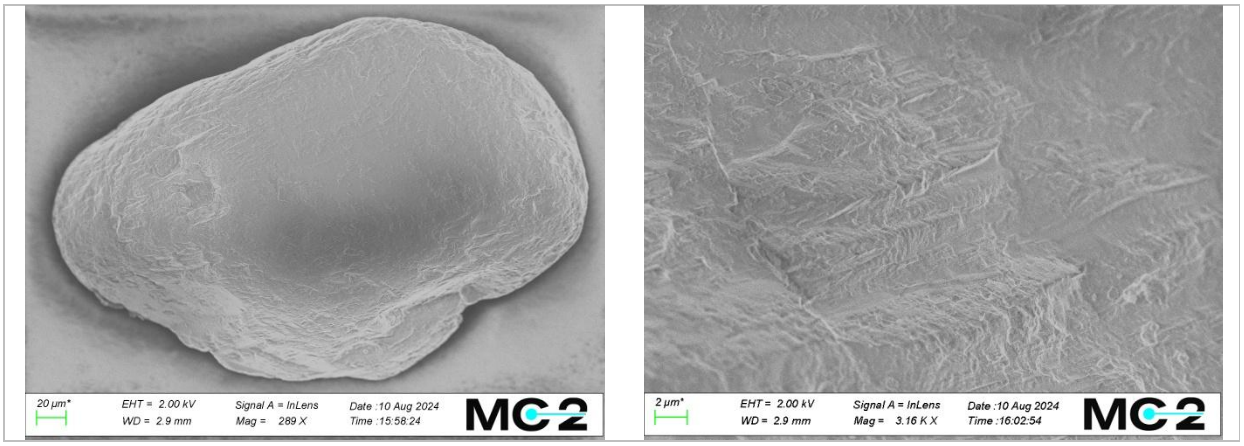

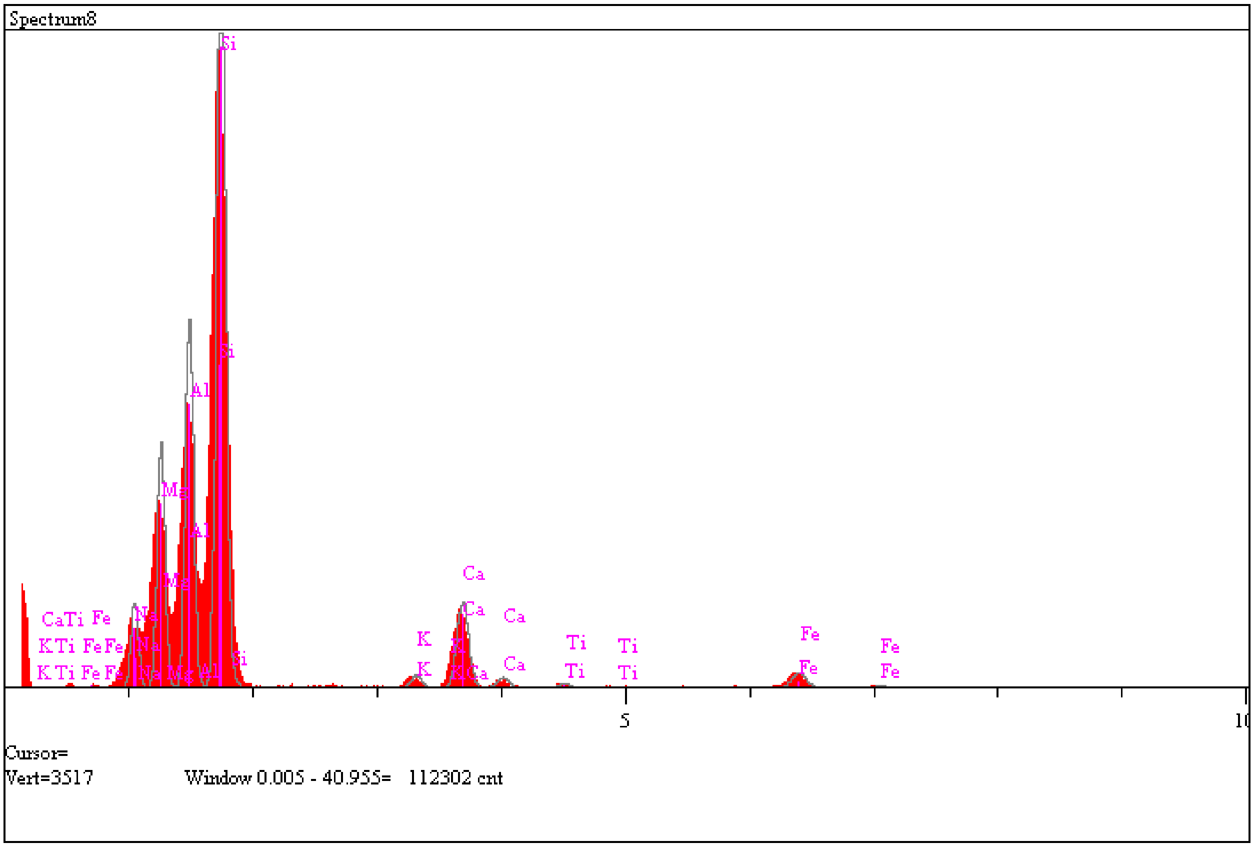

Elt.	Line	Intensity (c/s)	Error 2-sig	Atomic %	K-Ratio	
Na	Ka	56.35	1.938	4.562	0.0291	
Mg	Ka	171.84	3.384	11.768	0.0912	
Al	Ka	268.31	4.229	18.094	0.1557	
Si	Ka	639.58	6.529	46.097	0.4137	
K	Ka	12.57	0.915	1.156	0.0157	
Ca	Ka	89.12	2.437	9.178	0.1313	
Ti	Ka	5.25	0.592	0.725	0.0114	
Fe	Ka	19.72	1.146	8.421	0.1518	
				100.000		Total

Natural particle 7 (with no plasma treatment)

- Exposure to reference lipids mixture, Fig. 5C – s7

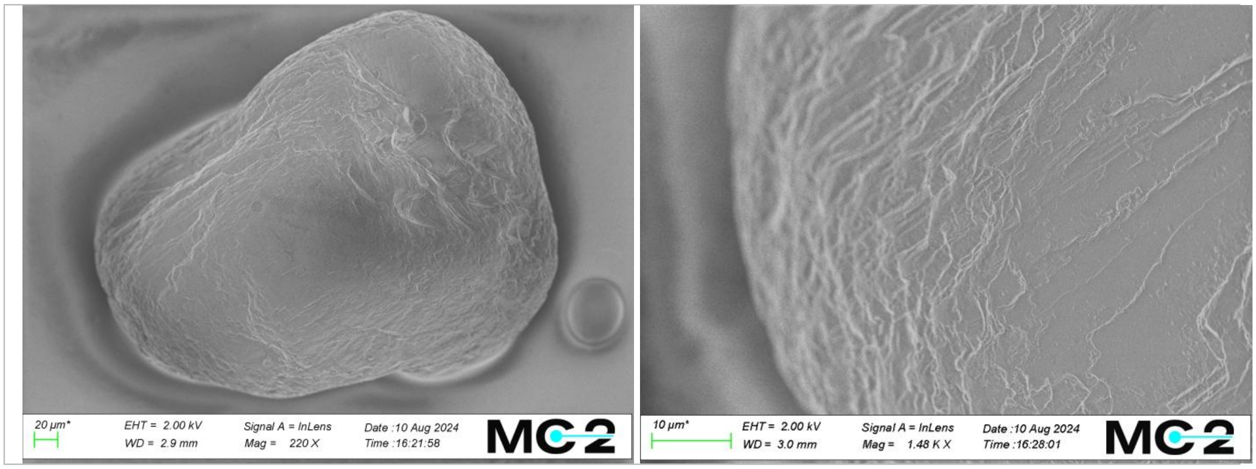

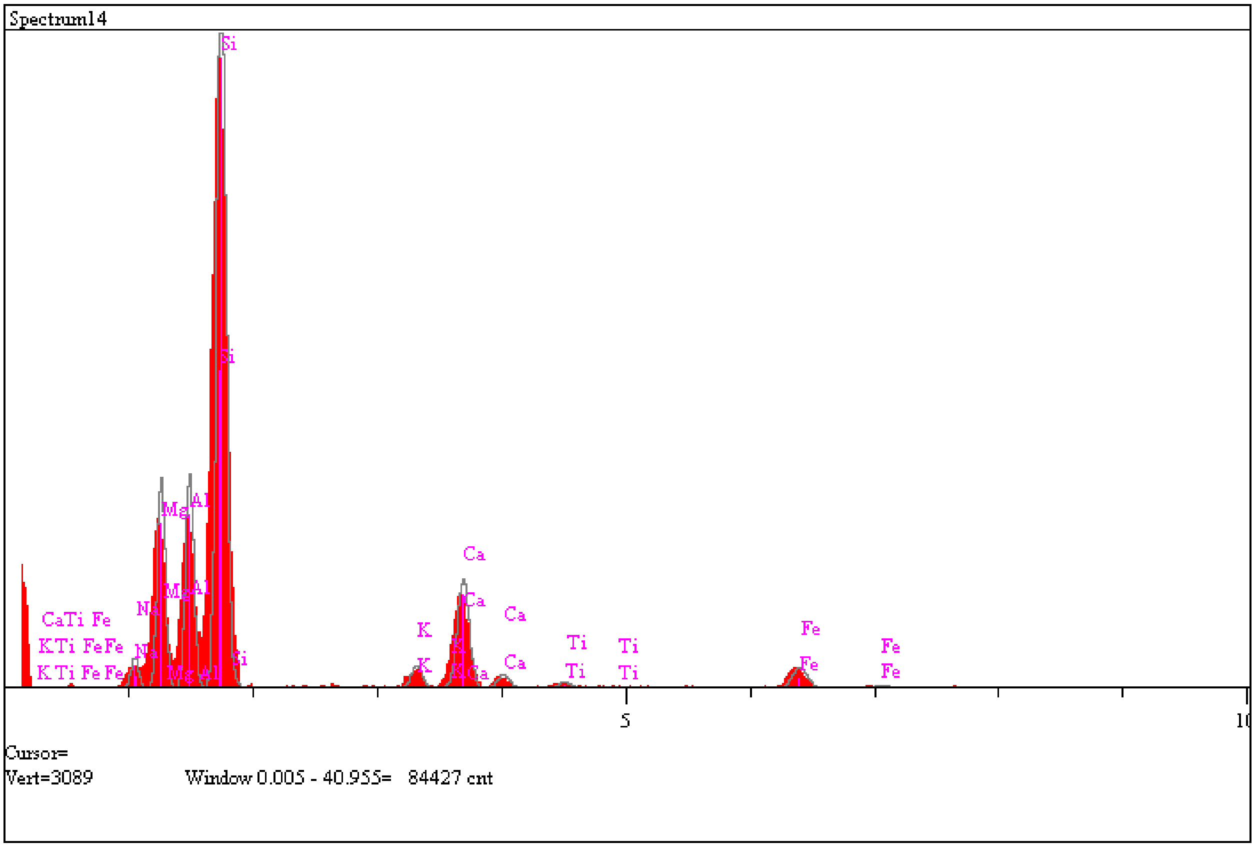

Elt.	Line	Intensity (c/s)	Error 2-sig	Atomic %	K-Ratio	
Na	Ka	17.59	1.083	1.863	0.0103	
Mg	Ka	129.19	2.935	11.169	0.0774	
Al	Ka	137.35	3.026	11.428	0.0899	
Si	Ka	556.19	6.089	47.696	0.4061	
K	Ka	19.74	1.147	2.174	0.0279	
Ca	Ka	98.52	2.563	12.228	0.1639	
Ti	Ka	6.08	0.637	1.013	0.0149	
Fe	Ka	24.12	1.268	12.430	0.2096	
				100.000		Total

Natural particle 8 (with no plasma treatment)

- Exposure to reference lipids mixture, Fig. 5C – s8

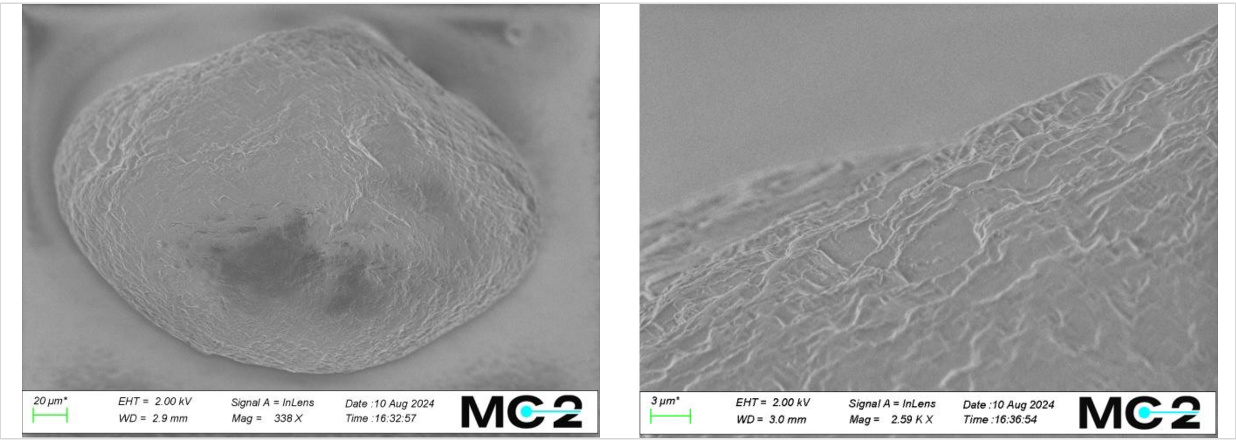

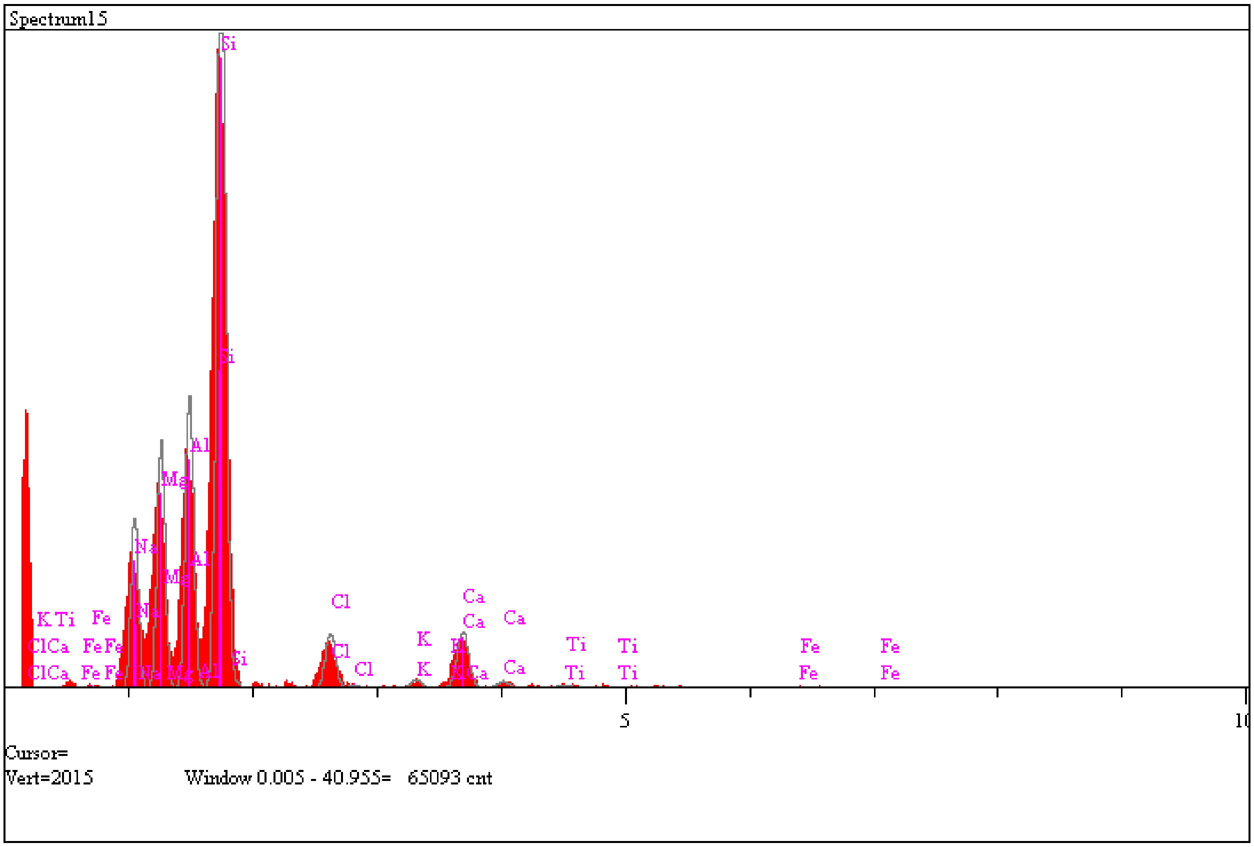

Elt.	Line	Intensity (c/s)	Error 2-sig	Atomic %	K-Ratio	
Na	Ka	64.38	2.072	8.837	0.0685	
Mg	Ka	99.00	2.569	12.344	0.1082	
Al	Ka	121.87	2.850	15.410	0.1455	
Si	Ka	364.01	4.926	49.729	0.4847	
Cl	Ka	28.19	1.371	4.546	0.0542	
K	Ka	4.83	0.567	0.887	0.0124	
Ca	Ka	33.34	1.491	6.823	0.1011	
Ti	Ka	2.14	0.378	0.587	0.0095	
Fe	Ka	0.99	0.257	0.836	0.0157	

				100.000		Total

Natural particle 9 (with no plasma treatment)

- Exposure to reference lipids mixture, Fig. 5C – s9

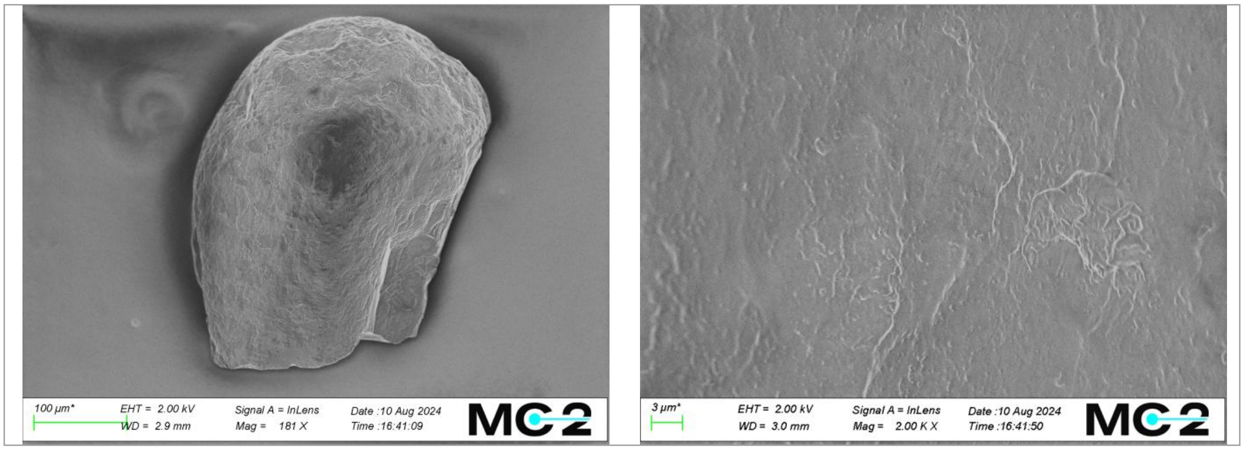

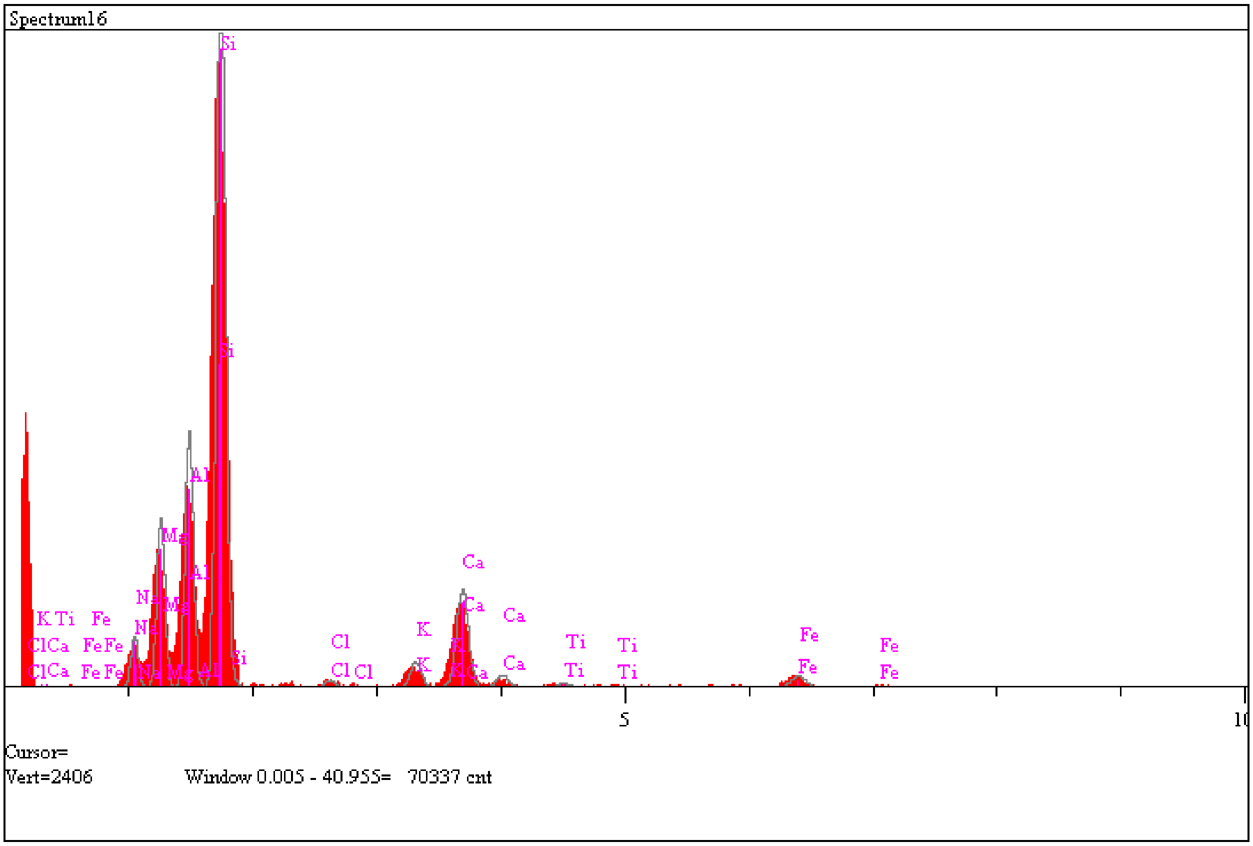

Elt.	Line	Intensity (c/s)	Error 2-sig	Atomic %	K-Ratio	
Na	Ka	22.89	1.235	3.113	0.0190	
Mg	Ka	81.32	2.328	9.250	0.0694	
Al	Ka	127.43	2.914	14.064	0.1188	
Si	Ka	430.03	5.354	50.229	0.4470	
Cl	Ka	4.41	0.542	0.598	0.0066	
K	Ka	16.94	1.063	2.591	0.0341	
Ca	Ka	68.85	2.142	11.865	0.1630	
Ti	Ka	3.30	0.469	0.765	0.0115	
Fe	Ka	10.55	0.839	7.525	0.1306	

				100.000		Total

3. Table of lipid interaction with mineral surfaces

Lipid type	Headgroup Charge at pH 7	Associated lipid mixture(s)	Surface interaction mechanism	Mineral surface affinity	Ref.
16:0 Diether PC	zwitterionic	Archaeal	No previous direct study		
PE	zwitterionic	E.coli, Soy bean	Electrostatic attraction and hydrogen bonding	Goethite (α-FeO(OH)), hematite (Fe_2O_3)	[1]
PG	negative	E.coli	(DMPG) interacts via the hydroxyl groups of the terminal glycerol where they were oppositely charged (at pH:2-6.6)	Titanium dioxide (TiO_2)	[2]
Cardiolipin	Negative	*E. coli*	No previous direct study		
PC	zwitterionic	Soy bean	Interaction through the phosphate group of the lipid head group	Pyrite (FeS_2)	[3]
PC	zwitterionic	Soy bean	(DTPC) van der Waals and electrostatic forces between the oxide surface and the negatively charged ($-R(PO_4^-)R'-$) portion of the phosphocholine headgroup.	quartz (α-SiO_2), rutile (α-TiO_2), and corundum (α-Al_2O_3)	[4]
PC	zwitterionic	Soy bean	dipalmitoylphosphatidylcholine (DPPC), via effects of surface charge and Ca^{2+} ions	Sapphire (α-Al_2O_3)	[5]
PC	zwitterionic	Soy bean	individual lipids within the membrane juts out and makes links with the support	mica	[6]
PI	negative	Soy bean	No previous direct study		
PA	negative	Soy bean	formed **inner-sphere complexes** through P–O–Fe bonds, strong, specific adsorption to iron oxides	goethite and hematite	[1]

References

1. Cagnasso, M.; Boero, V.; Franchini, M. A.; Chorover, J., ATR-FTIR studies of phospholipid vesicle interactions with alpha-FeOOH and alpha-Fe2O3 surfaces. *Colloids and surfaces. B, Biointerfaces* **2010,** *76* (2), 456-67.
2. Le, Q.-C.; Ropers, M.-H.; Terrisse, H.; Humbert, B., Interactions between phospholipids and titanium dioxide particles. *Colloids and Surfaces B: Biointerfaces* **2014,** *123*, 150-157.
3. Zhang, X.; Borda, M. J.; Schoonen, M. A. A.; Strongin, D. R., Adsorption of Phospholipids on Pyrite and Their Effect on Surface Oxidation. *Langmuir* **2003,** *19* (21), 8787-8792.
4. Xu, J.; Stevens, M. J.; Oleson, T. A.; Last, J. A.; Sahai, N., Role of Oxide Surface Chemistry and Phospholipid Phase on Adsorption and Self-Assembly: Isotherms and Atomic Force Microscopy. *The Journal of Physical Chemistry C* **2009,** *113* (6), 2187-2196.
5. Oleson, T. A.; Sahai, N.; Wesolowski, D. J.; Dura, J. A.; Majkrzak, C. F.; Giuffre, A. J., Neutron reflectivity study of substrate surface chemistry effects on supported phospholipid bilayer formation on (112¯0) sapphire. *Journal of Colloid and Interface Science* **2012,** *370* (1), 192-200.
6. Pertsin, A.; Grunze, M., Possible mechanism of adhesion in a mica supported phospholipid bilayer. *The Journal of Chemical Physics* **2014,** *140* (18).
